# Formation, Reactivity and Decomposition of Aryl Phospha‐Enolates

**DOI:** 10.1002/chem.202203081

**Published:** 2022-12-15

**Authors:** Stephanie J. Urwin, Jose M. Goicoechea

**Affiliations:** ^1^ Department of Chemistry University of Oxford 12 Mansfield Road Oxford OX1 3TA United Kingdom

**Keywords:** decarbonylation, phospha-enolates, phosphines, phosphorus, 2-phosphaethynolate

## Abstract

Two lithium phospha‐enolates [RP=C(Si^*i*^Pr_3_)OLi]_2_ were prepared by reaction of triisopropyl silyl phosphaethynolate, ^*i*^Pr_3_SiPCO, with aryl lithium reagents LiR (R=Mes: 1,3,5‐trimethyl phenyl; or Mes*: 1,3,5,‐tri‐tertbutyl phenyl). Monomer/dimer aggregation of the enolates can be modulated by addition of 12‐crown‐4. Substitution of lithium for a heavier alkali metal was achieved through initial formation of a silyl enol ether, followed by reaction with KO^*t*^Bu to form the corresponding potassium phospha‐enolate [MesP=C(Si^*i*^Pr_3_)OK]_2_. On addition of water, the enolates are protonated to afford RP=C(Si^*i*^Pr_3_)(OH). For the sterically less demanding system (R=Mes), this phospha‐enol rapidly tautomerises to the corresponding acyl phosphine MesP(H)C(Si^*i*^Pr_3_)(O), which on heating extrudes CO. In contrast, bulkier phospha‐enol (R=Mes*) is stable to rearrangement at room temperature and thermally decomposes to RH and ^*i*^Pr_3_SiPCO.

## Graphical abstract

**The synthesis and reactivity** of two lithium phospha‐enolates [RP=C(Si^*i*^Pr_3_)OLi]_2_ (R=Mes, Mes*) is described. Derivatization at the oxygen atom with electrophiles is possible, facilitating subsequent reactivity pathways including, for example, spontaneous decarbonylation.

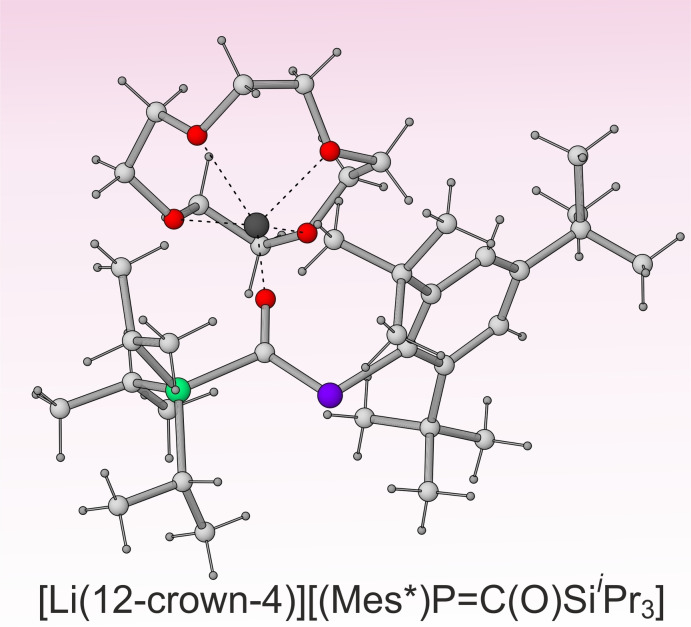

## Introduction

Lithium enolates are integral synthetic intermediates with wide ranging applications.^[1]^ A key characteristic that drives the diverse reactivity of the enolate functionality is 1,3‐delocalisation of negative charge between the β‐carbanion and the adjacent carbonyl. Modification of this well‐established functional group by replacing this C−H group with a diagonally‐related phosphorus atom, to create a phospha‐enolate, has the potential to disrupt this intramolecular interaction. This modification can significantly alter resonance stabilization and hence, reactivity.

**Figure 1:**
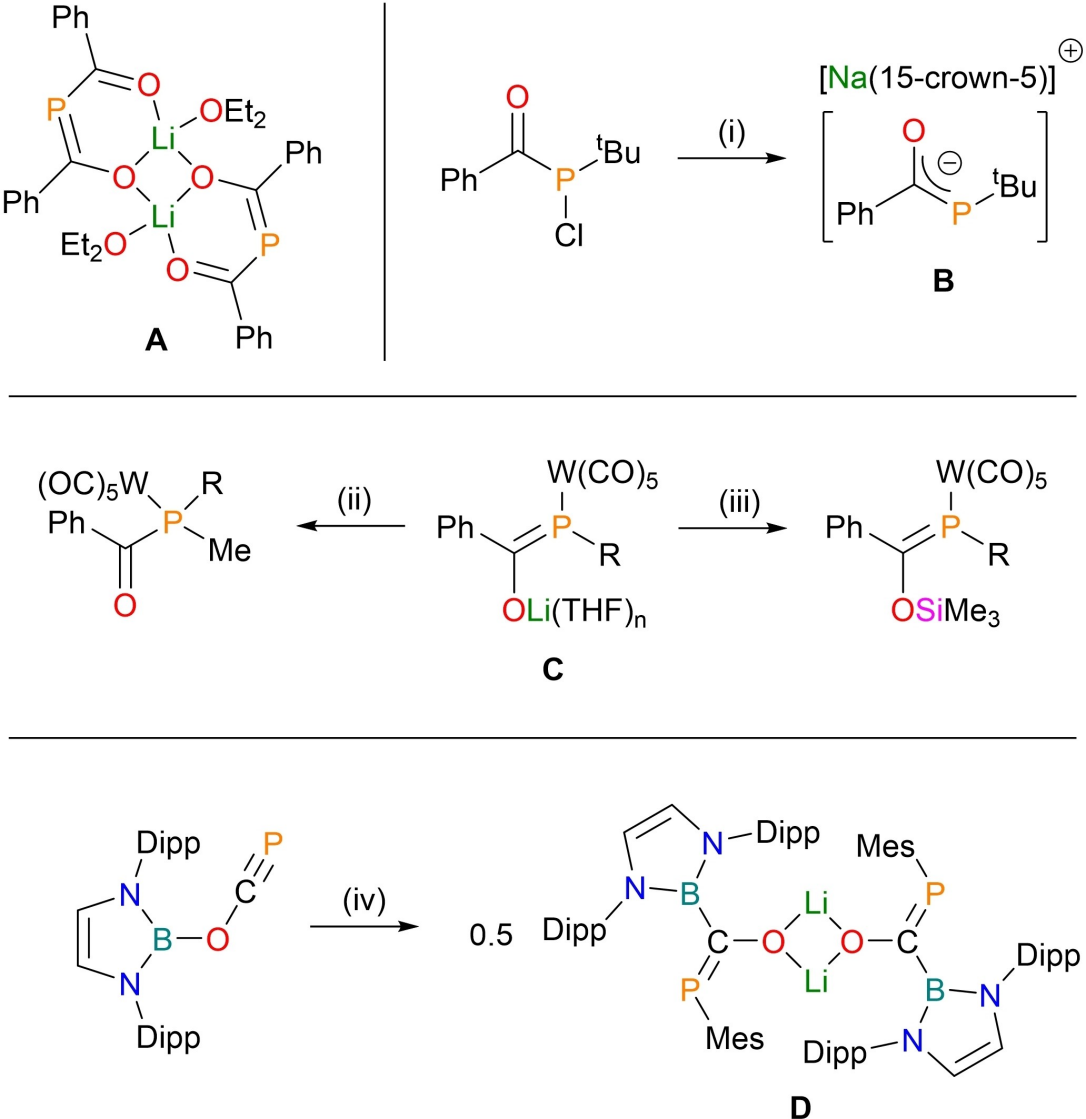
Previous examples of alkali‐metal phospha‐enolates. R=C(H)(SiMe_3_)_2_; Mes=1,3,5‐trimethyl phenyl; Dipp=1,5‐diisopropyl phenyl. (i) Na(napthalene), 15‐crown‐5, – napthalene, – NaCl; (ii) MeI, – LiCl; (iii) Me_3_SiCl; – LiCl; (iv) LiMes.

Initial steps to alkali‐metal phospha‐enolates included the addition of lithium phosphide to phenyl acyl chloride to form a 2‐phospha‐1,3‐dionate **A**, in a complex sequence of steps relying on the tautomerisation of a bis‐acyl phosphine (Figure 1).^[2]^ Dimeric in the solid state, the three central heterocycles are not co‐planar, indicating no delocalisation throughout the molecule. Dehalogenation of a mono‐acyl phosphine produced a sodium phospha‐enolate **B**, which was shown to react as a phosphide despite the fact that the negative charge is distributed equally over the oxygen and phosphorus atoms.^[3]^ A similar synthetic approach resulted in the formation of phospha‐enolate **C**.^[4]^ The reactivity of this tungsten‐supported example varies depending on the nature of the added substrate; MeI gives rise to phosphorus‐centered alkylation, where Me_3_SiCl results in silylation of the oxygen atom.

The phosphaethynolate anion (PCO^−^)^[5]^ has been shown to act an effective chemical precursor to a number of phosphorus‐containing compounds including phosphinecarboxamides (H_2_PC(O)NR(H)),^[6]^ and more recently, metal cyaphido‐complexes ([M]−C≡P).[^7^, ^8^] It can also be employed as a precursor to phospha‐enolates. The addition of an organo‐lithium to a boryl‐substituted phosphaethynolate produced a structurally authenticated lithium phospha‐enolate **D**,^[9]^ a reaction that includes a migration of the supporting boryl group from the oxygen to the carbon atom.

Here, we present an extension of this reactivity to the reaction of a silyl phosphaethynolate with aryl lithium reagents to form silyl‐substituted lithium phospha‐enolates. Furthermore, we explore the fundamental reactivity of these phospha‐enolates in salt metathesis and hydrolysis reactions, as well as their thermal stability and decomposition pathways.

## Results and Discussion

**Scheme 1:**
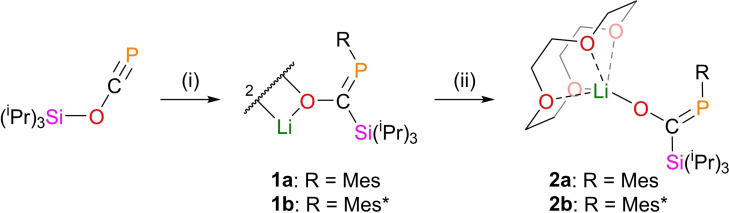
Summary of lithium enolate formation. Mes=2,4,6‐trimethyl phenyl, Mes*=2,4,6‐tri‐tertbutyl phenyl. (i) RLi, toluene, 18 h; (ii) 12‐crown‐4, toluene, 3 h.

Addition of an aryl‐lithium reagent LiR (R=Mes or Mes*) to an *in situ* generated solution of the silyl phosphaethynolate ^*i*^Pr_3_SiOCP in toluene leads the formation of lithium phospha‐enolates **1 a** and **1 b** (Scheme 1).^[10]^ The reduction of the C≡P bond order is immediately evident by ^31^P NMR spectroscopy, where the starting phosphaethynolate resonance (^31^P{^1^H}: −360 ppm) is replaced by a single new downfield signal (^31^P{^1^H} **1 a**: 132.0 ppm; **1 b**: 159.5 ppm), indicating quantitative product formation. The aryl group adds to the phosphorus center, as evidenced by the presence of C−P coupling in the ^13^C{^1^H} NMR resonance attributed to the *ipso*‐carbon atom (**1 a**: ^13^C{^1^H}: 137.55 ppm; d, ^1^ *J*_C‐P_=58.9 Hz). The silyl group was found to migrate from oxygen to carbon. Micro‐solvation by coordinating solvents is typical in the isolation of lithium enolates,^[11]^ however whilst dioxane is present during the synthesis of **1 a** and **1 b**, ^1^H NMR spectroscopy confirms none is present in the isolated products; both are base‐free enolates.

**Figure 2:**
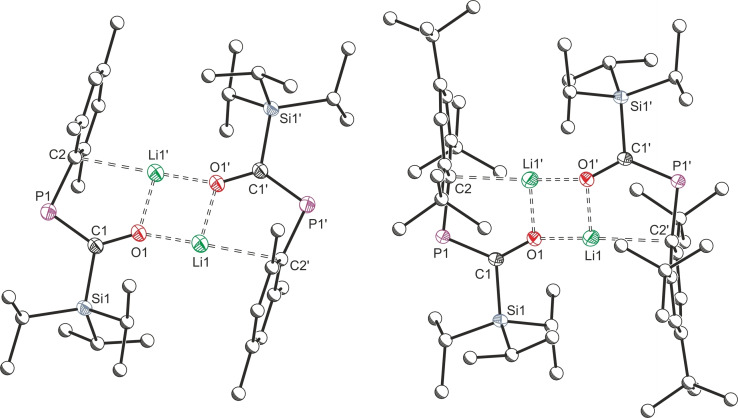
Single crystal X‐ray structures of **1 a** (left) and **1 b** (right). Hydrogen atoms are omitted and atoms of Mes and Mes* substituents are pictured as spheres of arbitrary radius. Ellipsoids are shown at 50 % probability.

Lithium enolates **1 a** and **1 b** readily crystallize from standing hexane solutions; Figure 2 depicts the resulting solid‐state structures, and key bond metrics are collated in Table 1. Like the direct boryl analogue **D**, both are dimeric enolates, featuring a Li_2_O_2_ 4‐membered ring in the center of a fused planar tricyclic system. The internal Li−O−Li angles of this planar motif are close to 90°. Short Li1⋅⋅⋅C2 contacts in both **1 a** (2.412(2) Å) and **1 b** (2.422(2) Å) indicate interaction of the cation with the aryl group, providing intramolecular stabilization in the absence of an external coordinated base. The P=C bond lengths of **1 a** and **1 b** are 1.712(1) Å and 1.714(1) Å, which are slightly elongated for phosphaalkene bonds indicative of delocalisation of electrons from the P=C into the C−O bond.^[12]^ The magnitude of this interaction can be calculated using Natural Bond Order (NBO) analysis.^[13]^ Such analysis for **1 b** gives an interaction between the oxygen‐based lone pair and the P−C π* orbital of 272.3 kJ mol^−1^ (see Supporting Information for full details), a value consistent with moderate delocalization across the P−C−O unit.

**Table 1:** Comparative bond lengths (Å) from solid‐state structures.

	M⋅⋅⋅O1^[a]^	C1−O1	C1−P1	P1−C2	M⋅⋅⋅C2
**1 a**	1.824(3)	1.321(2)	1.714(2)	1.851(2)	2.412(3)
**1 b**	1.815(2)	1.323(2)	1.712(2)	1.881(2)	2.422(2)
**2 b**	1.771(4)	1.279(3)	1.731(2)	1.878(2)	4.344(5)
**3 b**	n.a.	1.352(2)	1.687(1)	1.857(1)	n.a.
**4**	2.602(2)	1.291(2)	1.739(2)	1.852(2)	3.056(2)

[a] Value stated is for the shortest contact.

Analogous products could not be isolated from reactions of ^*i*^Pr_3_SiOCP with LiTipp or Li^Tipp^Ter (Tipp=2,4,6‐tri‐isopropyl phenyl; Ter=2,6‐bis(2.6‐diisopropylphenyl)phenyl). The phosphaethynolate ^*i*^Pr_3_SiOCP is a transient species, and isomerizes to the corresponding phosphaketene ^*i*^Pr_3_SiPCO over time in solution.^[14]^ Addition of LiMes to a toluene solution containing only the thermodynamically favoured phosphorus‐bonded isomer gave a complex, intractable mixture of products, implying that the formation of enolates **1 a**/**1 b** occur via the oxygen‐bonded isomer. Similarly, using the triphenylsilyl analogue of the phosphaethynolate, which is known to exist as a mixture of Ph_3_SiOCP and Ph_3_SiPCO, gave an intractable mixture of products. We therefore hypothesize that formation of **1 a**/**1 b** involves initial addition of LiR across the C≡P bond of ^*i*^Pr_3_SiOCP to form an unobserved intermediate. This is immediately followed by a 1,2 migration of the ‐Si^*i*^Pr_3_ group from oxygen to carbon, driven by the oxophilicity of the lithium counter‐cation. This reactivity is analogous to what we have previously observed for a boryl‐substituted phosphaethynolate on reaction with aryl‐lithium reagents.^[9]^

**Figure 3:**
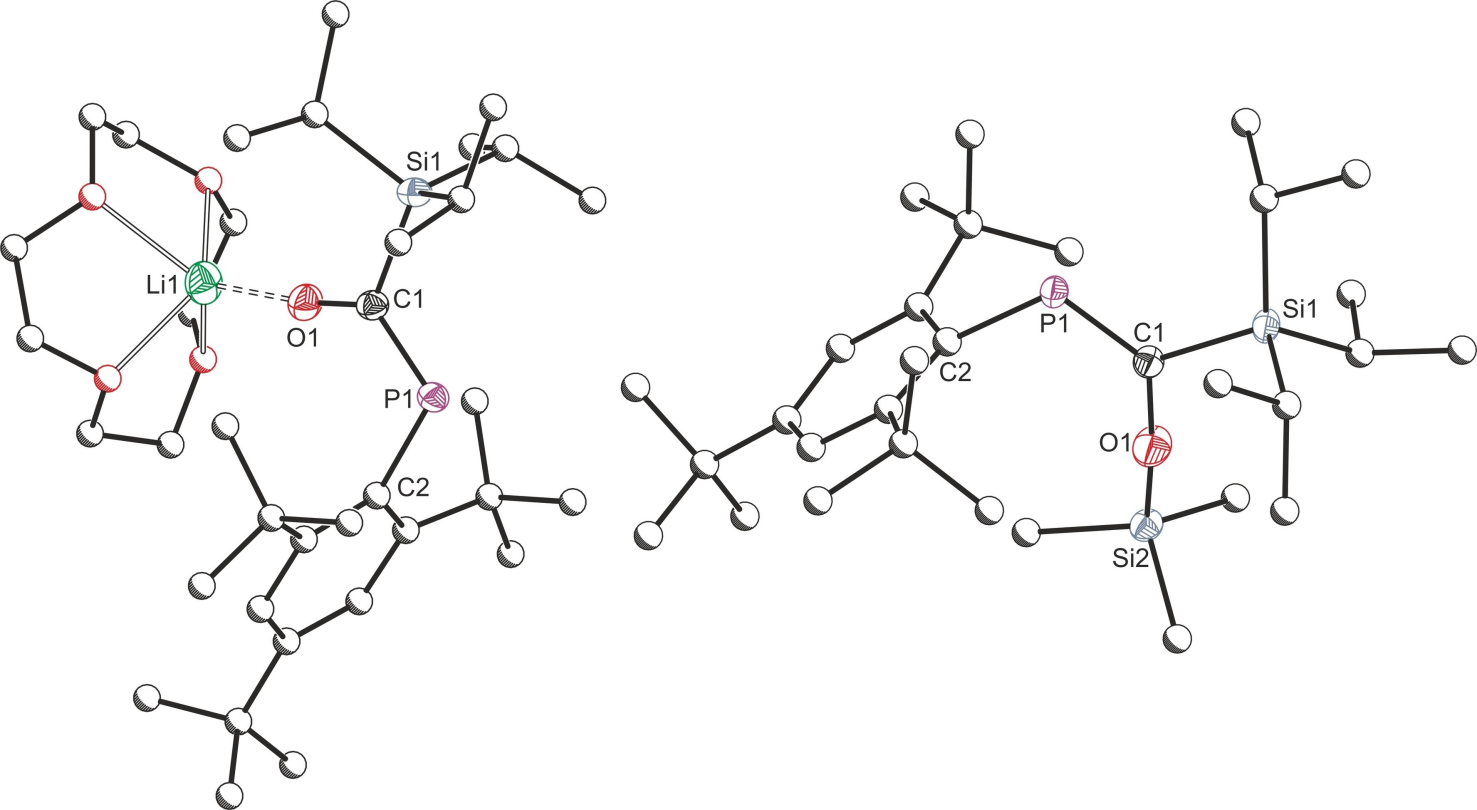
Single crystal X‐ray structures of **2 b** (left) and **3 b** (right). Hydrogen atoms are omitted and atoms of Mes* substituents are pictured as spheres of arbitrary radius. Ellipsoids are shown at 50 % probability.

Addition of 12‐crown‐4 to **1 a** or **1 b** effectively sequesters the lithium cation, breaking up the dimeric structure to form monomeric salts **2 a** and **2 b**, respectively. An upfield shift of the ^31^P{^1^H} NMR resonance provides evidence of ether coordination (^31^P{^1^H} **1 a**: 132.0 ppm; **2 a**: 108.5 ppm. **1 b**: 159.5 ppm; **2 b**: 126.6 ppm). Single crystal X‐ray diffraction analysis of **2 b** (Figure 3) confirms that this enolate is now monomeric in the solid‐state. Coordination of the crown ether has almost doubled the Li⋅⋅⋅C2 interatomic distance (**1 b**: 2.422(2) Å; **2 b**: 4.344(5) Å), indicating there is no interaction between the aryl system and the lithium cation in **2 b**. Bonds key to the enolate structure slightly contract on cation sequestration (Li1⋅⋅⋅O1: 1.815(2) Å (**1 b**), 1.771(4) Å (**2 b**); C1−O1: 1.323(1) Å (**1 b**), 1.279(3) Å (**2 b**)), where the P=C bond length elongates slightly (**1 b**: 1.712(2) Å, **2 b**: 1.731(2) Å). Unlike in the 12‐crown‐4 analogue of **D**, the C1−O1⋅⋅⋅Li1 unit of **2 b** is almost linear (**2 b**: 173.4(2)°, **D**(12‐crown‐4): 136.5(2)°), which is likely a function of the decreased steric bulk of the Si^*i*^Pr_3_ group when compared with the bulkier boryl group. NBO analysis of **2 b** indicated the O⋅⋅⋅Li bond is ionic and that there is delocalization across the P−C−O unit. A significant interaction (354.7 kJ mol^−1^) was found between the oxygen‐based lone pair and the P−C π* orbital, much higher than the analogous interaction in **1 b** (272.3 kJ mol^−1^). Hence, **2 b** can be thought of as an ionic salt, consisting of a formal lithium cation associated with a delocalized PCO anion.

**Scheme 2:**
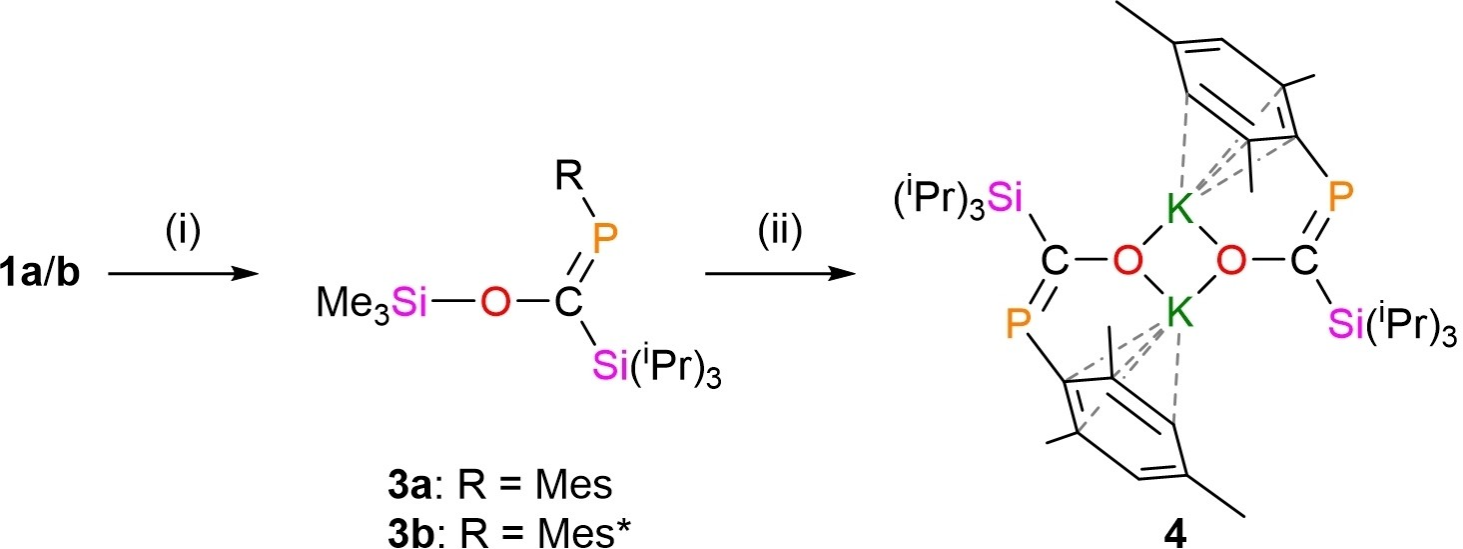
Preparation of silyl enols **3 a** and **3 b**, and the reaction of **3 a** with KO^*t*^Bu to form **4**. (i) Me_3_SiCl, toluene, 18 h, – LiCl; (ii) R=Mes, KO^*t*^Bu, toluene, 60 °C, 18 h, – Me_3_SiCl.

Addition of Me_3_SiCl to **1 a** or **1 b** results in the formation of the corresponding silylated products **3 a**/**3 b** (Scheme 2). Whilst the elimination of LiCl is typically an effective reaction driving force, here salt formation comes at the expense of disrupting a strong Li⋅⋅⋅O interaction, leading to a sluggish reaction that requires a significant stoichiometric excess of silane and 24 h to reach full conversion. A downfield shift of the ^31^P{^1^H} NMR resonances (**1 a**: 132.0 ppm, **3 a**: 190.5 ppm; **1 b**: 159.4 ppm, **3 b**: 192.2 ppm), accompanied by new upfield ^1^H NMR resonances for the alkyl groups (**3 a**: −0.09 ppm, ^2^ *J*_H‐Si_=6.7 Hz; **3 b**: −0.15 ppm, ^2^ *J*_H‐Si_=6.7 Hz) support the silylation. Expectedly due its low molecular weight and additional silyl group, compound **3 a** was isolated a pale‐yellow oil with a melting point below −30 °C. The bulkier Mes* derivative, **3 b**, can be crystallized from hexane as colorless prisms, and the resulting solid‐state structure is shown in Figure 3. The P1=C1 bond length of **3 b**, at 1.687(1) Å, is the shortest in our series here, and more the in the range expected of a true phosphaalkene.^[12]^ This greater degree of multiple bond character is also evident in the C−P coupling constants which increase relative to **2 a**/**2 b** (**3 a**: ^1^ *J*_C‐P_=93.2 Hz; **3 b**: ^1^ *J*_C‐P_=95.1 Hz). It follows that an NBO analysis indicates less delocalization over the P−C‐O unit in **3 b**, with the magnitude of the interaction between the oxygen‐based lone pair and P−C π* orbital being less than half that of **2 b** (**2 b**: 354.7 kJ mol^−1^; **3 b**: 122.2 kJ mol^−1^). The new C1−O1−Si2 fragment is significantly deviated from the expected bent geometry, with an angle of 160.10(9)°.

A stoichiometric reaction between **3 a** and KO^*t*^Bu at 60 °C for 18 h gave clean conversion to corresponding potassium enolate **4**, which exhibits a ^31^P{^1^H} NMR resonance 104.7 ppm (Scheme 2). Retention of the mesityl group is evident in the ^1^H NMR spectrum of **4**, with singlet signals at 6.71, 2.45 and 2.13 ppm, in a ratio of 2 : 6 : 3. The expected doublet and septet resonances corresponding to the ^*i*^Pr groups coalesce into one singlet resonance, which could not be deconvoluted by ^1^H‐^1^H COSY experiments. No reactivity was not observed between silylated **3 a** and NaO^*t*^Bu or LiO^*t*^Bu.

**Figure 4:**
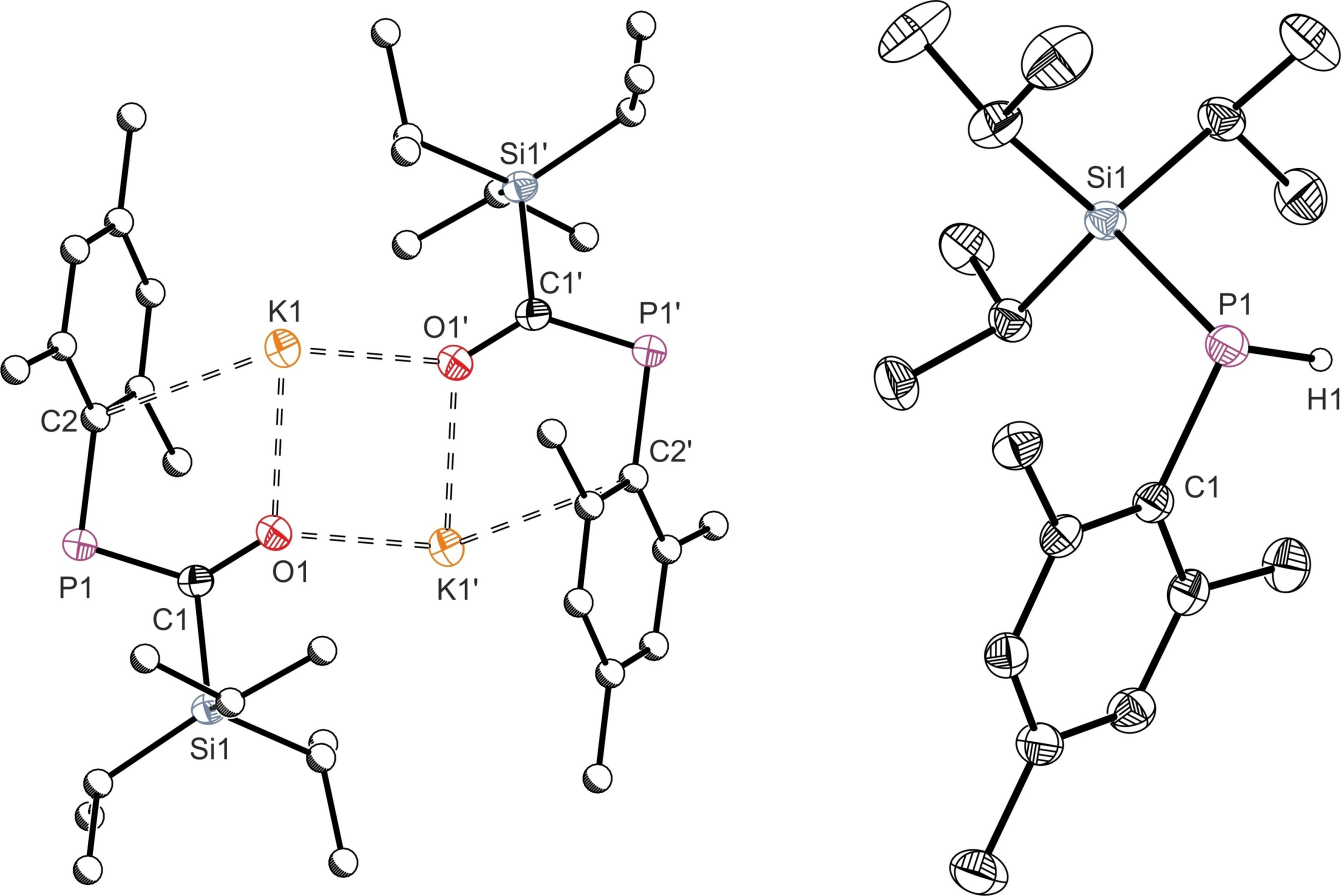
Single crystal X‐ray structures of **4** (left) and **6** (right). Hydrogen atoms are omitted and atoms of Mes* substituents (for **4**) are pictured as spheres of arbitrary radius. Ellipsoids are shown at 50 % probability.

The solid‐state structure of **4** (Figure 4) confirms an analogous structure to lithium enolate **1 a**. Alkyl potassium phospha‐enolates are relatively uncommon, one such species has been previously synthesized from exposure of [K(18‐crown‐6)]P^*t*^Bu_2_ to a CO atmosphere, and its solid state structure indicates a charge‐separated salt with a shorter C−O (1.252(7) Å) and longer C−P (1.786(6) Å) bond when compared with **4** (C−O 1.291(2) Å; C−P 1.739(2) Å).^[15]^ **4** is an unusual dimeric potassium enolate free of external base coordination. Instead, electronic stabilisation is provided by the flanking aryl groups, with relatively short K⋅⋅⋅C contacts in the solid‐state structure indicating weak association (K1⋅⋅⋅C2 3.056(2) Å, K1⋅⋅⋅C3 3.218(2) Å, K1⋅⋅⋅C6 3.290(2) Å and K1⋅⋅⋅C7 3.109(2) Å). When compared with its lithium analogue, **1 a**, the P=C bond in **4** has slightly elongated and the C−O bond has slightly shortened, indicating a greater delocalisation across the P−C−O unit. This is further supported by NBO analysis on the DFT optimised structure of **4**, which indicates significant interactions between the oxygen‐based lone pair and the P−C π* orbital (323.5 kJ mol^−1^).

Reaction of bulkier silyated enolate **3 b** with KO^*t*^Bu did not lead to the isolation of a product analogous with **4**. Several equivalents of KO^*t*^Bu were required to consume **3 b**, and from these mixtures a colourless crystalline solid was isolated. This product is insoluble in most common laboratory solvents and thus its full characterisation has thus far eluded us.

**Scheme 3:**
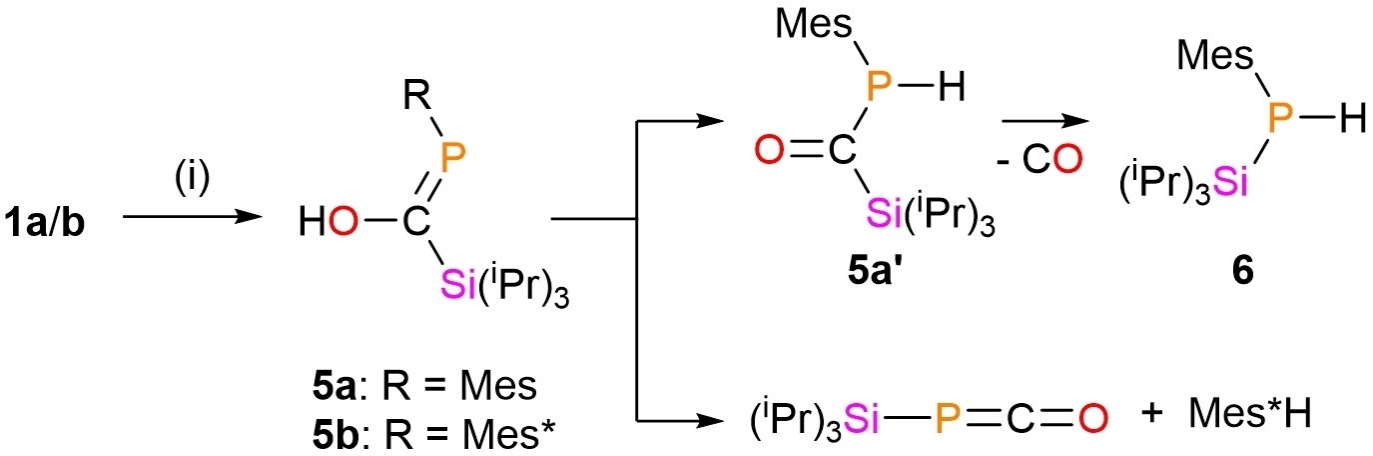
Formation of **5 a** and **5 b** and their contrasting decomposition pathways. (i) stoichiometric H_2_O, toluene.

Addition of degassed water to base‐free enolates **1 a** and **1 b** results in protonation of the enolate with elimination of LiOH, forming **5 a** (^31^P{^1^H} 151.3 ppm) and **5 b** (^31^P{^1^H} 169.1 ppm), respectively (Scheme 3). In the case of mesityl‐substituted **5 a**, whilst initial protonation forms a phospha‐enol, this tautomerizes through a 1,3 H‐shift to the corresponding acyl phosphine isomer **5 a′** within a few hours in solution. This transformation can be monitored by *in situ* ^1^H and ^31^P NMR spectroscopy (Figures S32 and S33), with the appearance of a proton‐coupled ^31^P NMR signal at −20.0 ppm (d, ^1^ *J*_P‐H_=217 Hz). A similar rearrangement was seen for the analogous protonated form of **D**, although the rearrangement in that case was found to proceed slowly in benzene but rapidly in pyridine.^[9]^ When attempting to isolate **5 a**, this rapid rearrangement was also observed in neat samples, making it very challenging to obtain pure samples of **5 a**. Density functional theory (DFT) calculations indicate that acyl phosphine **5 a′** is 10.6 kJ mol^−1^ lower in energy than phospha‐enol **5 a**, providing an energetic driving force for the rapid tautomerization. On further standing at room temperature, mixtures of **5 a** and **5 a′** formed trace amounts of a third product, resonating at −175.9 ppm (d, ^1^ *J*_P‐H_=207 Hz) in the ^31^P NMR spectrum, and heating this mixture results in full conversion to this third compound (see below). No 1,3‐tautomerization was seen in the case of Mes*, and **5 b** was isolated as a colorless solid. Whilst not observed, the corresponding **5 b′** tautomer is calculated to be 7.3 kJ mol^−1^ lower in energy than **5 b**, and we attribute the stability to rearrangement to the larger Mes* preventing the tautomerization through steric interactions.

Heating a toluene or hexane solution of mesityl‐substituted enolate **1 a**, or its silylated analogue **3 a**, for several days results in the formation of a small amount of a new phosphine product, **6**, observed at −175.9 ppm (d, ^1^ *J*_P‐H_=214 Hz) in the ^31^P{^1^H} NMR spectrum. Higher conversions to **6** were achieved using lithium enolate **1 a**, where the maximum conversion which could be achieved was reproducibly around 33 % in hexane, plateauing after 7 days. A new mesityl environment is evident in the ^1^H NMR spectrum of the product mixtures, with singlet resonances at 6.76 (**1 a**: 6.77; **3 a**: 6.82), 2.46 (**1 a**: 2.40; **3 a**: 2.55) and 2.10 (**1 a**: 2.08; **3 a**: 2.18), corresponding to the aryl and *ortho*/*para* methyl groups, respectively. Heating base adduct **2 a** or indeed bulkier analogues **1 b**, **2 b** or **3 b** did not produce any observable analogous reaction. The same product **6** can be generated cleanly by heating the tautomeric mixture of **5 a** and **5 a′**, allowing for further NMR studies. Initially we considered that **6** could be MesPH_2_ from further protonation of **5 a′**, however comparison of chemical shifts allowed us to discount this decomposition pathway (MesPH_2_: ^31^P{^1^H} −156 ppm, ^1^ *J*_P‐H_ 206 Hz).^[16]^ Long range ^1^H‐^13^C HMBC interactions between P−H and the methine C−H, as well as the *ortho*‐CH_3_ of the mesityl group indicated all the functional groups remain on a single molecule, and so we considered the generation of MesP(H)(Si^*i*^Pr_3_) through the extrusion of CO from **5 a′** (Scheme 3). Whilst no evidence for carbon monoxide formation was found by *in situ* ^13^C{^1^H} NMR experiments, crystals of **6** grown at low temperature suitable for X‐ray diffraction revealed that the identity of **6** is indeed this secondary phosphine (Figure 4).

Whilst **6** has been reported as a reaction intermediate,^[17]^ to the best of our knowledge it has not yet been structurally authenticated or isolated. The unexpected formation of this compound through carbon monoxide extrusion from an acyl‐phosphine to our knowledge only has one literature precedent: on warming from −90 °C to room temperature, ^*t*^BuP(H)C(O)(Cl) extrudes CO to form ^*t*^BuP(H)(Cl).^[18]^ Bulkier hydrolysis product **5 b** does not undergo the same 1,3 rearrangement to a phosphine tautomer as **5 a** at room temperature. Under the same thermal conditions for the formation of **6**, **5 b** instead decomposes to Mes*H and ^*i*^Pr_3_SiPCO, presumably through a cyclic 5‐membered transition state (C−P−C−O‐H). In the absence of base, ^*i*^Pr_3_SiPCO is stable and does not undergo any dimerization or cyclisation.^[14]^ In the DFT optimized structure of **5 b** (Figure S43) the interatomic distance between the *ipso*‐carbon atom and hydroxyl proton is reasonably short at 2.232 Å, and with a C−P=C angle of 99.4°, the −OH is geometrically poised for deprotonation. This stark difference in thermal decomposition pathway gives further support for the formation of **6** being solely from tautomer **5 a′**, and not **5 a**.

Given the relatively high, reproducible conversion rates, and that reactivity on heating is limited only to two analogous compounds, it seems unlikely that the formation of **6** from **1 a** or **3 a** is from protonation via trace amounts of water present in the dried and degassed solvents. Further, this reactivity could be observed across different batches of reagents. We hypothesize that **1 a** and **3 a** deprotonate the glassware surface, and that this is the origin of **6** when heating these extremely reactive compounds.

## Conclusion

We have shown that the addition of LiMes or LiMes* across the C≡P bond in ^*i*^Pr_3_SiOCP triggers migration of the silyl group from the oxygen to the carbon atom, resulting in the isolation of lithium phospha‐enolates **1 a** and **1 b**. The dimeric structures of these enolates can be broken up into their monomeric components by addition of 12‐crown‐4 (**2 a** and **2 b**). By utilizing a salt metathesis pathway, silylated silyl enol ethers (**3 a** and **3 b**) can be prepared, from which the corresponding potassiated metal exchange product **4** was synthesized. Hydrolysis of the enolate by addition of degassed water produces phospha‐enols **5 a** and **5 b**, and through an intra‐molecular rearrangement the corresponding acyl‐phosphine forms over the course of hours for smaller Mes‐substituted example **5 a**. Heating **5 a**/**5 a′** cleanly extrudes CO from the acyl‐phosphine, forming silyl phosphine.

## Experimental Section

**General synthetic and analytical procedures**: All reactions and product manipulations were performed under an inert atmosphere of argon or dinitrogen by using standard Schlenk‐line or glovebox techniques (MBraun UNIlab glovebox maintained at <0.1 ppm of H_2_O and <0.1 ppm of O_2_). A procedure for the *in situ* generation of ^*i*^Pr_3_SiOCP from our previous work was used.^[8]^ [Na(dioxane)_3.8_][PCO],^[19]^ 1,3,5‐trimethyl phenyl lithium (MesLi),^[20]^ and 1,3,5‐tri‐tertbutyl phenyl lithium (Mes*Li) were synthesized according to reported synthetic procedures.^[21]^ Triisopropylsilyl trifluoro‐methanesulfonate (Sigma‐Aldrich), 12‐crown‐4 (Sigma‐Aldrich), chlorotrimethylsilane (Sigma‐Aldrich) and potassium tert‐butoxide (Sigma‐Aldrich) were used as received. Distilled water was degassed with argon prior to use. Hexane (Sigma‐Aldrich, HPLC grade), pentane (Sigma‐Aldrich, HPLC grade) and toluene (Sigma‐Aldrich, HPLC grade) were purified by using an MBraun SPS‐800 solvent system. C_6_D_6_ (Aldrich, 99.5 %) was dried over CaH_2_ and degassed prior to use. All dry solvents were stored under argon in gastight ampoules over activated 3 Å molecular sieves.

NMR spectra were acquired on a Bruker Avance Neo 600 MHz NMR spectrometer with a broadband helium cryoprobe (^13^C 151 MHz) or a Bruker AVIII 400 MHz NMR spectrometer (^1^H 400 MHz, ^1^H‐^29^Si HMBC 80 MHz, ^7^Li 156 MHz, ^31^P 162 MHz) at 295 K unless otherwise stated. ^1^H and ^13^C{^1^H} NMR spectra were referenced to residual protic solvent resonance (^1^H NMR C_6_D_6_: δ=7.16 ppm; ^13^C NMR C_6_D_6_: δ=188.06 ppm). ^7^Li NMR were externally referenced to LiCl in D_2_O (1 M). ^29^Si NMR were externally referenced to Me_3_SiH. ^31^P NMR were externally referenced to an 85 % solution of H_3_PO_4_ using the spectrometer reference values. For ^1^H‐^29^Si HMBC experiments a Si−H coupling constant of 6 Hz and a relaxation delay of 1.5 s was used to generate the 2D spectra. Elemental analyses were performed by London Metropolitan University. Samples (∼10 mg) were submitted in vacuum sealed ampoules (solids) or double packaged vials sealed with electrical tape (oils).

**Synthesis of [MesP=C(Si^i^Pr_3_)OLi]_2_ (1 a)**: Triisopropylsilyl triflate (197.1 mg, 0.64 mmol) was dissolved in toluene (5 mL) and [Na(dioxane)_3.8_](PCO) (302 mg, 0.73 mmol, 1.1 eq) was added. The suspension was stirred for 3.5 h and before being filtered. The filtrate containing ^*i*^Pr_3_SiOCP was added to a suspension of mesityl lithium (80.5 mg, 0.64 mmol, 1.0 eq) in toluene (1 mL) and stirred at room temperature overnight. The volatiles were removed to an orange residue which was washed with hexane (3 mL) and dried to an off‐white powder. Concentration of the hexane filtrate yielded a further crop of the title product (Combined crops: 111.5 mg, 0.16 mmol, 50 %). Crystals suitable for single crystal X‐ray diffraction were grown from a standing saturated hexane solution over several days. ^1^H NMR (400 MHz, C_6_D_6_): δ(ppm) 6.77 (s, 2H, Ar*H*), 2.40 (s, 6H, *ortho*‐C*H*_3_), 2.08 (s, 3H, *para*‐C*H*_3_), 1.20 (d, ^3^ *J*_H‐H_=7.2 Hz, 18H, C(H)(C*H*_3_)_2_), 1.14–1.00 (m, 3H, C(*H*)(CH_3_)_2_). ^13^C NMR (151 MHz, C_6_D_6_): δ(ppm) 141.87 (d, ^1^ *J*_C‐P_=4.2 Hz, P=*C*(Si)O), 138.70 (*para*‐Ar*C*), 137.55 (d, ^1^ *J*_C‐P_=58.9 Hz, *ipso*‐Ar*C*) 129.66 (*meta*‐Ar*C*), 22.22 (d, ^3^ *J*_C‐P_=6.7 Hz, *ortho*‐*C*H_3_), 20.86 (*para*‐*C*H_3_), 18.89 (C(H)(*C*H_3_)_2_), 11.60 (d, ^3^ *J*_C‐P_=5.5 Hz, (*C*(H)(CH_3_)_2_)). ^7^Li NMR (156 MHz, C_6_D_6_) δ(ppm) 0.58 (s). ^31^P NMR (162 MHz, C_6_D_6_): δ(ppm) 132.0 (s, ^2^ *J*_P‐Si_=51.5 Hz satellites). ^29^Si NMR (HMBC, 80 MHz, C_6_D_6_): δ(ppm) 1.20 (d, ^2^ *J*_Si‐P_=51.5 Hz). Anal. Calcd (%) for C_38_H_64_Li_2_O_2_P_2_Si_2_: C, 66.64; H, 9.42. Found: C, 66.67; H 9.38.

Synthesis of [Mes*P=C(Si^i^Pr_3_)OLi]_2_ (**1 b**). Triisopropylsilyl triflate (106.4 mg, 0.34 mmol) was dissolved in toluene (5 mL) and [Na(dioxane)_3.8_](PCO) (151.9 mg, 0.37 mmol, 1.1 eq) was added. The suspension was stirred for 3.5 h and before being filtered. The filtrate containing ^*i*^Pr_3_SiOCP was added to a suspension of super‐mesityl lithium (86.1 mg, 0.34 mmol, 1.0 eq) in toluene (1 mL) and stirred at room temperature overnight. The volatiles were removed and the residue recrystallised from a minimum amount of hot toluene (1 mL), and the resulting colourless crystals were filtered and dried (80.9 mg, 0.086 mmol, 51 %). Crystals suitable for single crystal X‐ray diffraction were grown by recrystallisation from a hot C_6_D_6_ solution. ^1^H NMR (400 MHz, C_6_D_6_): δ(ppm) 7.69 (s, 2H, Ar*H*), 1.71 (s, 18H, *ortho*‐C(C*H*_3_)_3_), 1.33 (s, 9H, *para*‐ C(C*H*_3_)_3_), 1.20 (br. s, 18H, C(H)(C*H*_3_)_2_). Methine protons were not observed due to low solubility in C_6_D_6_. A ^13^C{^1^H} NMR spectrum of [Mes*P=C(Si^*i*^Pr_3_)OLi]_2_ could not be obtained due to low solubility. ^7^Li NMR (156 MHz, C_6_D_6_): δ(ppm) −0.11 (s). ^31^P NMR (162 MHz, C_6_D_6_): δ(ppm) 159.5 (s). ^29^Si NMR (HMBC, 80 MHz, C_6_D_6_): δ(ppm) −1.96 (d, ^2^ *J*_Si‐P_=108.02 Hz). Despite repeated attempts, no satisfactory elemental analysis data could be obtained for this compound.

**Synthesis of [MesP=C(Si^i^Pr_3_)O][Li(12‐crown‐4)] (2 a)**: Triisopropylsilyl triflate (100.3 mg, 0.33 mmol) was dissolved in toluene (3 mL) and [Na(dioxane)_3.8_](PCO (144.7 mg, 0.35 mmol, 1.1 eq) was added. The suspension was stirred for 3.5 h and before being filtered. The filtrate containing ^*i*^Pr_3_SiOCP was added to a suspension of mesityl lithium (39.0 mg, 0.31 mmol, 1.0 eq) in toluene (1 mL) and stirred at room temperature overnight. The volatiles were removed to a dark yellow‐orange powder, which was redissolved in toluene (1 mL). A solution of 12‐crown‐4 (56.2 mg, 0.32 mmol, 1.0 eq) in toluene (1 mL) was added and the solution stirred for 3 h. The volatiles were removed to an orange residue which was washed with hexane (3 mL) and dried to an orange powder (98.7 mg, 0.19 mmol, 61 %). ^1^H NMR (400 MHz, C_6_D_6_): δ(ppm) 6.84 (s, 2H, Ar*H*), 3.06 (broad s, 10 H, 12‐crown‐4 C*H*_2_), 2.83 (s, 6H, *ortho*‐C*H*_3_), 2.59 (broad s, 6H, 12‐crown‐4 C*H*_2_), 2.23 (s, 3H, *para*‐C*H*_3_), 1.68 (m, 3H, C(*H*)(CH_3_)_2_), 1.60 (d, ^3^ *J*_H‐H_=6.65 Hz, 18H, C(H)(C*H*_3_)_2_). ^13^C NMR (151 MHz, C_6_D_6_): δ(ppm) 227.98 (d, ^1^ *J*_C‐P_=97.5 Hz, P=*C*), 142.06 (d, ^1^ *J*_C‐P_=58.7 Hz, *ipso*‐Ar*C*) 142.56 (d, ^2^ *J*_C‐P_=4.7 Hz, *ortho*‐Ar*C*), 133.04 (*para*‐Ar*C*), 126.96 (*meta*‐Ar*C*), 67.25 (12‐crown‐4 *C*H_2_), 66.78 (12‐crown‐4 *C*H_2_), 23.47 (d, ^3^ *J*_C‐P_=7.0 Hz, *ortho*‐*C*H_3_), 20.91 (*para*‐*C*H_3_), 19.76 (*C*(H)(CH_3_)_2_), 12.25 (d, ^4^ *J*_C‐P_=4.7 Hz, C(H)(*C*H_3_)_2_). ^7^Li NMR (156 MHz, C_6_D_6_): δ(ppm) −0.06 (s). ^31^P NMR (162 MHz, C_6_D_6_): δ(ppm) 108.5 (s). ^29^Si NMR (HMBC, 80 MHz, C_6_D_6_): δ(ppm) −6.58 (s). Anal. Calcd (%) for C_27_H_48_LiO_5_PSi: C, 62.52; H, 9.33. Found: C, 64.30; N, 9.38.

Syntheis of [Mes*P=C(Si^i^Pr_3_)O][Li(12‐crown‐4)] (2 b): Triisopropylsilyl triflate (107.3 mg, 0.35 mmol) was dissolved in toluene (5 mL) and [Na)(dioxane)_3.8_](PCO) (174.2 mg, 0.42 mmol, 1.3 eq) was added. The suspension was stirred for 3.5 h and before being filtered. The filtrate containing ^*i*^Pr_3_SiOCP was added to a suspension of supermesityl lithium (82.0 mg, 0.33 mmol, 1.0 eq) in toluene (1 mL) and stirred at room temperature overnight. This suspension was added to a solution of 12‐crown‐4 (68.0 mg, 0.56 mmol, 1.7 eq) in toluene (1 mL) and the solution stirred for 3 h. The volatiles were removed to an orange residue which was washed with hexane (2×3 mL) and dried to a pale orange powder (109.4 mg, 0.17 mmol, 52 %). Crystals suitable for X‐ray diffraction were grown from a standing saturated benzene solution. ^1^H NMR (400 MHz, C_6_D_6_): δ(ppm) 7.55 (s, 2H, Ar*H*), 3.07 (br. s, 8H, 12‐crown‐4 C*H*_2_), 2.58 (br. s, 8H, 12‐crown‐4 C*H*_2_), 2.01 (s, 18H, *ortho*‐C(C*H*_3_)_3_), 1.77–1.61 (m, 3H, C(*H*)(CH_3_)_2_), 1.57 (d, ^3^ *J*_H‐H_=7.0 Hz, 18H, C(H)(C*H*_3_)_2_), 1.39 (s, 9H, *para*‐C(C*H*_3_)_3_). ^13^C NMR (151 MHz, C_6_D_6_): δ(ppm) 155.56 (*ortho*‐Ar*C*), 145.33 (*para*‐Ar*C*), 143.02 (d, ^1^ *J*_C‐P_=77.4 Hz, *ipso*‐Ar*C*), 120.10 (*meta*‐Ar*C*H), 67.32 (12‐crown‐4 *C*H_2_), 38.47 (*ortho*‐*C*(CH_3_)_3_), 34.65 (*para*‐*C*(CH_3_)_3_), 32.76 (d, ^4^ *J*_C‐P_=7.2 Hz, *ortho*‐C(*C*H_3_)_3_), 31.72 (*para*‐C(*C*H_3_)_3_), 19.58 (C(H)(*C*H_3_)_2_), 12.59 (d, ^3^ *J*_C‐P_=6.2 Hz, *C*(H)(CH_3_)_2_). ^7^Li NMR (156 MHz, C_6_D_6_): δ(ppm) −0.10. ^31^P NMR (162 MHz, C_6_D_6_): δ(ppm) 126.6 (s). ^29^Si NMR (HMBC, 80 MHz, C_6_D_6_): δ(ppm) −5.43 (s). Anal. Calcd (%) for C_36_H_66_LiO_5_PSi: C, 67.15; H, 10.32. Found: C, 66.71; H 10.28.

**Synthesis of MesP=C(Si^i^Pr_3_)OSiMe_3_ (3 a): [**MesP=C(Si^*i*^Pr_3_)OLi]_2_ (25.8 mg, 0.038 mmol) was suspended in toluene (3 mL) and trimethylsilyl chloride (0.15 mL, 1.18 mmol, excess) was added at room temperature. This was stirred for 18 h. The volatiles were removed and the residue extracted with hexane (3 mL), filtered and dried to a pale‐yellow viscous oil (18.5 mg, 0.045 mmol, 60 %). Pure MesP=C(Si^*i*^Pr_3_)OSiMe_3_ was challenging to manipulate, so for subsequent reactivity studies this compound was synthesized in situ. ^1^H NMR (400 MHz, C_6_D_6_): δ(ppm) 6.73 (s, 2H, Ar*H*), 2.46 (s, 6H, *ortho*‐C*H*_3_), 2.09 (s, 3H, *para*‐C*H*_3_), 1.43 (m, 3H, C(*H*)CH_3_)_3_), 1.30 (d, 18H, ^3^ *J*_H‐H_=7.1 Hz, C(H)(C*H*_3_)_2_), −0.09 (s, ^2^ *J*_H‐Si_=6.7 Hz, 9H, Si(C*H*_3_)_3_ satellites). ^13^C NMR (151 MHz, C_6_D_6_): δ(ppm) 207.37 (d, ^1^ *J*_C‐P_=93.2 Hz, P=*C*), 141.18 (d, ^2^ *J*_C‐P_=6.4 Hz, *ortho*‐Ar*C*), 138.18 (*para*‐Ar*C*), 135.21 (d, ^1^ *J*_C‐P_=51.4 Hz, *ipso*‐*C*), 128.51 (Ar‐*C*H), 22.25 (d, ^3^ *J*_C‐P_=8.8 Hz, *ortho*‐C(*C*H_3_)), 20.73 (*para*‐*C*(CH_3_), 18.81 (*C*(H)(CH_3_)_2_), 11.73 (d, ^4^ *J*_C‐P_=7.2 Hz, C(H)(*C*H_3_)_2_), 0.44 (^1^ *J*_C‐Si_=59.6 Hz, Si(*C*H_3_)_3_). ^31^P NMR (162 MHz, C_6_D_6_): δ(ppm) 190.5 (s, ^2^ *J*_P‐Si_=36.6 Hz satellites). ^29^Si NMR (HMBC, 80 MHz, C_6_D_6_): δ(ppm) 2.92 (s, *Si* ^*i*^Pr_3_), 16.18 (s, *Si*Me_3_). Anal. Calcd (%) for C_27_H_48_LiO_5_PSi: C, 64.65; H, 10.11. Found: C, 65.23; H 10.35.

Synthesis of Mes*P=C(Si^i^Pr_3_)OSiMe_3_ (3 b): [Mes*P=C(Si^*i*^Pr_3_)OLi]_2_ (43.4 mg, 0.046 mmol) was suspended in toluene (3 mL) and trimethylsilyl chloride (0.15 mL, 1.18 mmol, excess) was added at room temperature. The suspension was stirred at room temperature for 18 h. The volatiles were removed and the residue extracted with hexane and filtered. The filtrate was concentrated slowly, and pale‐yellow crystals of the compound were isolated and dried (43.8 mg, 0.082 mmol, 88 %). ^1^H NMR (400 MHz, C_6_D_6_): δ(ppm) 7.53 (s, 2H, Ar*H*), 1.69 (s, 18H, *ortho*‐C(C*H*_3_)_3_), 1.46 (m, 3H, C(*H*)(CH_3_)_2_), 1.34 (s, 9H, *para*‐C(C*H*_3_)_3_), 1.31 (d, ^3^ *J*_H‐H_=7.2 Hz, 18H, C(H)(C*H*_3_)_2_), −0.15 (s, ^2^ *J*_H‐Si_=6.7 Hz, 9H, Si(C*H*_3_)_3_ satellites). ^13^C NMR (151 MHz, C_6_D_6_): δ(ppm) 195.22 (d, ^1^ *J*_C‐P_=95.1 Hz, P=*C*), 154.28 (d, ^2^ *J*_C‐P_=1.8 Hz, *ortho*‐Ar*C*), 149.31 (*para*‐Ar*C*), 134.48 (d, ^1^ *J*_C‐P_=69.7 Hz, *ipso*‐*C*), 121.80 (*meta*‐Ar*C*H), 37.92 (*ortho*‐*C*(CH_3_)_3_), 34.74 (*para*‐*C*(CH_3_)_3_), 32.66 (d, ^5^ *J*_C‐P_=6.7 Hz, *ortho*‐C(*C*H_3_)_3_), 31.11 (*para*‐C(*C*H_3_)_3_), 18.93 (C(H)(*C*H_3_)_2_), 12.40 (d, ^3^ *J*_C‐P_=8.1 Hz, *C*(H)(CH_3_)_2_), 1.79 (Si(*C*H_3_)_3_). ^31^P NMR (162 MHz, C_6_D_6_): δ(ppm) 192.0 (s, ^2^ *J*_P‐Si_=38.7 Hz satellites). ^29^Si NMR (HMBC, 80 MHz, C_6_D_6_): δ(ppm) 11.34 (s, *Si*Me_3_) and 2.52 (s, *Si* ^*i*^Pr_3_). Anal. Calcd (%) for C_27_H_48_LiO_5_PSi: C, 69.60; H, 11.12. Found: C, 69.80; H 11.21; N.

**Synthesis of [MesP=C(Si^i^Pr_3_)OK]_2_ (4)**: [MesP=C(Si^*i*^Pr_3_)OLi]_2_ (49.2 mg, 0.072 mmol) was dissolved in toluene (3 mL) and Me_3_SiCl (0.15 mL, 1.2 mmol, 16 eq) was added. The solution was stirred for 18 h at room temperature. The volatiles were removed and the residue extracted with hexane (5 mL) and filtered. KO^*t*^Bu (19.4 mg, 0.17 mmol, 2.4 eq) was added to the filtrate, and the mixture heated to 60 °C for 18 h. The volatiles were removed, and the residue washed with hexane (3 mL) and dried to a pale yellow powder. A second crop was obtained by concentration of the hexane washings, filtration and drying (combined crops 25.1 mg, 0.034 mmol, 47 %). Crystals for X‐ray diffraction were grown from a standing saturated hexane solution. ^1^H NMR (400 MHz, C_6_D_6_): δ(ppm) 6.71 (s, 2H, Ar*H*), 2.45 (s, 6H, *ortho*‐C*H*_3_), 2.13 (s, 3H, *para*‐C*H*_3_), 1.32 (s, 21H, C(*H*)(C*H*_3_)_2_, two resonances overlap). ^13^C NMR (151 MHz, C_6_D_6_): δ(ppm) 228.60 (d, ^1^ *J*_C‐P_=99.31 Hz, P=*C*), 141.81 (*ortho*‐Ar*C*), 141.52 (d, ^1^ *J*_C‐P_=81.90 Hz, *ipso*‐Ar*C*), 135.42 (*para*‐Ar*C*), 127.91 (*meta*‐Ar*C*H, obscured by solvent peak), 22.95 (d, ^3^ *J*_C‐P_=7.4 Hz, *ortho*‐*C*H_3_), 20.63 (*para*‐*C*H_3_), 19.48 (*C*(H)(CH_3_)_2_), 12.05 (d, ^4^ *J*_C‐P_=5.2 Hz, C(H)(*C*H_3_)_2_). ^31^P NMR (162 MHz, C_6_D_6_): δ(ppm) 102.7 (s). ^29^Si NMR (HMBC, 80 MHz, C_6_D_6_): δ(ppm) −5.05 (d, ^2^ *J*_Si‐P_=117.79 Hz). Anal. Calcd (%) for C_38_H_64_K_2_O_2_P_2_Si_2_: C, 60.92; H, 8.61. Found: C, 60.59; H 8.40.

**Synthesis of a mixture of MesP=C(Si^i^Pr_3_)OH and Mes(H)PC(Si^i^Pr_3_)(O) (5 a and 5 a′)**: *In situ*: [MesP=C(Si^*i*^Pr_3_)OLi]_2_ was suspended in C_6_D_6_ (0.5 mL) and excess degassed water (0.1 mL) was added. The mixture was sonicated for 1 minute to ensure full miscibility of water in the solvent (Figure S20a, Figure S21a). The volatiles were removed under vacuum, with the residue heated to 50 °C to ensure all water was removed. The yellow oily residue was then redissolved in C_6_D_6_ and filtered to remove precipitated LiOH (Figure S20b, Figure S21b). Tautomerization was measured by ^1^H and ^31^P NMR spectroscopy, 20 h (Figure S20c, Figure S21c) and 4 days (Figure S20d, Figure S21d) after the addition of water. MesP=C(Si ^*i*^ Pr_3_ )OH: ^1^H NMR (400 MHz, C_6_D_6_): δ(ppm) 6.70 (s, 2H, Ar*H*), 6.66 (d, ^3^ *J*_P‐H_=4.1 Hz, 1H, O*H*), 2.29 (s, 6H, *ortho*‐C*H*_3_), 2.06 (s, 3H, *para*‐C*H*_3_), 1.51–1.35 (m, 3H, C(*H*)(CH_3_)_2_), 1.28 (d, ^3^ *J*_H‐H_=7.3 Hz, 18H, C(H)(C*H*_3_)_2_). ^31^P NMR (162 MHz, C_6_D_6_): δ(ppm) 151.3 (s). MesP(H)C(Si ^*i*^ Pr_3_ )(O): ^1^H NMR (400 MHz, C_6_D_6_): δ(ppm) 6.76 (s, 2H, Ar*H*), 5.69 (d, ^1^ *J*_P‐H_=234.7 Hz, 1H, P*H*), 2.33 (s, 6H, *ortho*‐C*H*_3_), 2.08 (s, 3H, *para*‐C*H*_3_), 1.35–1.29 (m, overlapping with adjacent signal, C(*H*)(CH_3_)_2_), 1.12 (d, ^3^ *J*_H‐H_=7.4 Hz, 18H, C(H)(C*H*_3_)_2_). ^31^P NMR (162 MHz, C_6_D_6_): δ(ppm) −20.0 (d, ^1^ *J*_P‐H_=217 Hz). Due to instability in solution, no ^13^C{^1^H} NMR spectra were collected. *Preparative scale*: [MesP=C(Si^*i*^Pr_3_)OLi]_2_ (15.5 mg, 0.023 mmol) was suspended in toluene (0.5 mL) and excess degassed water (0.1 mL) was added, which dissolved the suspension creating a bi‐phasic mixture. After 2 h, the volatiles were removed thoroughly and the residue extracted with pentane and filtered. The volatiles were removed from the filtrate to give a bright yellow oil containing a mixture of MesP=C(Si^*i*^Pr_3_)OH and Mes(H)PC(Si^*i*^Pr_3_) (11.5 mg, 0.034 mmol, 75 %). Anal. Calcd (%) for C_19_H_33_OPSi: C, 67.81; H, 9.88. Found: C, 63.33; H, 9.61. Neat MesP(H)C(Si^*i*^Pr_3_)(O) extrudes CO over time, and this is reflected in the elemental analysis values, which are consistent with CHN analysis of MesP(H)(Si^*i*^Pr_3_) (**6**).

Synthesis of Mes*P=C(Si^i^Pr_3_)OH (5 b): [Mes*P=C(Si^*i*^Pr_3_)OLi]_2_ (25.6 mg, 0.027 mmol) was suspended in toluene (0.5 mL) and degassed after which water (0.1 mL, 5.6 mmol, excess) was added. The mixture was sonicated, and after 5 minutes no solid remained in the organic fraction. The volatiles were removed under vacuum, with the residue heated to 50 °C to ensure all water was removed. The residue was extracted with hexane (0.5 mL) and filtered, then concentrated to 0.1 mL by evaporation. Cooling to −35 °C produced pale yellow crystals of Mes*P=C(Si^*i*^Pr_3_)OH, which when isolated and warmed to room temperature melted to an oil (19.9 mg, 0.043 mmol, 78 %). Attempts were made to collect X‐ray diffraction data of crystals kept at low temperature, however samples were repeatedly polycrystalline and not suitable for single crystal X‐ray diffraction. ^1^H NMR (400 MHz, C_6_D_6_): δ(ppm) 6.70 (s, 2H, Ar*H*), 5.18 (d, ^3^ *J*_H‐P_=4.6 Hz, 1H, O*H*), 1.16 (s, 18H, C(C*H*_3_)_3_, *ortho*‐ and *para*‐positions overlap), 1.03–0.91 (m, 3H, C(*H*)(CH_3_)_2_), 0.82 (d, ^3^ *J*_H‐H_=6.8 Hz, 18H, C(H)(C*H*_3_)_2_).^13^C NMR (151 MHz, C_6_D_6_): δ(ppm) 199.59 (d, ^1^ *J*_C‐P_=90.3 Hz ^3^ *J*_C‐Si_=55.7 Hz satellites, *ipso*‐Ar*C*), 156.66 (*ortho*‐Ar*C*), 150.54 (*para*‐Ar*C*), 122.28 (*meta*‐Ar*C*), 38.03 (*ortho*‐C(*C*H_3_)_3_), 34.76 (*para*‐C(C*H*_3_)_3_), 32.40 (d, ^3^ *J*_C‐P_=6.7 Hz, *ortho*‐*C*(CH_3_)_3_), 30.99 (*para*‐*C*(CH_3_)_3_), 18.70 (^2^ *J*_C‐Si_=31.7 Hz, C(H)(*C*H_3_)_2_ satellites), 11.61 (d, ^3^ *J*_C‐P_=6.1 Hz, ^1^ *J*_C‐Si_=55.8 Hz, *C*(H)(CH_3_)_2_ satellites). ^31^P NMR (162 MHz, C_6_D_6_): δ(ppm) 169.1 (s). ^29^Si NMR (HMBC, 80 MHz, C_6_D_6_): δ(ppm) 0.47 (s). Anal. Calcd (%) for C_27_H_48_LiO_5_PSi: C, 72.67; H, 11.11. Found: C, 72.26; H, 10.63.

**Synthesis of MesP(H)(Si^i^Pr_3_) (6)**: [MesP=C(Si^*i*^Pr_3_)OLi]_2_ (25.0 mg, 0.037 mmol) was dissolved in 2 mL toluene and degassed water (0.1 mL, 5.6 mmol, excess) was added. The resulting solution was stirred at 80 °C overnight before the volatiles were removed. The residue was extracted into hexane (1 mL), filtered and dried to a yellow oil. Hexane (2 drops) was added to the residue and the solution cooled to −35 °C to form colourless crystals (8.3 mg, 0.027 mmol, 72 %). These crystals were used for single crystal X‐ray diffraction experiments. ^1^H NMR (400 MHz, C_6_D_6_): δ(ppm) 6.76 (s, 2H, Ar*H*), 3.60 (d, ^1^ *J*_P‐H_=214 Hz, 1H, P*H*), 2.46 (s, 6H, *ortho*‐C*H*_3_), 2.10 (s, 3H, *para*‐C*H*_3_), 1.25–1.14 (m, 3H, C(*H*)(CH_3_)_2_), 1.08 (d, ^3^ *J*_H‐H_=7.2 Hz, 9H, C(H)(C*H*_3_)_2_), 1.02 (d, ^3^ *J*_H‐H_=7.3 Hz, 9H, C(H)(C*H*_3_)_2_). ^13^C NMR (151 MHz, C_6_D_6_): δ(ppm) 141.05 (d, ^2^ *J*_C‐P_=11.26 Hz, *ortho*‐Ar*C*), 135.65 (*para*‐Ar*C*), 128.94 (d, ^3^ *J*_C‐P_=3.46 Hz, *meta*‐ArC*H*), 24.15 (d, %bk;^3^ *J*_C‐P_=12.2 Hz, *ortho*‐*C*H_3_), 20.58 (*para*‐*C*H_3_), 18.83 (d, ^3^ *J*_C‐P_=4.13 Hz, C(H)(*C*H_3_)_2_), 18.71 (d, ^3^ *J*_C‐P_=1.82 Hz, C(H)(*C*H_3_)_2_), 13.80 (d, ^2^ *J*_C‐P_=7.61 Hz, *C*(H)(CH_3_)_2_). P=*C* and *ipso*‐Ar*C* not observed. ^31^P NMR (162 MHz, C_6_D_6_): δ(ppm) −175.9 (d, ^1^ *J*_P‐H_=214 Hz). ^29^Si NMR (HMBC, 80 MHz, C_6_D_6_): δ(ppm) 14.48 (s). Anal. Calcd (%) for C_18_H_33_PSi: C, 70.08; H, 10.78. Found: C, 63.66; H 9.49.

## Conflict of interest

The authors declare no conflict of interest.

1

## Supporting information

As a service to our authors and readers, this journal provides supporting information supplied by the authors. Such materials are peer reviewed and may be re‐organized for online delivery, but are not copy‐edited or typeset. Technical support issues arising from supporting information (other than missing files) should be addressed to the authors.

Supporting Information

## Data Availability

The data that support the findings of this study are available in the supplementary material of this article.
