# Supplementary material for: Formation, Reactivity and Decomposition of Aryl Phospha‐Enolates
Source: Chemistry. 2022 Dec 15;29(8):e202203081. doi: 10.1002/chem.202203081 (PMC10108052; doi:10.1002/chem.202203081)
Supplement: Supplementary file 1 — Supporting Information [file CHEM-29-0-s001.pdf]

Chemistry–A European Journal

Supporting Information

# Formation, Reactivity and Decomposition of Aryl Phospha-Enolates

Stephanie J. Urwin* and Jose M. Goicoechea*

**Contents**

1. NMR spectra ........ 1
2. Crystallographic Data ........ 17
3. DFT Calculations ........ 19
Geometry Optimisations ........ 19
XYZ Coordinates of Optimised Structures ........ 21
4. References ........ 32

# 1. NMR spectra

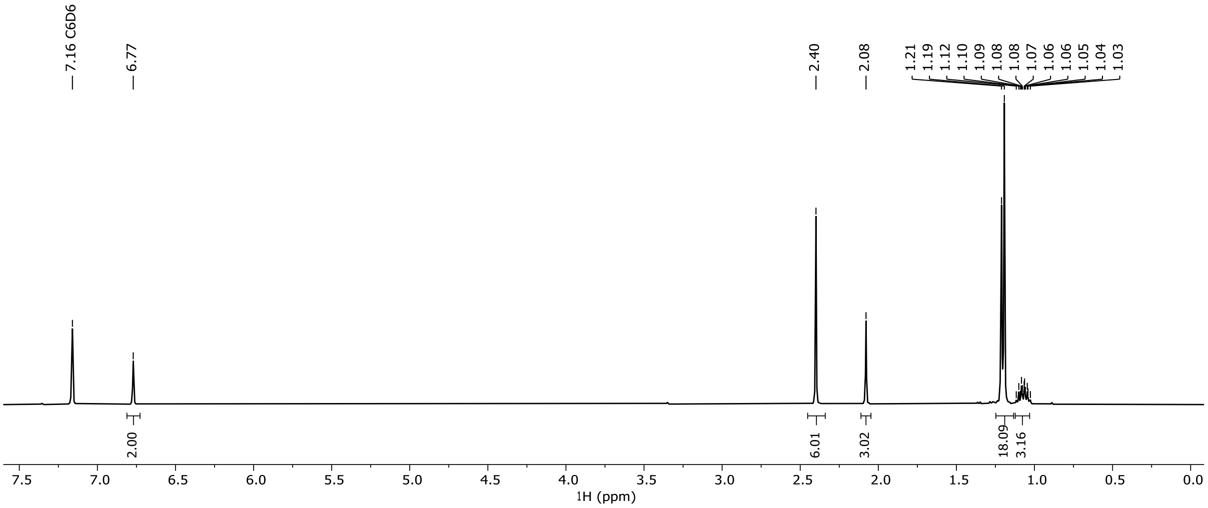


**Figure S1.** $^{1}H$ NMR spectrum (400 MHz, $C_6D_6$) of [MesP=C(Si$^{i}$Pr$_3$)OLi]$_2$ (**1a**).

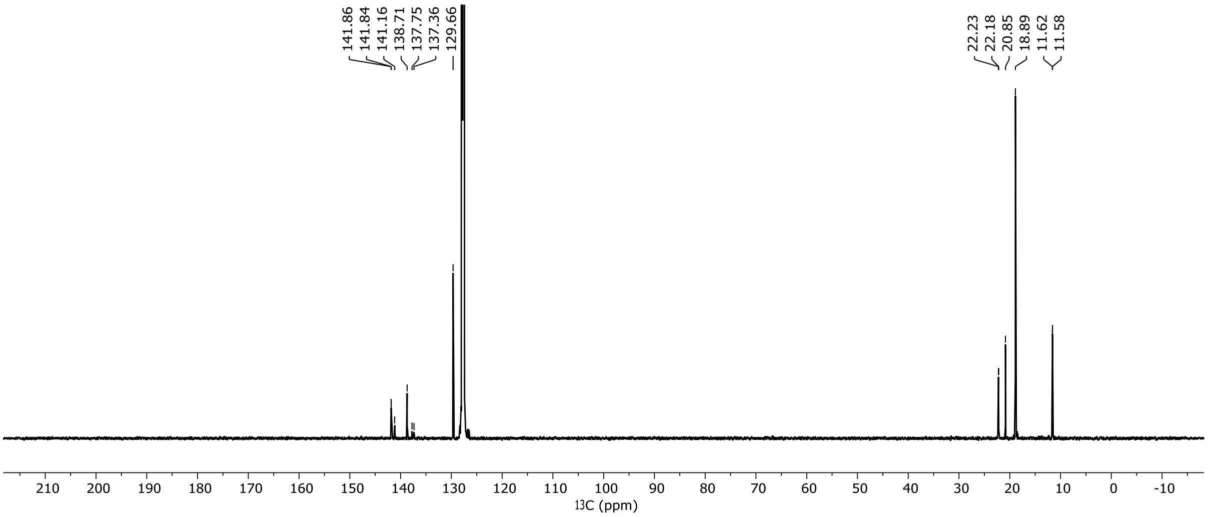


**Figure S2.** $^{13}C$ NMR spectrum (151 MHz, $C_6D_6$) of [MesP=C(Si$^{i}$Pr$_3$)OLi]$_2$ (**1a**).

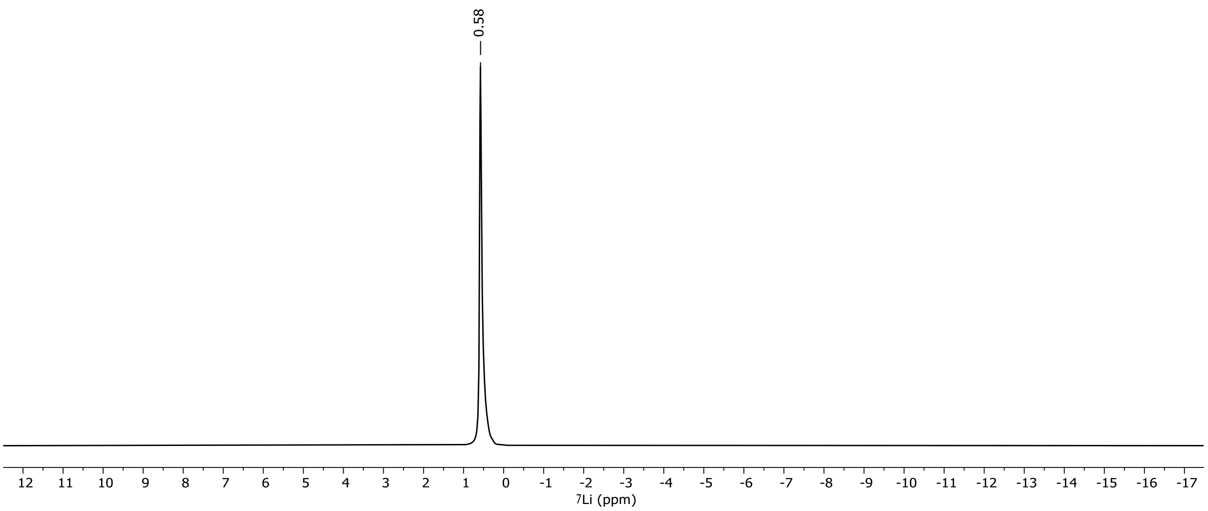


**Figure S3.** $^{7}$Li NMR spectrum (156 MHz, $C_6D_6$) of [MesP=C(Si$^{i}$Pr$_3$)OLi]$_2$ (**1a**).

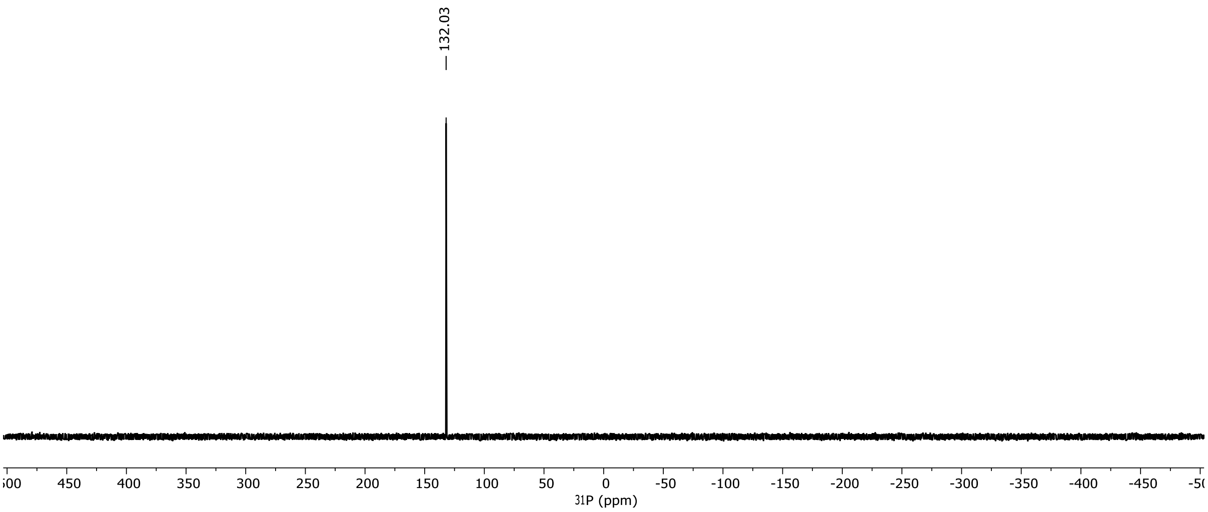


**Figure S4.** $^{31}$P NMR spectrum (162 MHz, $C_6D_6$) of [MesP=C(Si$^{i}$Pr$_3$)OLi]$_2$ (**1a**).

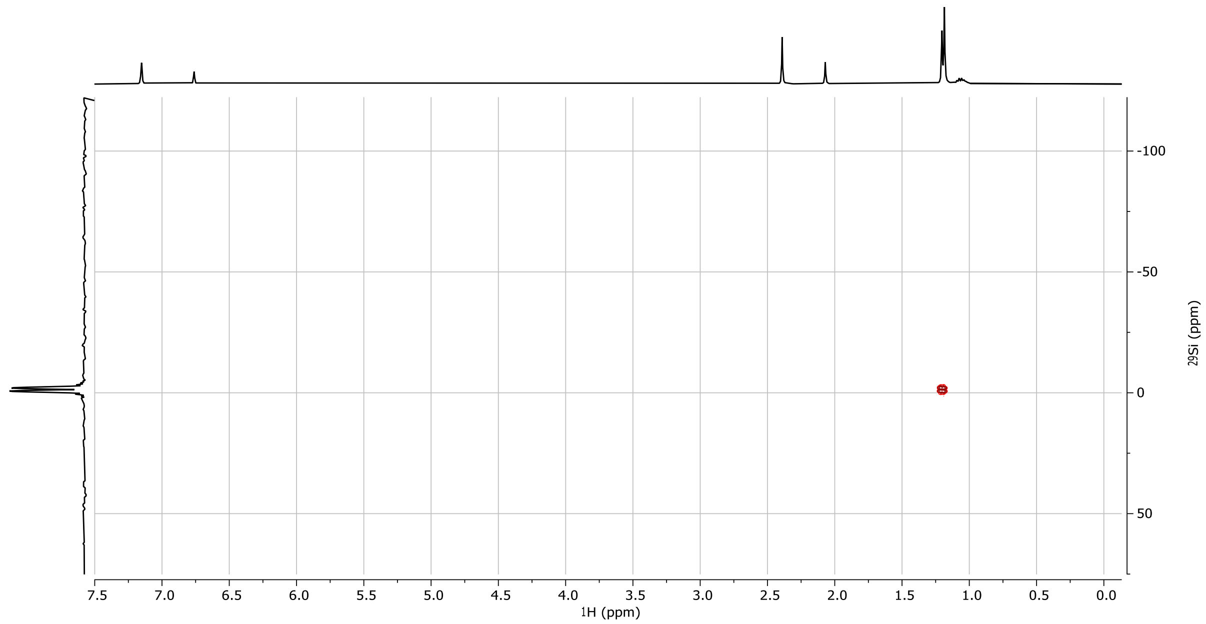


**Figure S5.** $^{1}$H-$^{29}$Si HMBC NMR spectrum (80 MHz, $C_6D_6$) of [MesP=C(Si$^{i}$Pr$_3$)OLi]$_2$ (**1a**).

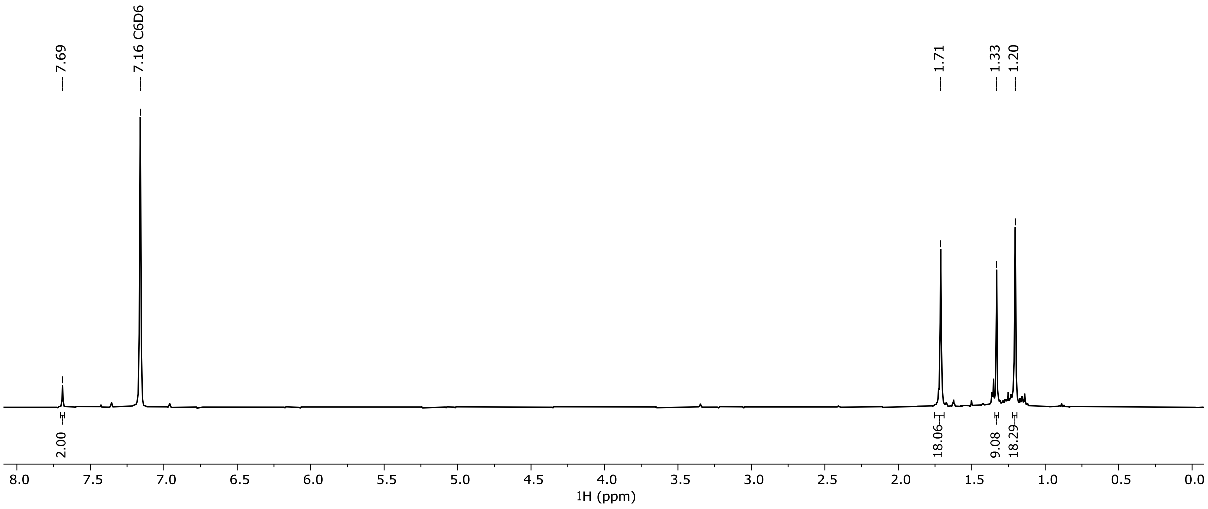


**Figure S6.** $^{1}H$ NMR spectrum (400 MHz, $C_6D_6$) of [Mes*P=C(Si$^{i}$Pr$_3$)OLi]$_2$ (**1b**). **1b** is sparingly soluble in $C_6D_6$.

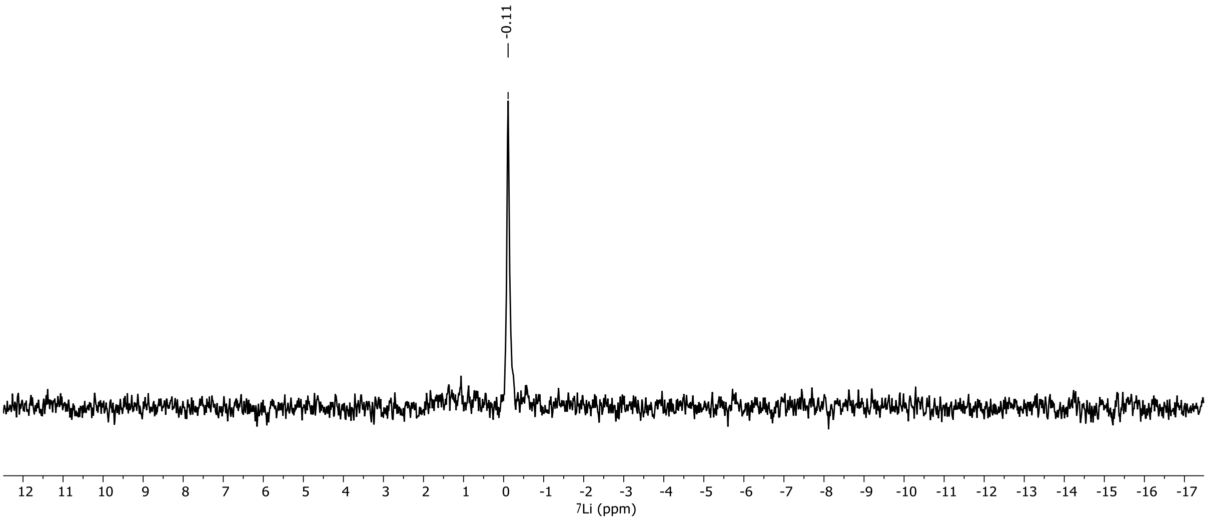


**Figure S7.** $^{7}Li$ NMR spectrum (156 MHz, $C_6D_6$) of [Mes*P=C(Si$^{i}$Pr$_3$)OLi]$_2$ (**1b**).

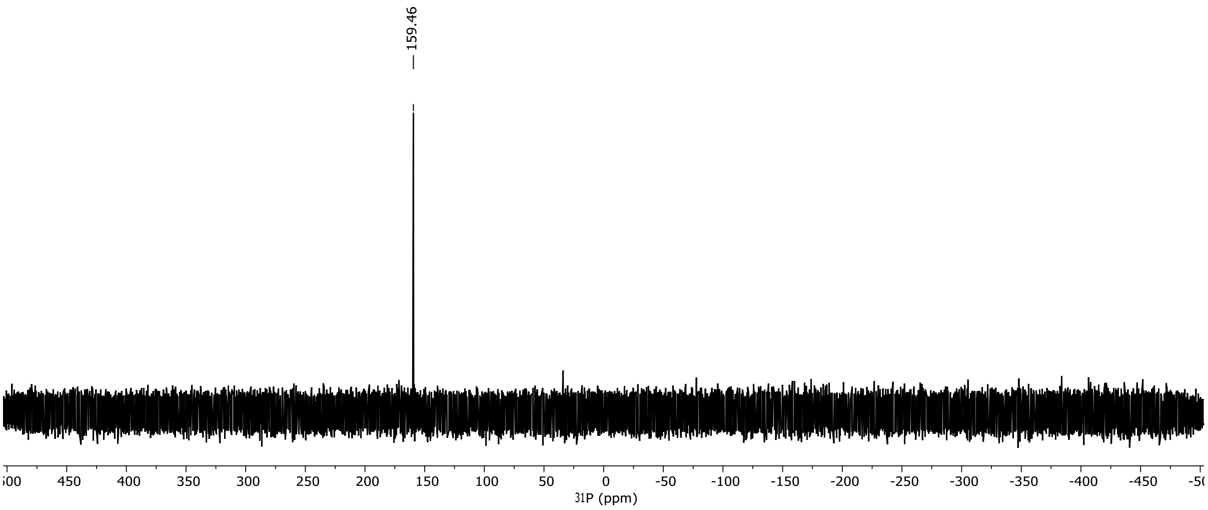


**Figure S8.** $^{31}P$ NMR spectrum (162 MHz, $C_6D_6$) of [Mes*P=C(Si$^{i}$Pr$_3$)OLi]$_2$ (**1b**).

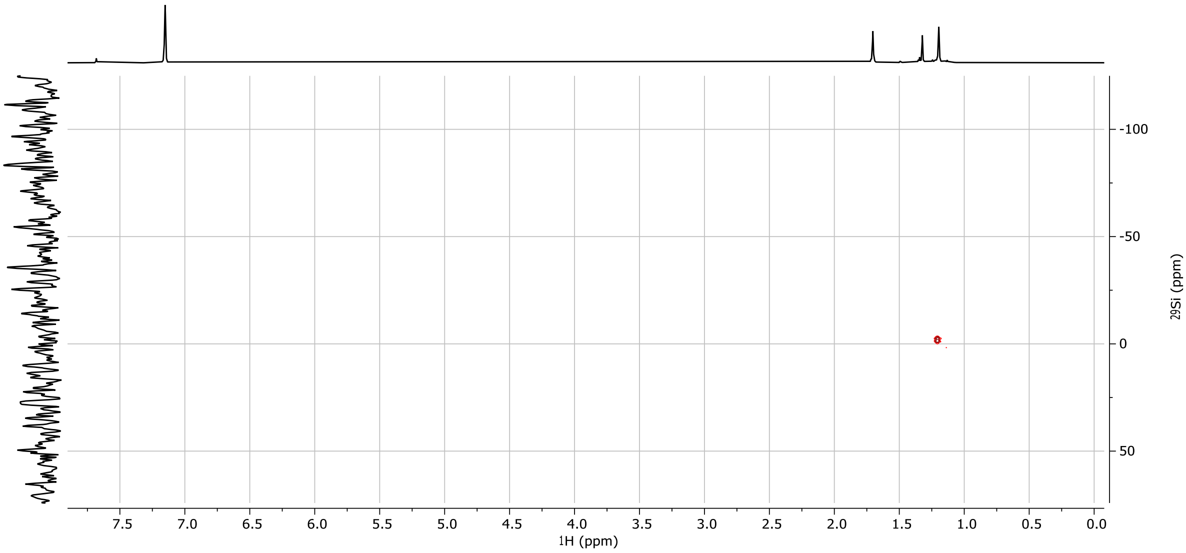


**Figure S9.** $^{1}$H-$^{29}$Si HMBC NMR spectrum (80 MHz, $C_6D_6$) of [Mes*P=C(Si$^{i}$Pr$_3$)OLi]$_2$ (**1b**).

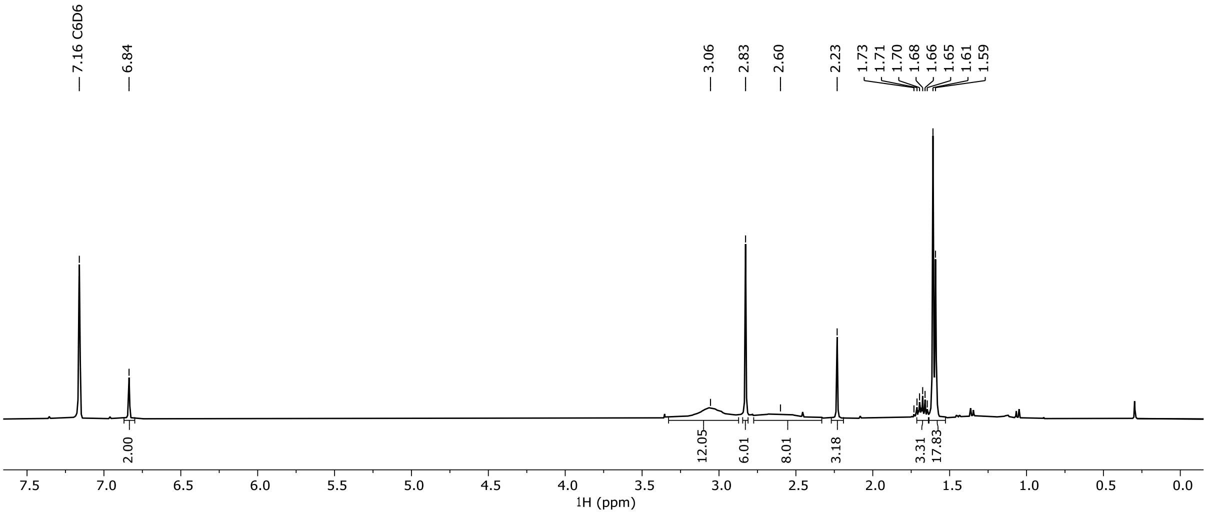


**Figure S10.** $^{1}$H NMR spectrum (400 MHz, $C_6D_6$) of MesP=C(Si$^{i}$Pr$_3$)OLi(12-crown-4) (**2a**).

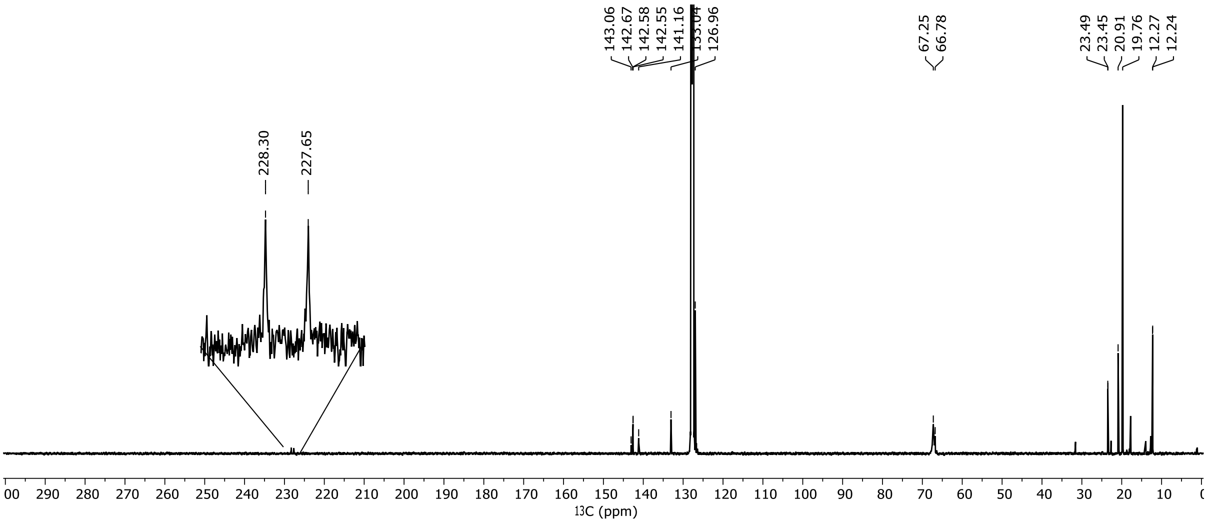


**Figure S11.** $^{13}$C NMR spectrum (151 MHz, $C_6D_6$) of MesP=C(Si$^{i}$Pr$_3$)OLi(12-crown-4) (**2a**).

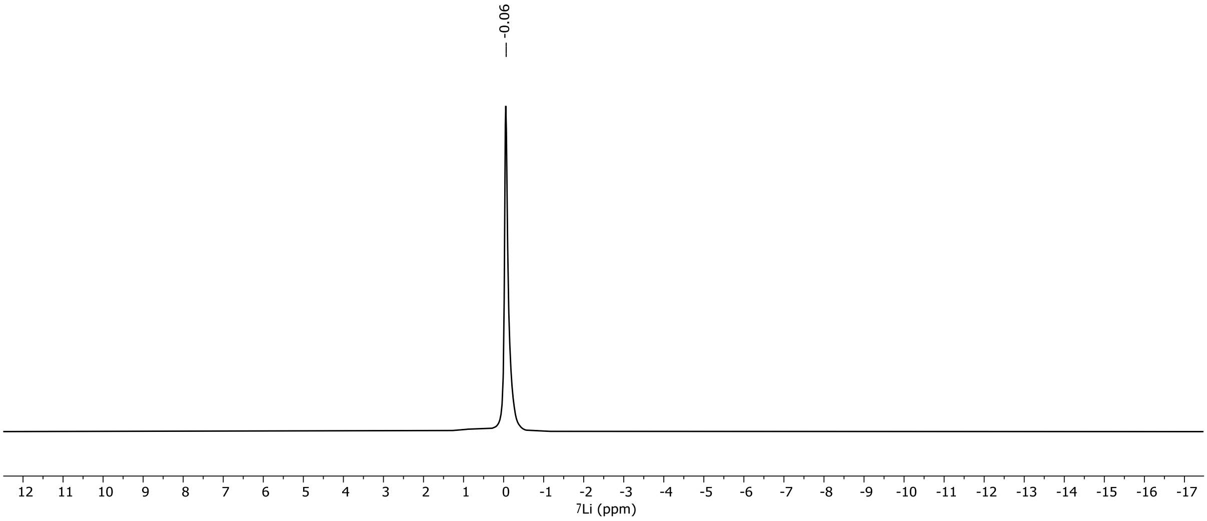


**Figure S12.** $^7$Li NMR spectrum (156 MHz, $C_6D_6$) of MesP=C(Si$^i$Pr$_3$)OLi(12-crown-4) (**2a**).

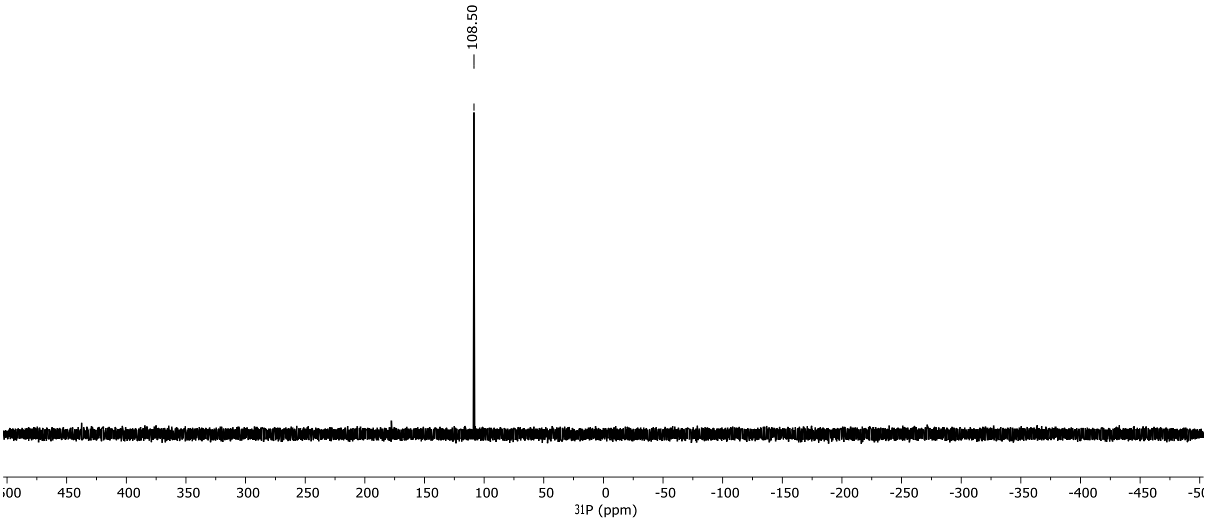


**Figure S13.** $^{31}$P NMR spectrum (162 MHz, $C_6D_6$) of MesP=C(Si$^i$Pr$_3$)OLi(12-crown-4) (**2a**).

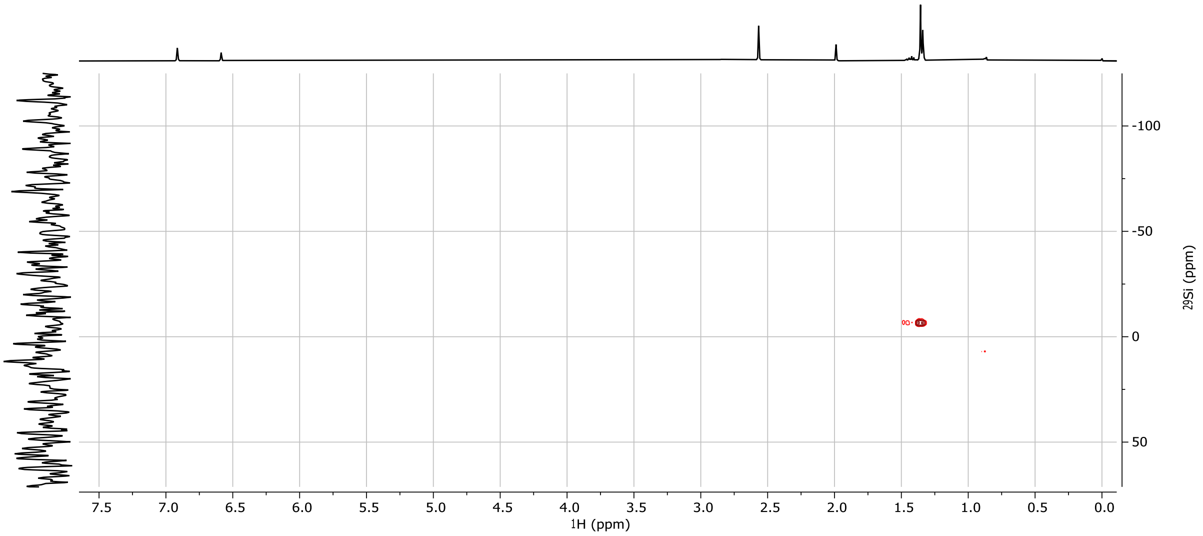


**Figure S14.** H-$^{29}$Si HMBC NMR spectrum (80 MHz, $C_6D_6$) of MesP=C(Si$^i$Pr$_3$)OLi(12-crown-4) (**2a**).

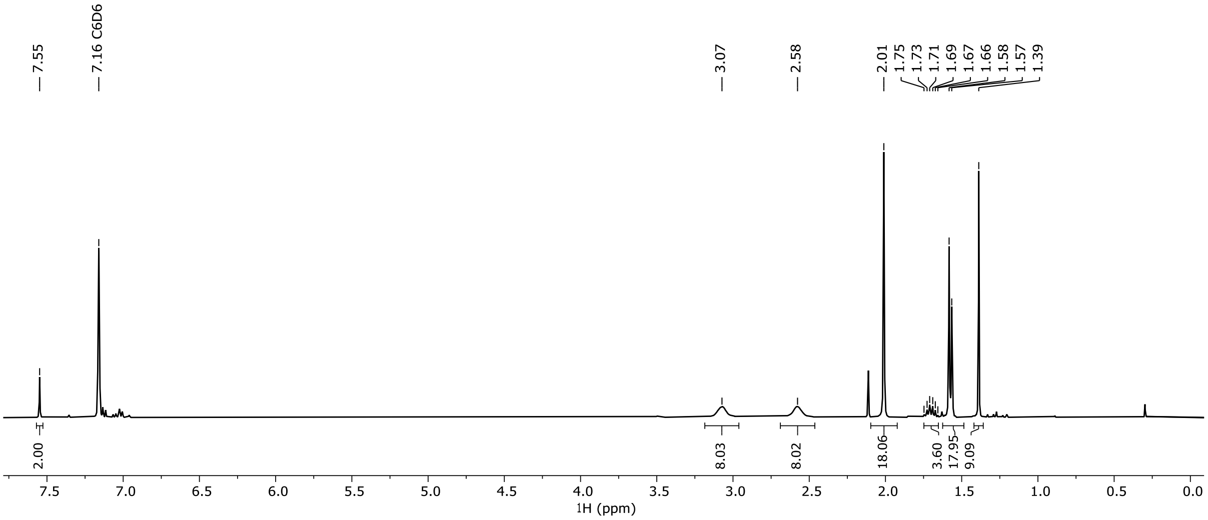


**Figure S15.** $^{1}$H NMR spectrum (400 MHz, $C_6D_6$) of Mes*P=C(Si$^{i}$Pr$_3$)OLi(12-crown-4) (**2b**).

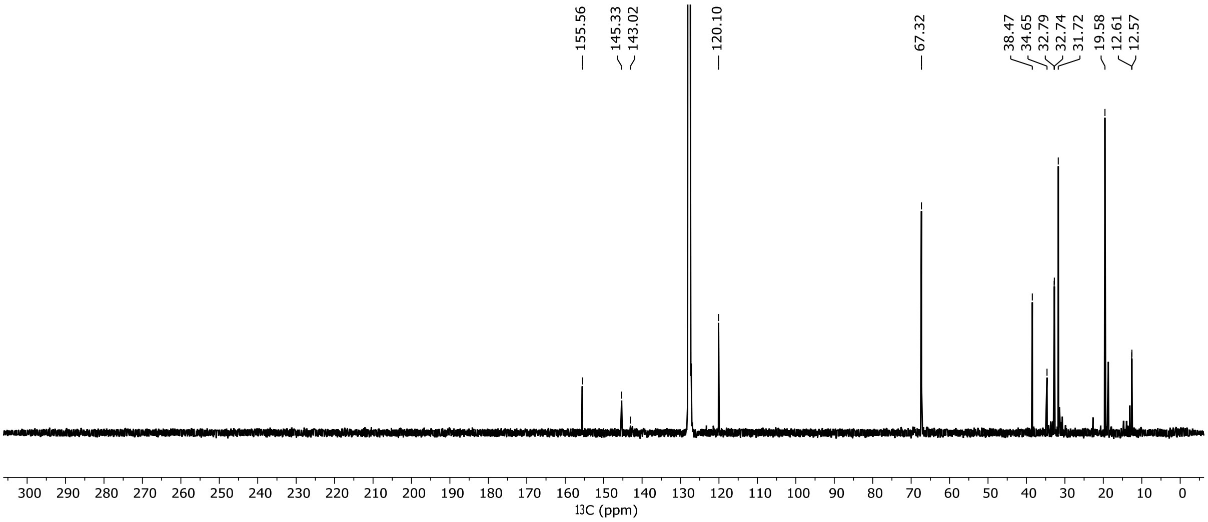


**Figure S16.** $^{13}$C NMR spectrum (151 MHz, $C_6D_6$) of Mes*P=C(Si$^{i}$Pr$_3$)OLi(12-crown-4) (**2b**).

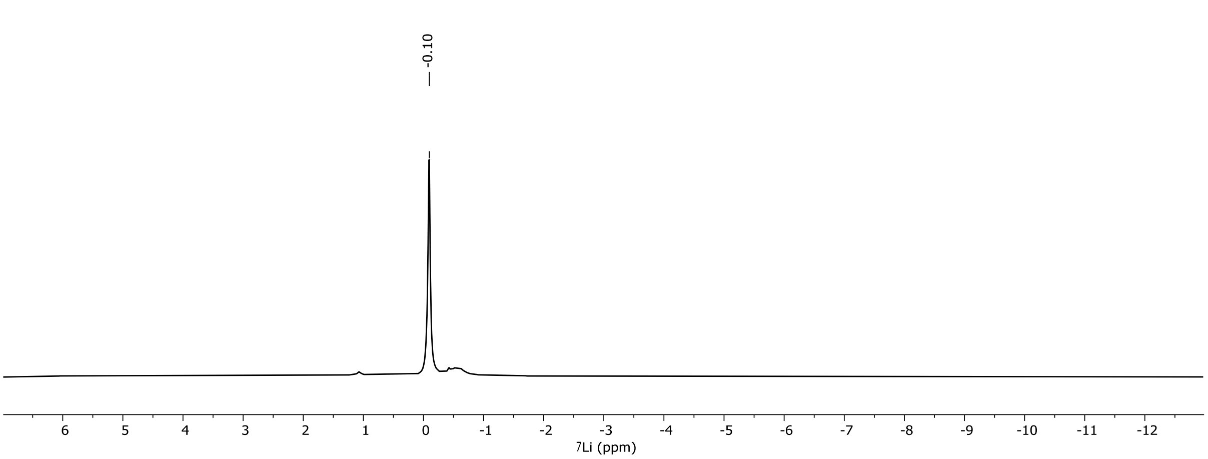


**Figure S17.** $^{7}$Li NMR spectrum (156 MHz, $C_6D_6$) of Mes*P=C(Si$^{i}$Pr$_3$)OLi(12-crown-4) (**2b**).

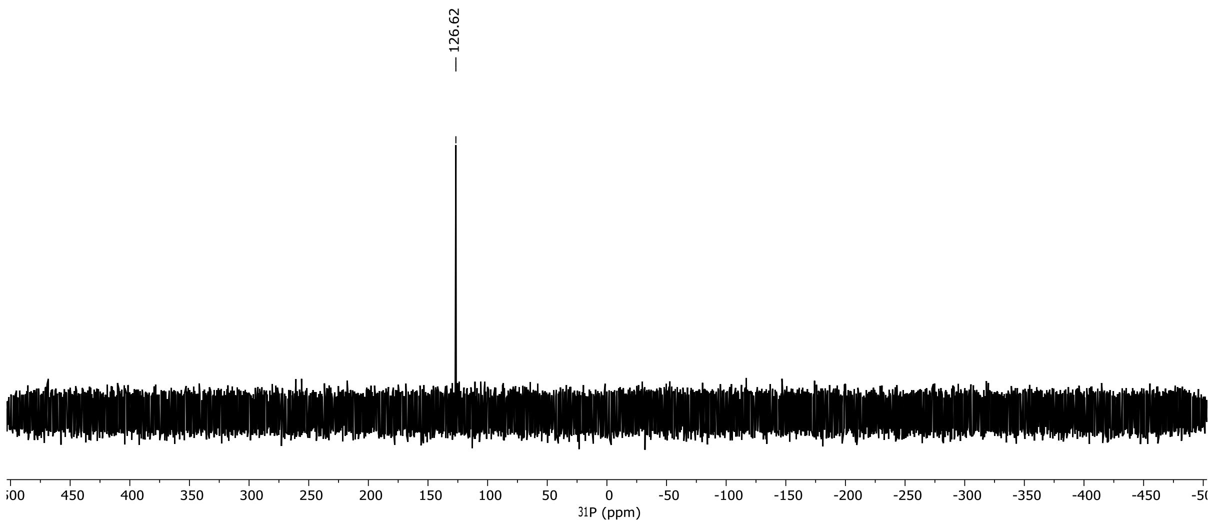


**Figure S18.** $^{31}P$ NMR spectrum (162 MHz, $C_6D_6$) of Mes*P=C($Si^iPr_3$)OLi(12-crown-4) (**2b**).

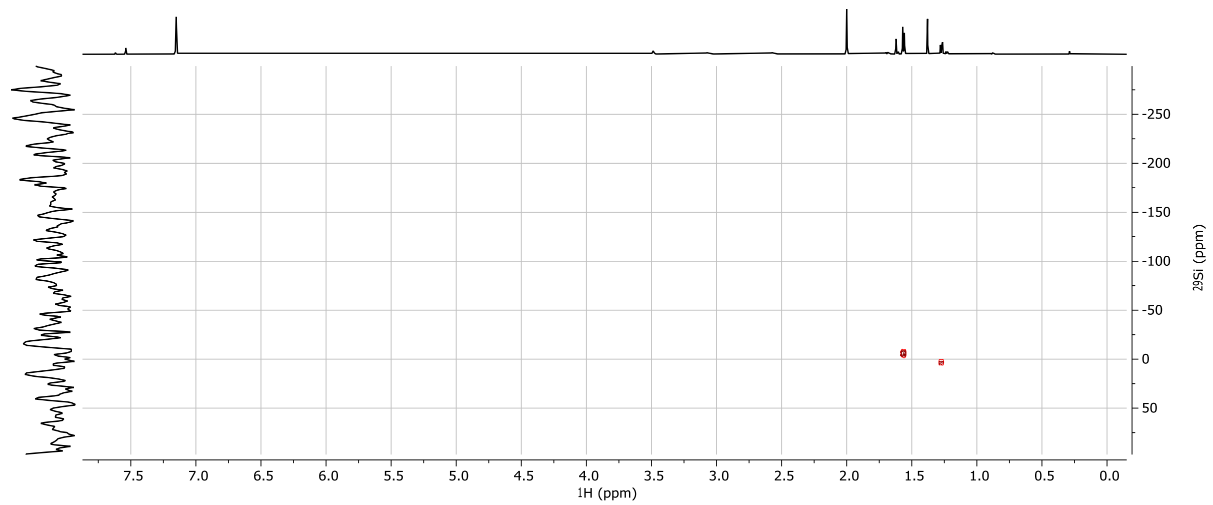

**Figure S19.** H-$^{29}Si$ HMBC NMR spectrum (80 MHz, $C_6D_6$) of Mes*P=C($Si^iPr_3$)OLi(12-crown-4) (**2b**).

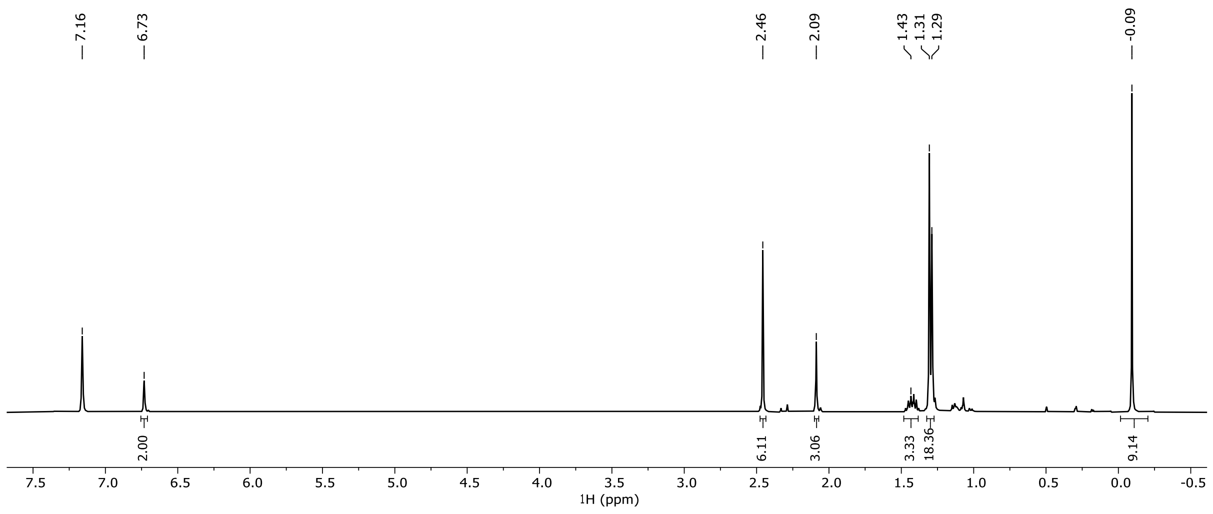

**Figure S20.** $^1H$ NMR spectrum (400 MHz, $C_6D_6$) of MesP=C($Si^iPr_3$)OSiMe$_3$ (**3a**).

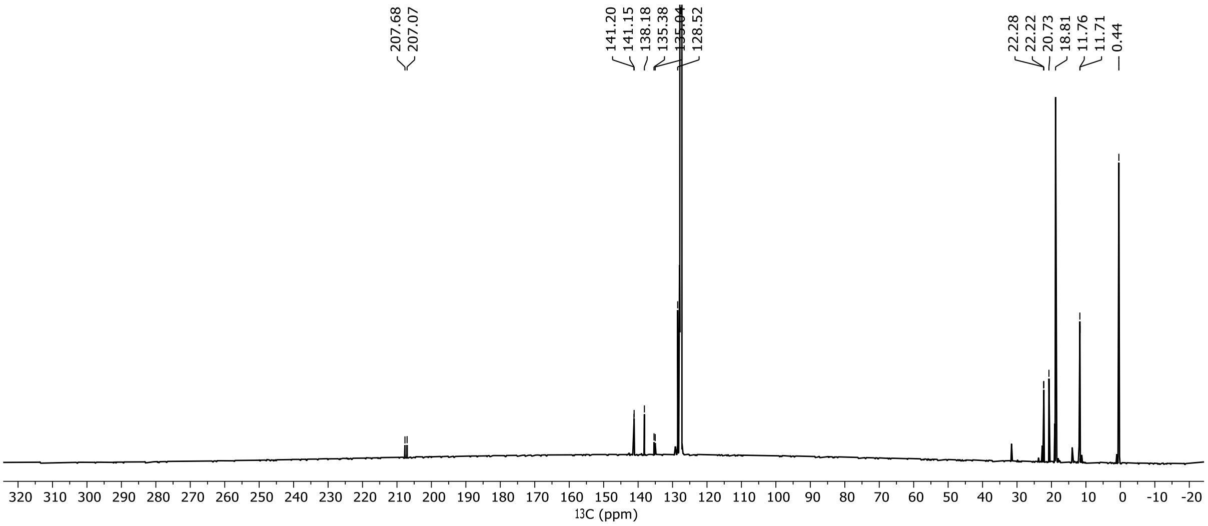


**Figure S21.** $^{13}C$ NMR spectrum (151 MHz, $C_6D_6$) of MesP=C(Si$^i$Pr$_3$)OSiMe$_3$ (**3a**).

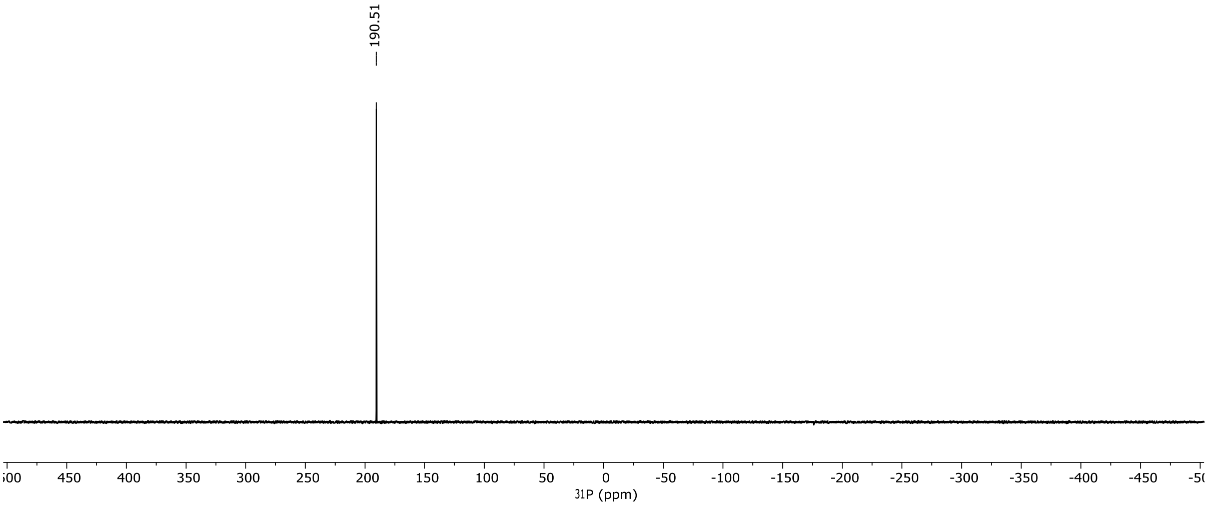


**Figure S22.** $^{31}P$ NMR spectrum (162 MHz, $C_6D_6$) of MesP=C(Si$^i$Pr$_3$)OSiMe$_3$ (**3a**).

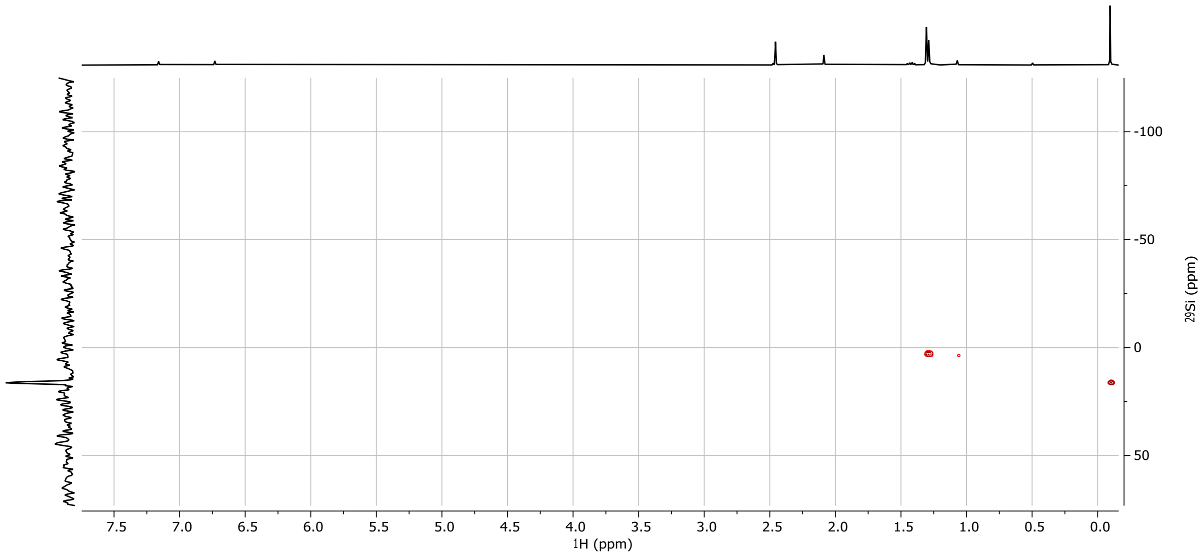


**Figure S23.** H-$^{29}Si$ HMBC NMR spectrum (80 MHz, $C_6D_6$) of MesP=C(Si$^i$Pr$_3$)OSiMe$_3$ (**3a**).

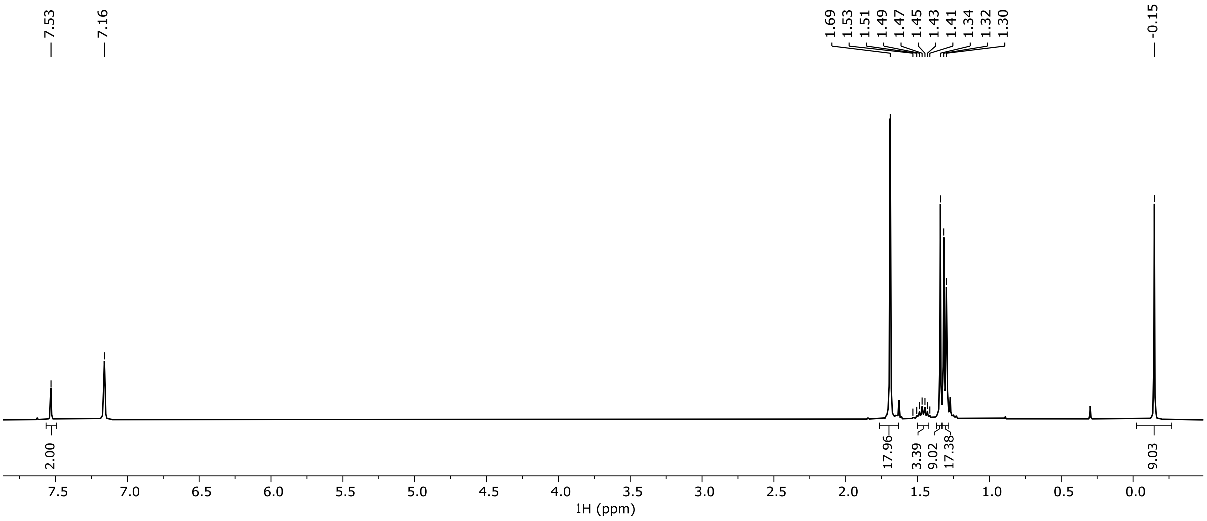


**Figure S24.** $^{1}H$ NMR spectrum (400 MHz, $C_6D_6$) of Mes*P=C(Si$^{i}$Pr$_3$)OSiMe$_3$ (**3b**).

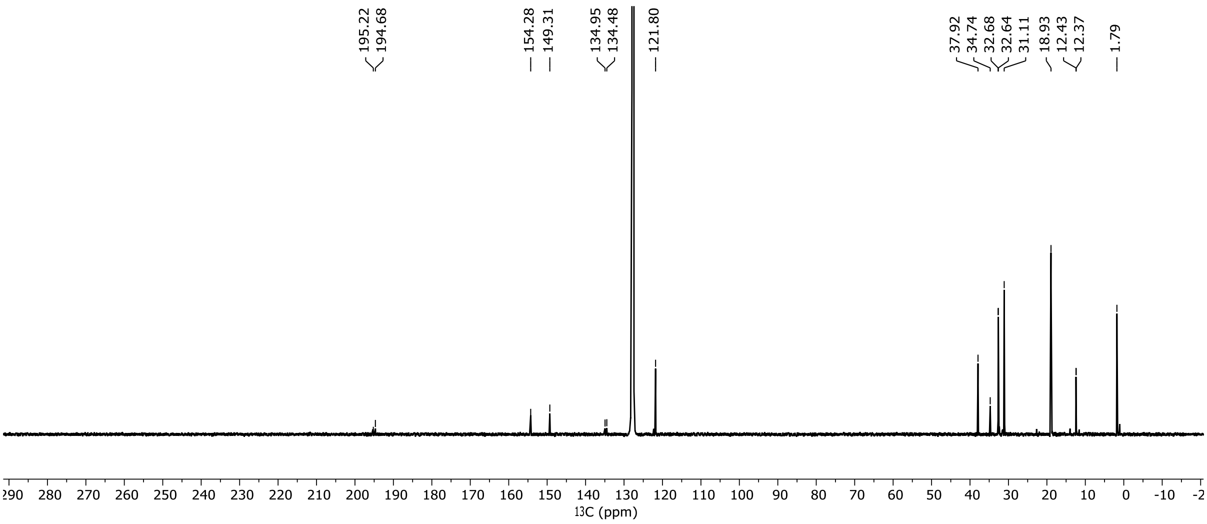


**Figure S25.** $^{13}C$ NMR spectrum (151 MHz, $C_6D_6$) of Mes*P=C(Si$^{i}$Pr$_3$)OSiMe$_3$ (**3b**).

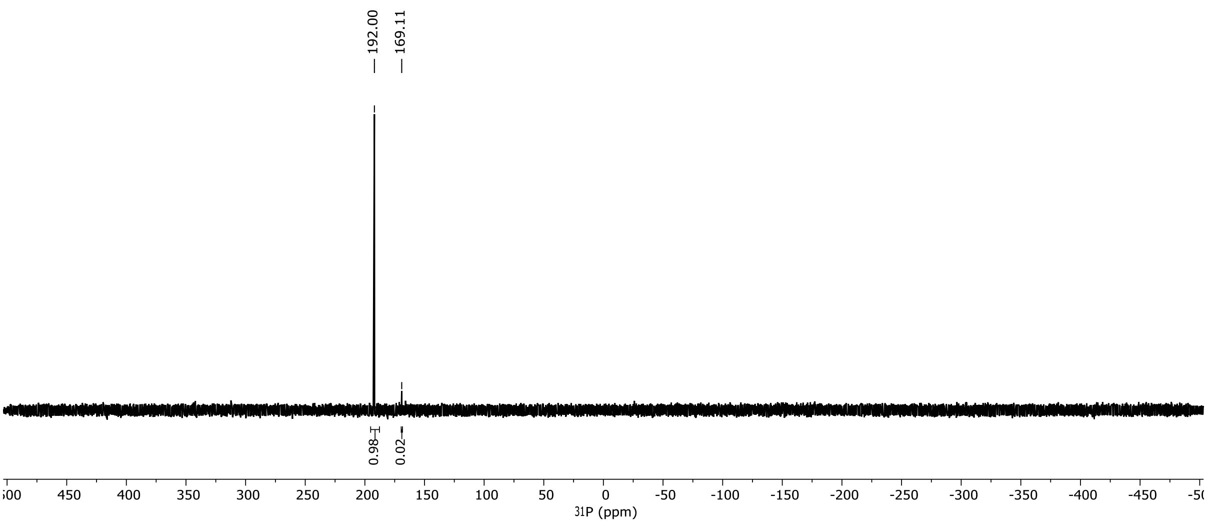


**Figure S26.** $^{31}P$ NMR spectrum (162 MHz, $C_6D_6$) of Mes*P=C(Si$^{i}$Pr$_3$)OSiMe$_3$ (**3b**). Hydrolysis product **3c** at δ = 169.11 present in trace amounts.

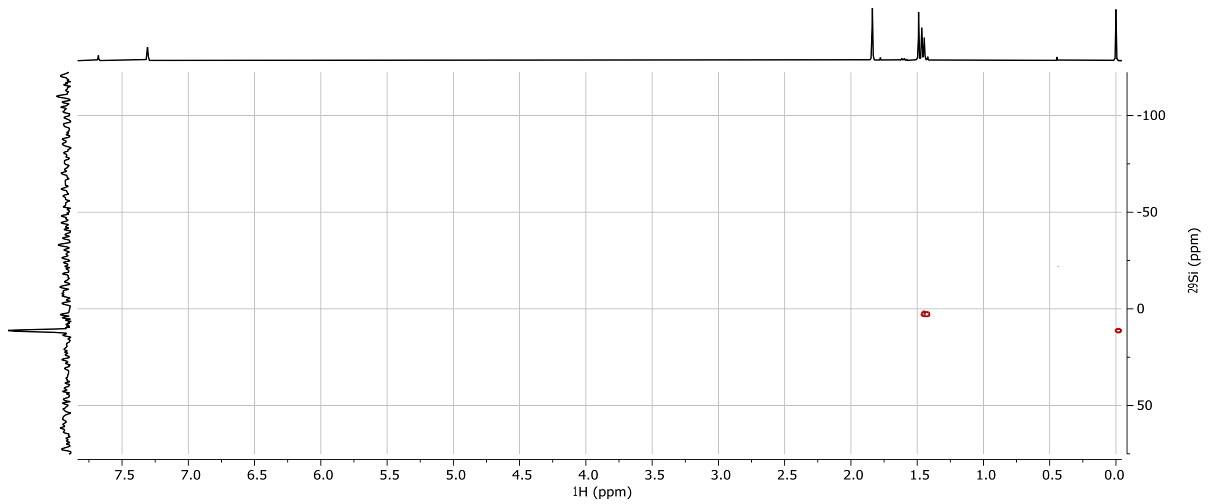


**Figure S27.** H-$^{29}$Si HMBC NMR spectrum (80 MHz, $C_6D_6$) of Mes*P=C(Si$^i$Pr$_3$)OSiMe$_3$ (**3b**).

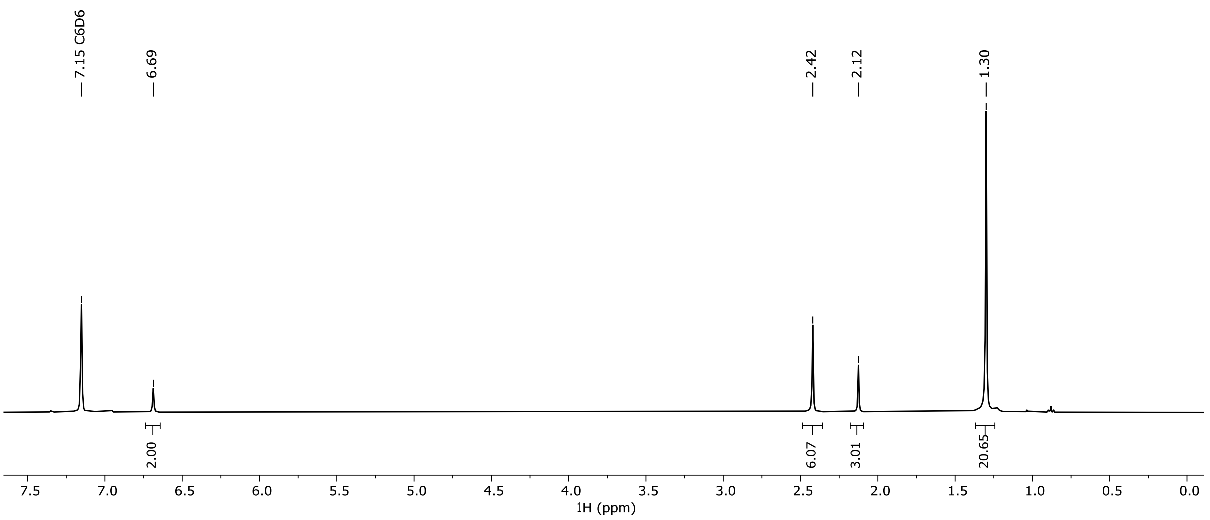


**Figure S28.** $^1$H NMR spectrum (400 MHz, $C_6D_6$) of [MesP=C(Si$^i$iPr$_3$)OK]$_2$ (**4**).

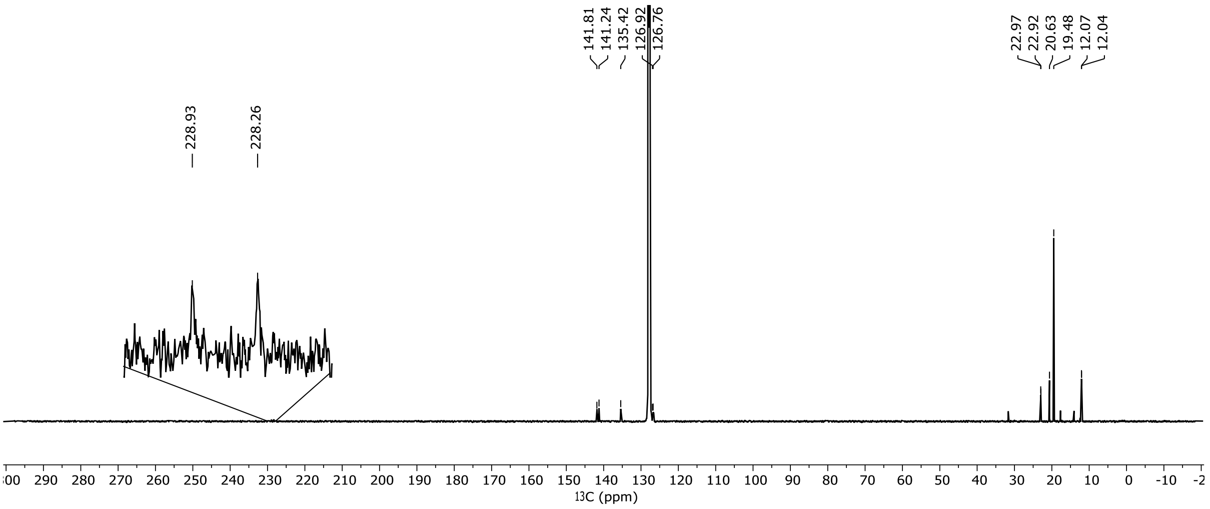


**Figure S29.** $^{13}$C NMR spectrum (101 MHz, $C_6D_6$) of [MesP=C(Si$^i$iPr$_3$)OK]$_2$ (**4**).

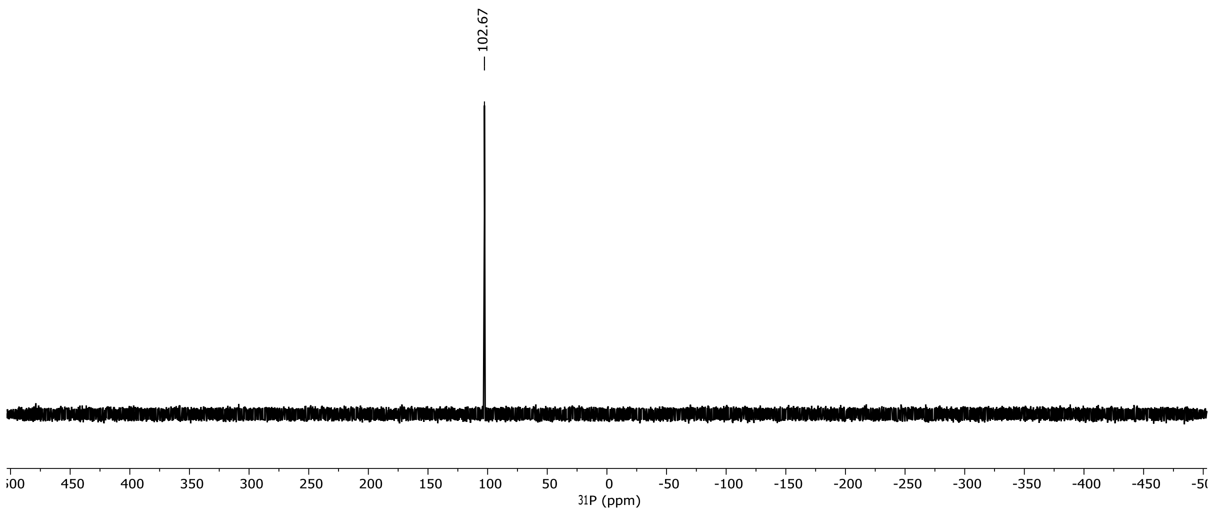


**Figure S30.** $^{31}P$ NMR spectrum (162 MHz, $C_6D_6$) of [MesP=C(S$^i$iPr$_3$)OK]$_2$ (**4**).

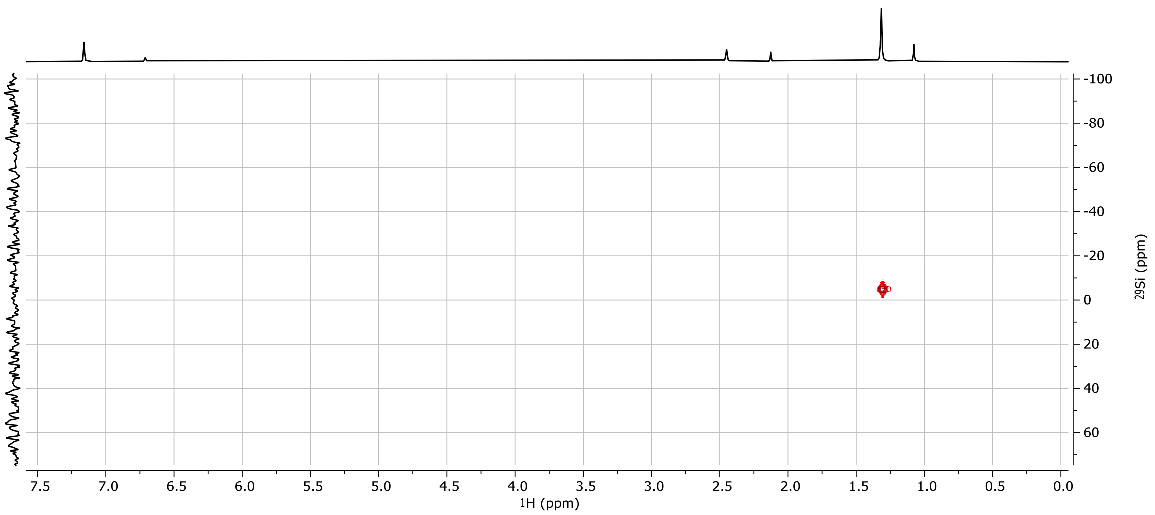


**Figure S31** H-$^{29}Si$ HMBC NMR spectrum (80 MHz, $C_6D_6$) of [MesP=C(S$^i$iPr$_3$)OK]$_2$ (**4**).

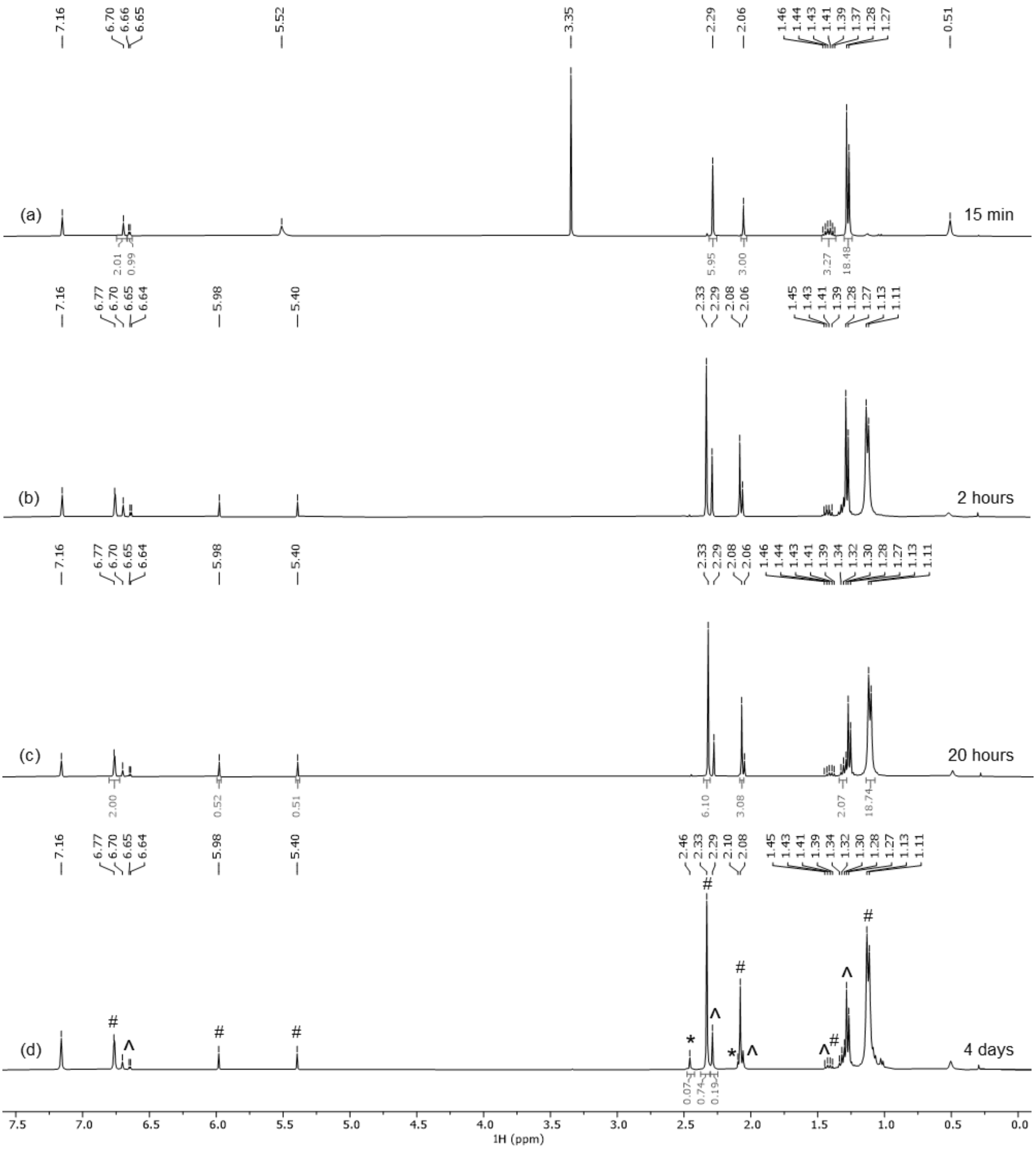


**Figure S32.** $^1$H NMR spectrum (400 MHz, $C_6D_6$) of MesP=C(Si$^i$Pr$_3$)OH. (a) Immediately after addition of $H_2O$ with integrals showing MesP=C(Si$^i$Pr$_3$)(OH) assignments, peak at 3.35 due to residual dioxane, excess water present at 0.51, (b) the volatiles were removed and the residue extracted back into $C_6D_6$, (c) same sample 20 hours after addition of $H_2O$ with integrals showing Mes(H)PC(Si$^i$Pr$_3$)(O) assignments and (d) sample 4 days later with ortho-$CH_3$ integrals showing ratio of three products present. (^) denotes MesP=C(Si$^i$Pr$_3$)(OH), ($^{\#}$) denotes Mes(H)PC(Si$^i$Pr$_3$)(O) and (*) denotes **6**.

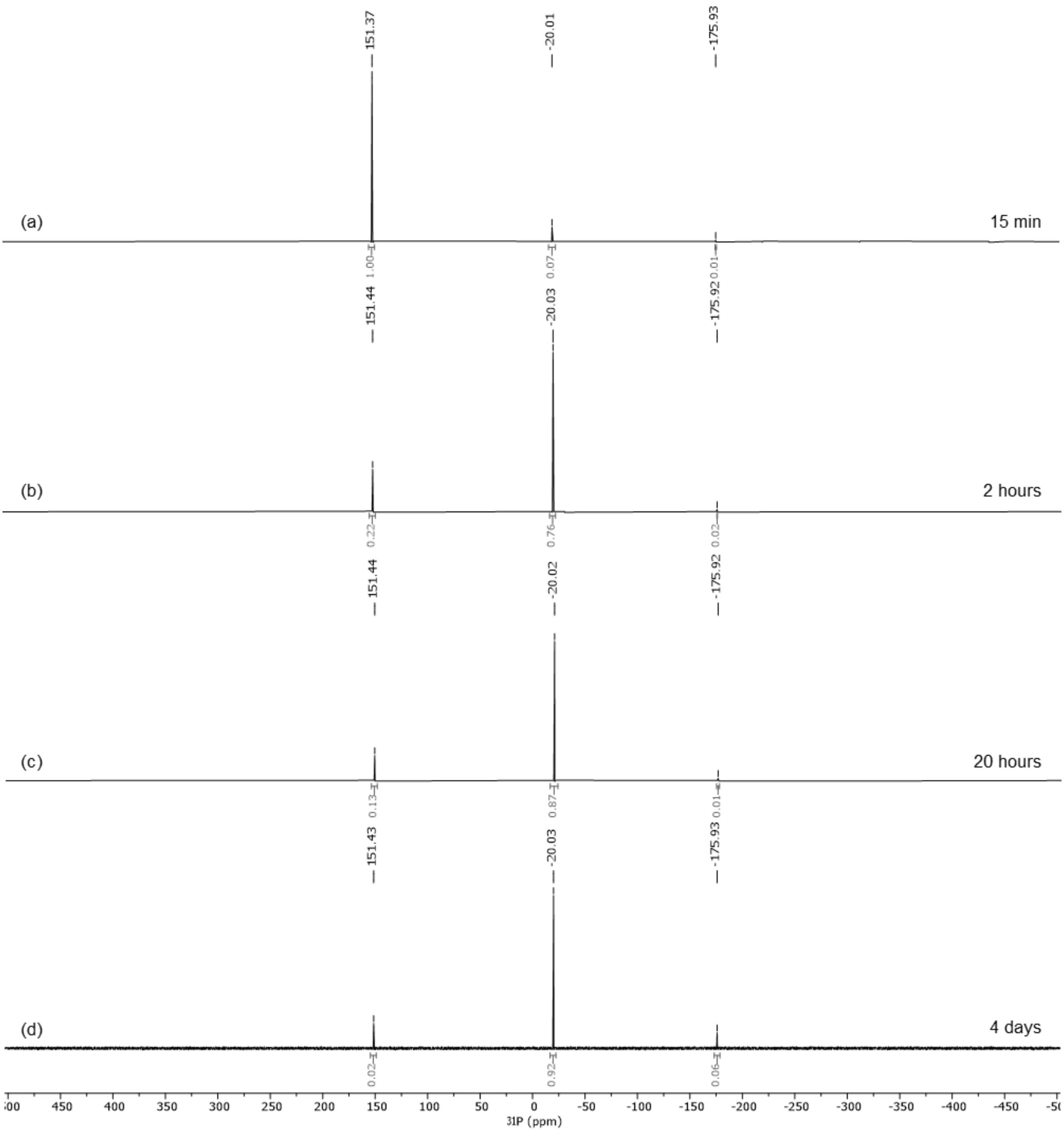


**Figure S33.** $^{31}$P NMR spectrum (162 MHz, $C_6D_6$) of MesP=C(Si$^i$Pr$_3$)OH. (a) Immediately after addition of $H_2O$, (b) the volatiles were removed and the residue extracted back into $C_6D_6$, (c) same sample 20 hours after addition of $H_2O$ and (d) same sample 4 days later.

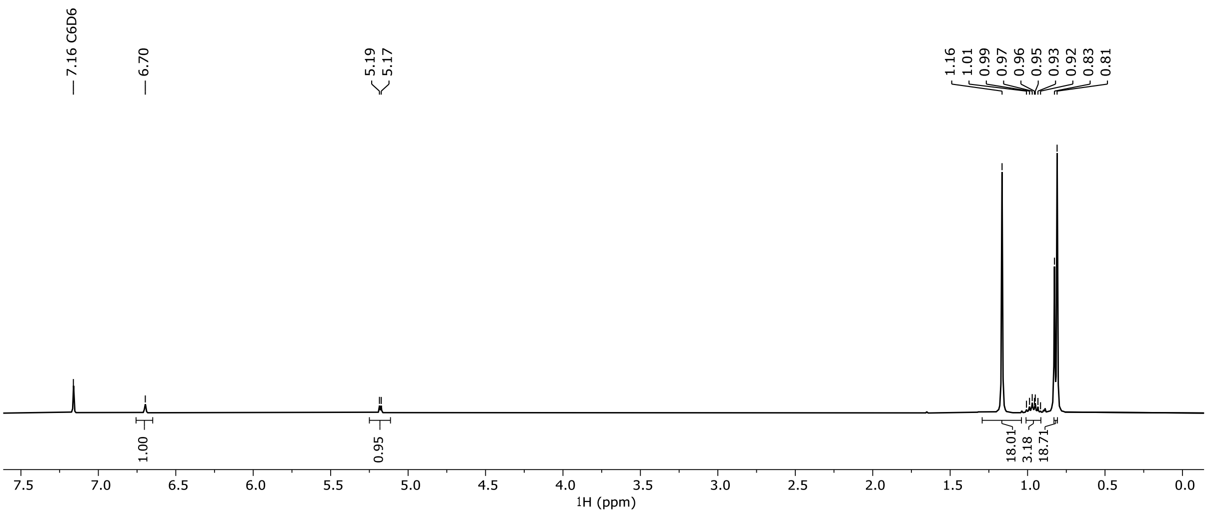


**Figure S34.** $^{1}$H NMR spectrum (400 MHz, $C_6D_6$) of Mes*P=C(Si$^{i}$Pr$_3$)OH (**5b**).

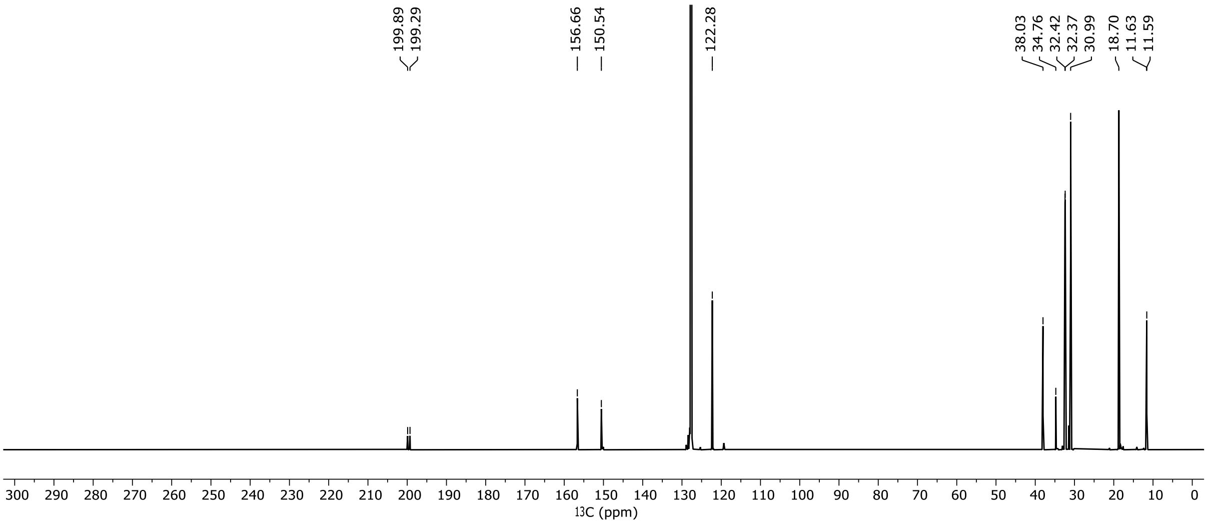


**Figure S35.** $^{13}$C NMR spectrum (101 MHz, $C_6D_6$) of Mes*P=C(Si$^{i}$Pr$_3$)OH (**5b**).

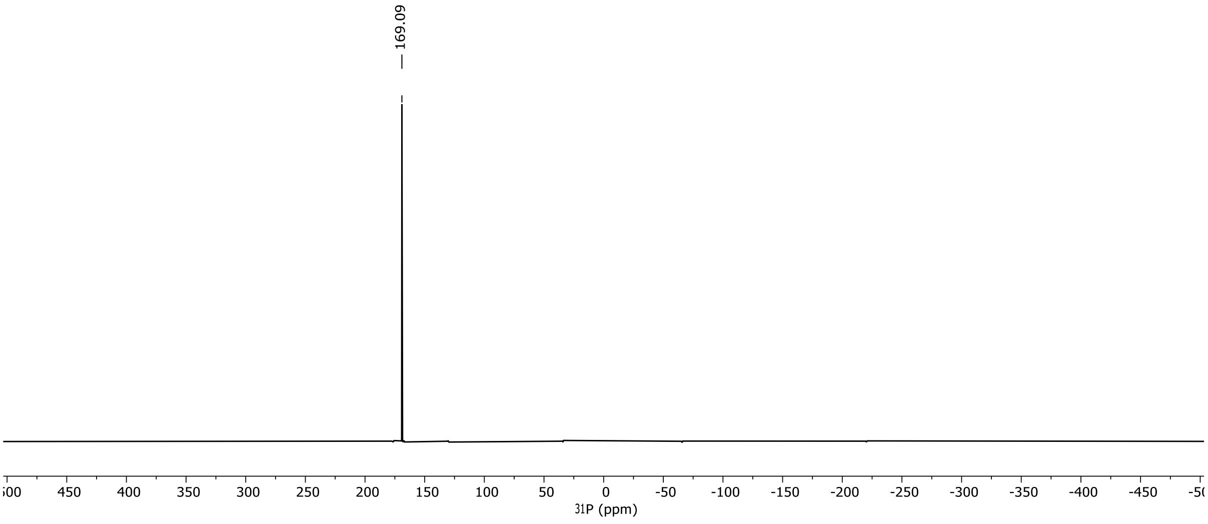


**Figure S36.** $^{31}$P NMR spectrum (162 MHz, $C_6D_6$) of Mes*P=C(Si$^{i}$Pr$_3$)OH (**5b**).

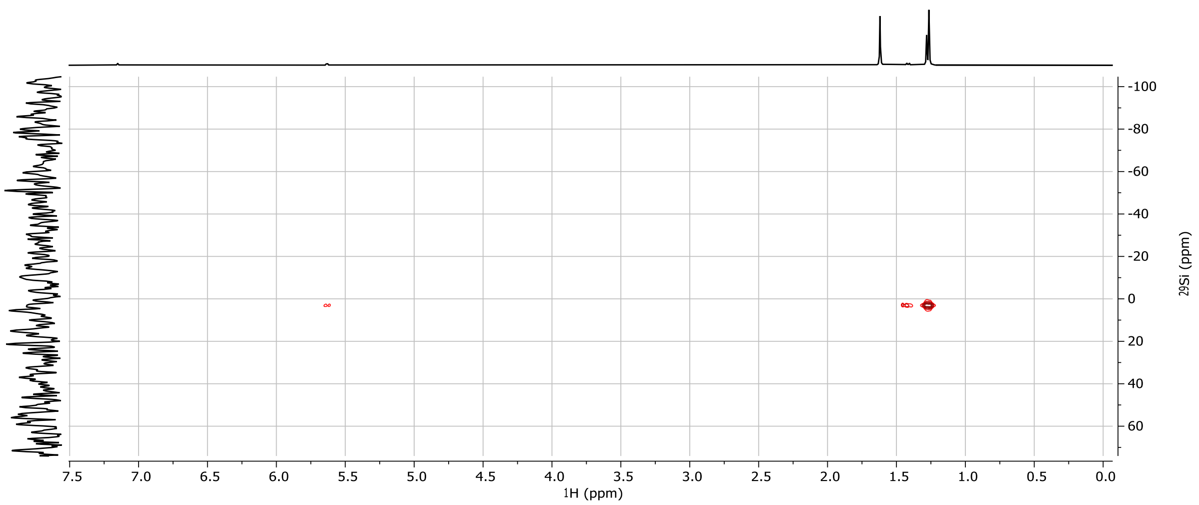


**Figure S37.** H-$^{29}$Si HMBC NMR spectrum (80 MHz, $C_6D_6$) of Mes*P=C(Si$^i$Pr$_3$)OH (**5b**).

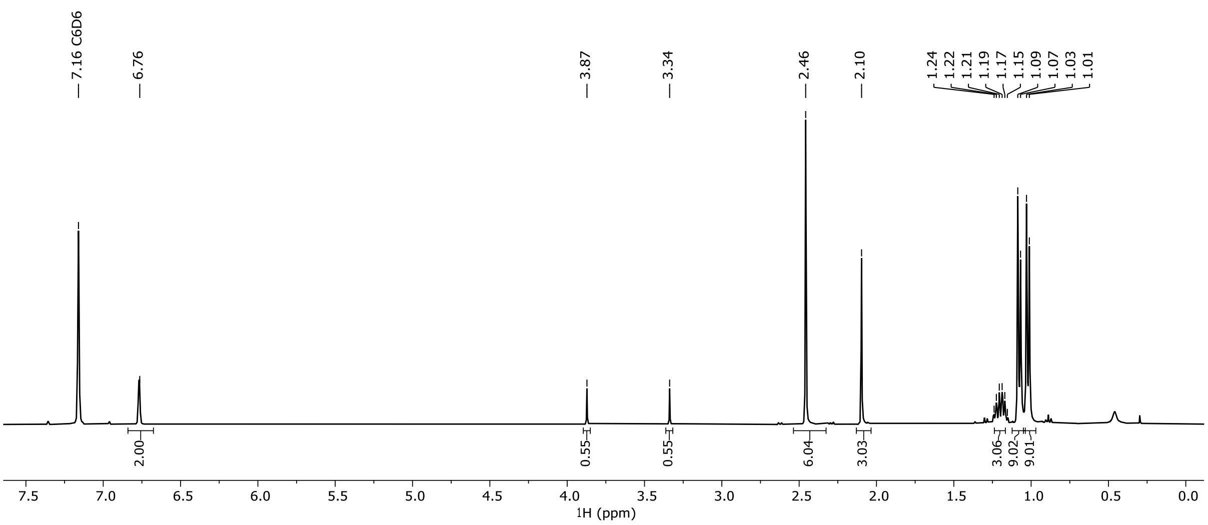


**Figure S38.** $^{1}$H NMR spectrum (400 MHz, $C_6D_6$) of MesP(H)(Si$^i$Pr$_3$) (**6**).

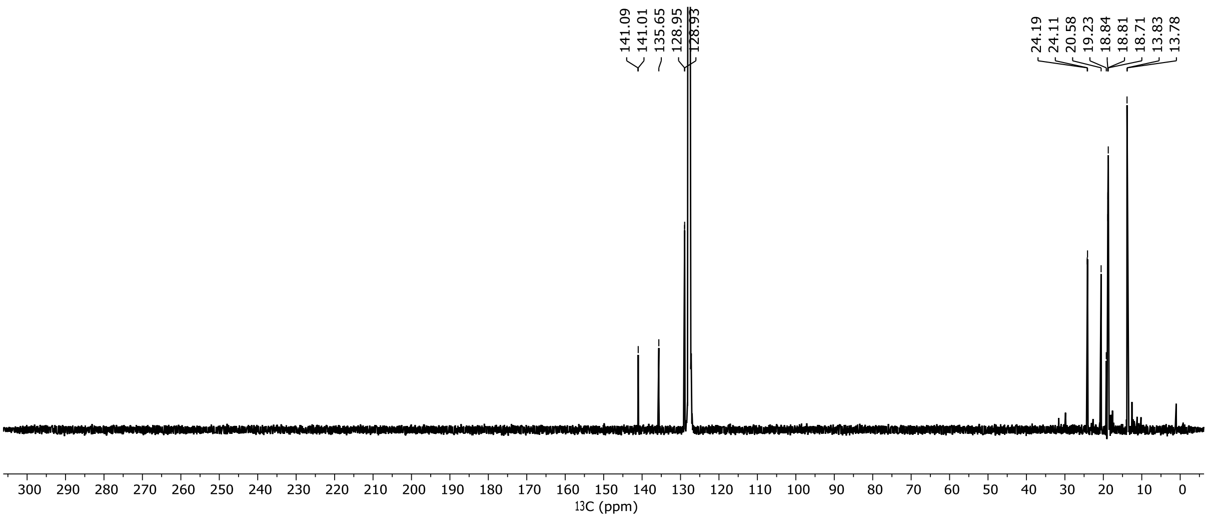


**Figure S39.** $^{13}$C NMR spectrum (151 MHz, $C_6D_6$) of MesP(H)(Si$^i$Pr$_3$) (**6**).

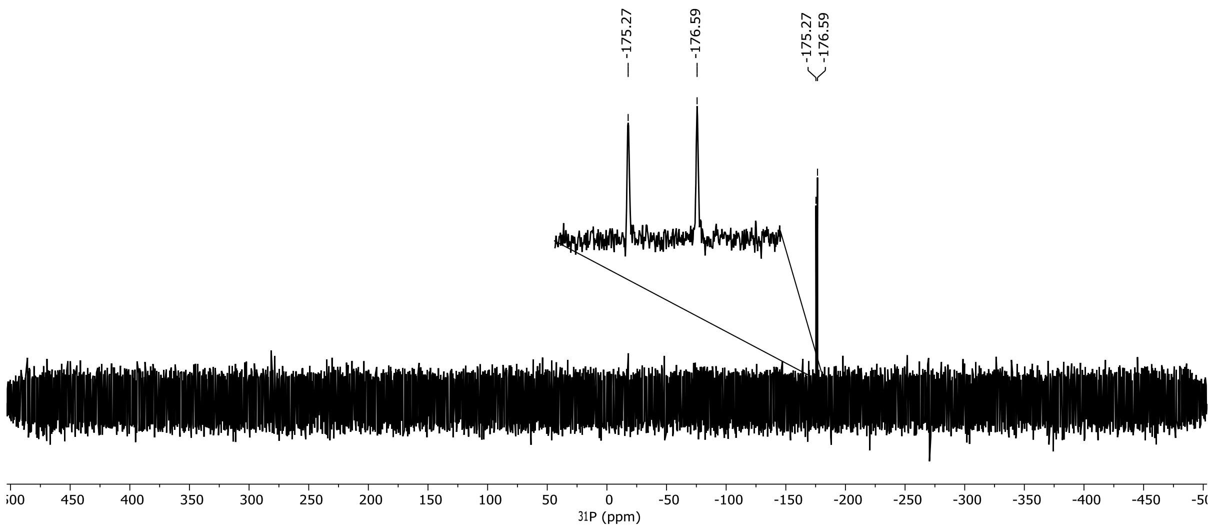


**Figure S40.** $^{31}P$ NMR spectrum (162 MHz, $C_6D_6$) of MesP(H)(Si$^i$Pr$_3$) (**6**).

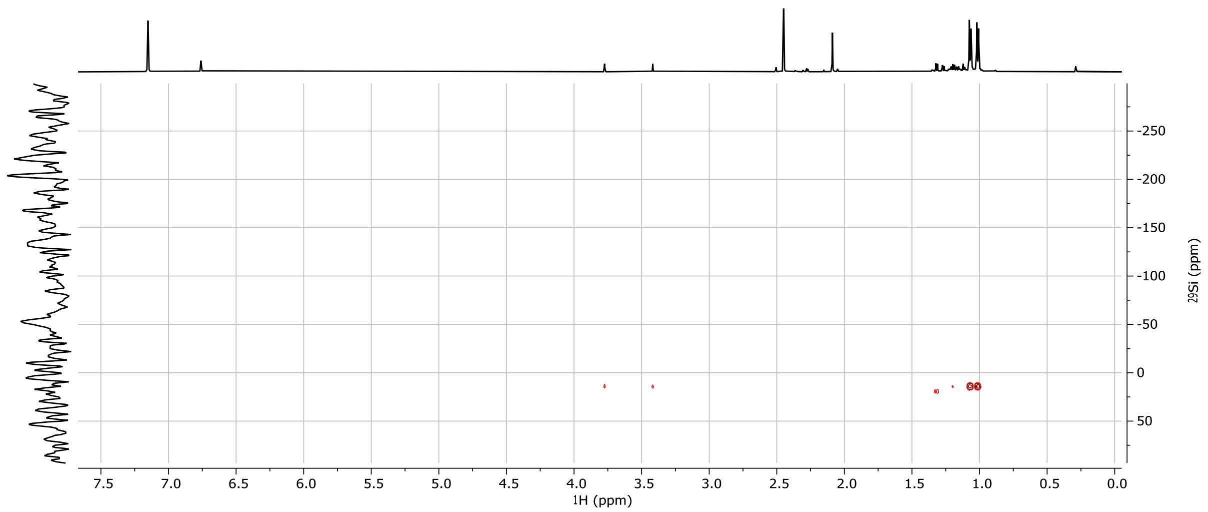


**Figure S41.** H-$^{29}$Si HMBC NMR spectrum (80 MHz, $C_6D_6$) of MesP(H)(Si$^i$Pr$_3$) (**6**).

## 2. Crystallographic Data

Single-crystal X-ray diffraction data were collected using an Oxford Diffraction Supernova dual-source diffractometer equipped with a 135 mm Atlas CCD area detector. Crystals were selected under Paratone-N oil, mounted on micromount loops and quench-cooled using an Oxford Cryosystems open flow $N_2$ cooling device. Data were collected at 150 K using mirror monochromated Cu Kα (λ = 1.54184 Å) radiation and processed using the CrysAlisPro package, including unit cell parameter refinement and inter-frame scaling (which was carried out using SCALE3 ABSPACK within CrysAlisPro).[1] Equivalent reflections were merged and diffraction patterns processed with the CrysAlisPro suite. Structures were subsequently solved using direct methods.[2]

**Table S1.** Selected X-ray data collection and refinement parameters.

| | **1a** | **1b** | **2b**·$C_6H_6$ |
|---|---|---|---|
| Formula | $C_{19}H_{32}LiOPSi$ | $C_{28}H_{50}LiOPSi$ | $C_{42}H_{72}LiO_5PSi$ |
| CCDC | 2203941 | 2203942 | 2203943 |
| Fw (g mol$^{-1}$) | 342.44 | 468.68 | 722.99 |
| Crystal colour | Pale yellow | Colourless | Colourless |
| Crystal habit | Plate | Block | Block |
| Crystal system | Triclinic | Monoclinic | Monoclinic |
| Space group | *P*–1 | $P2_1/c$ | *Cc* |
| *a* (Å) | 8.2029(4) | 11.8542(3) | 17.7658(3) |
| *b* (Å) | 10.8157(6) | 16.6683(4) | 12.2511(1) |
| *c* (Å) | 12.5252(8) | 15.4512(4) | 21.3269(4) |
| α (°) | 70.052(5) | 90 | 90 |
| β (°) | 89.885(4) | 107.019(2) | 109.596(2) |
| γ (°) | 81.217(4) | 90 | 90 |
| *V* (Å$^3$) | 1030.82(11) | 2919.19(13) | 4372.96(13) |
| Z | 2 | 4 | 4 |
| $\rho_{calc}$ (g cm$^{-3}$) | 1.103 | 1.066 | 1.098 |
| μ (mm$^{-1}$) | 1.725 | 1.330 | 1.116 |
| Reflections collected | 9224 | 15934 | 24964 |
| Independent reflections | 4246 | 6052 | 7486 |
| Parameters | 217 | 329 | 610 |
| R(int) | 0.0268 | 0.0267 | 0.0312 |
| R1/wR2, I≥ 2σI | 0.0316,0.0413 | 0.0326, 0.0385 | 0.0342, 0.0398 |
| R1/wR2, all data | 0.0740, 0.0796 | 0.0854, 0.0907 | 0.0852, 0.0878 |
| GOF | 1.047 | 1.043 | 1.032 |

[a] R1 = [Σ||Fo| – |Fc||]/Σ|Fo|; wR2 = $\{[\Sigma w[(Fo)^2 - (Fc)^2]2]/[\Sigma w(Fo^2)^2\}^{1/2}$; w = $[\sigma^2(Fo)^2 + (AP)^2 + BP]^{-1}$, where P = $[(Fo)^2 + 2(Fc)^2]/3$ and the A and B values are 0.0363 and 0.07 for **1a**, 0.0465 and 0.58 for **1b**, and 0.0488 and 1.09 for **2b**·$C_6H_6$.

**Table S2.** Selected X-ray data collection and refinement parameters.

| | **3b** | **4** | **6** |
|---|---|---|---|
| Formula | $C_{31}H_{59}OPSi_2$ | $C_{19}H_{32}KOPSi$ | $C_{18}H_{31}PSi$ |
| CCDC | 2203944 | 2203945 | 2203946 |
| Fw (g mol$^{-1}$) | 534.93 | 374.60 | 306.49 |
| Crystal colour | Colourless | Yellow | Colourless |
| Crystal habit | Block | Needle | Plate |
| Crystal system | Triclinic | Monoclinic | Monoclinic |
| Space group | *P*–1 | $P2_1/c$ | $P2_1/c$ |
| *a* (Å) | 9.5682(3) | 13.25780(10) | 15.6039(2) |
| *b* (Å) | 11.6198(4) | 10.13390(10) | 7.57808(10) |
| *c* (Å) | 15.8813(5) | 16.0979(2) | 16.6313(2) |
| α (°) | 73.989(3) | 90 | 90 |
| β (°) | 83.216(2) | 98.5060(10) | 102.6260(10) |
| γ (°) | 86.224(2) | 90 | 90 |
| *V* (Å$^3$) | 1684.29(10) | 2139.01(4) | 1917.21(4) |
| Z | 2 | 4 | 4 |
| $\rho_{calc}$ (g cm$^{-3}$) | 1.055 | 1.163 | 1.062 |
| μ (mm$^{-1}$) | 1.538 | 3.419 | 1.771 |
| Reflections collected | 17086 | 11591 | 47490 |
| Independent reflections | 6952 | 4412 | 4018 |
| Parameters | 334 | 217 | 194 |
| R(int) | 0.0184 | 0.0298 | 0.0417 |
| R1/wR2, I≥ 2σI | 0.0307, 0.0323 | 0.0310, 0.0382 | 0.0349, 0.0396 |
| R1/wR2, all data | 0.0817, 0.0833 | 0.0737, 0.0780 | 0.1027, 0.1069 |
| GOF | 1.034 | 1.048 | 1.087 |

[a] $R1 = [\Sigma||Fo| - |Fc||]/\Sigma|Fo|$; $wR2 = \{[\Sigma w[(Fo)^2 - (Fc)^2]2]/[\Sigma w(Fo^2)^2]\}^{1/2}$; $w = [\sigma^2(Fo)^2 + (AP)^2 + BP]^{-1}$, where $P = [(Fo)^2 + 2(Fc)^2]/3$ and the A and B values are 0.0449 and 0.41 for **3b**, 0.0338 and 0.31 for **4**, and 0.0623 and 0.77 for **6**.

## 3. DFT Calculations

### Geometry Optimisations

All calculations were carried out using Orca 5.0.1.[3] All methods were used as implemented. Geometry optimisations were performed using the Karlsruhe split valence basis set (def2-SVP) with the general Weigend J auxiliary basis set (def2/J).[4] Single crystal X-ray structures were used as the input structure, and were iteratively optimised until stable minima were located without negative vibrational frequencies. A comparison between experimental and optimised structures (Table S4) shows little deviation in the key bond metrics. Natural bond order (NBO) analysis was carried out on the geometry optimised structures using NBO 7.

**Table S3.** Comparison of key bond metrics in experimental and DFT optimised structures.

| | | | | | | | | |
|---|---|---|---|---|---|---|---|---|
| | | Li1-O1 | C1-O1 | P1-C1 | P1-C2 | Li1···C2 | P1-C1-O1 | Li1-O1-Li1 |
| **1b** | Experimental | 1.896(2) | 1.323(1) | 1.712(1) | 1.881(1) | 2.423(2) | 126.31(9) | 84.8(1) |
| | Optimised | 1.901 | 1.309 | 1.721 | 1.893 | 2.385 | 126.9 | 85.2 |
| | | Li1-O1 | C1-O1 | P1-C1 | P1-C2 | Li1···C2 | P1-C1-O1 | C1-O1-L1 |
| **2b** | Experimental | 1.7713 | 1.2789 | 1.7311 | 1.8780 | 4.344 | 129.52 | 173.43 |
| | Optimised | 1.784 | 1.285 | 1.739 | 1.881 | 4.465 | 130.8 | 162.01 |
| | | C1-O1 | C1-P1 | P1-C2 | C1-Si1 | O1-Si2 | P1-C1-O1 | C1-O1-Si2 |
| **3b** | Experimental | 1.352(1) | 1.687(1) | 1.857(1) | 1.911(1) | 1.638(1) | 129.20(8) | 160.10(9) |
| | Optimised | 1.346 | 1.701 | 1.861 | 1.936 | 1.708 | 133.7 | 146.5 |
| | | K1-O1 | C1-O1 | P1-C1 | P1-C2 | K1···C2 | P1-C1-O1 | K1-O1-K1 |
| **4** | Experimental | 2.635(1) | 1.291(2) | 1.738(1) | 1.852(1) | 3.056(1) | 128.3(1) | 95.97(4) |
| | Optimised | 2.603 | 1.291 | 1.744 | 1.856 | 3.065 | 129.1 | 91.5 |

Without a single crystal structure, the structure of hydrolysis products **5** were optimised from a user-generated input until a stable minimum was located without negative vibrational frequencies (Figure S42), including **5b'** which is not observed experimentally. The comparative final Gibbs free energy values for the optimised structures are detailed in Table S5.

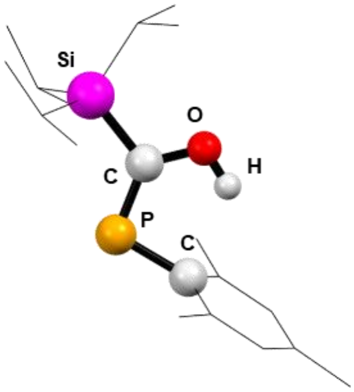


**5a**

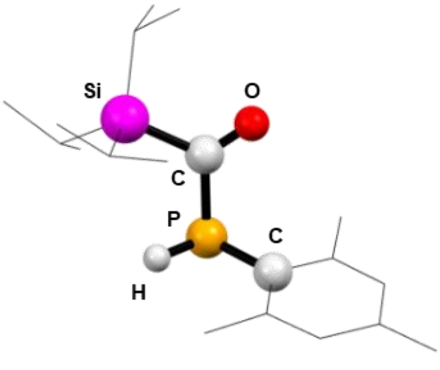


**5a’**

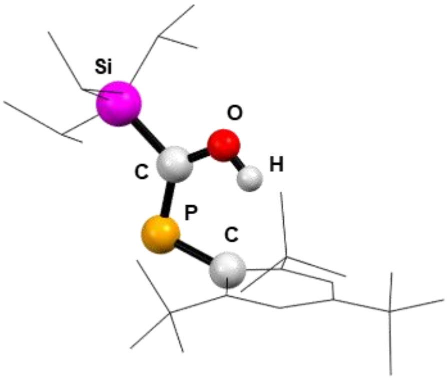


**5b**

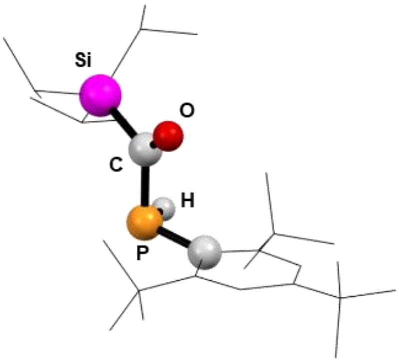


**[5b’]**

**Figure S42.** DFT optimised structures of **5b, 5b’, 5c and 5c’**, calculated at PBE0/def2-mSVP/def2/J.

**Table S4.** Calculated Gibbs Free Energy values for compounds **5.**

| **Compound** | **Gibbs Free Energy** | |
|---|---|---|
| | **(kcal mol$^{-1}$)** | **(kJ mol$^{-1}$)** |
| **5a** | 270.03 | 1129.81 |
| **5a’** | 267.49 | 1119.18 |
| **5b** | 425.89 | 1781.92 |
| [**5b’**] | 424.15 | 1774.64 |

**XYZ Coordinates of Optimised Structures**

[Mes*P=C(Si$^i$Pr$_3$)OLi]$_2$ (**1b**)

| | | | |
|---|---|---|---|
| Li | 3.99041 | -0.88659 | -3.97853 |
| Li | 1.82834 | -2.14538 | -3.70871 |
| O | 3.66118 | -2.59899 | -3.47111 |
| O | 2.15962 | -0.43326 | -4.21767 |
| C | 3.86797 | -3.79608 | -2.98214 |
| C | 1.93856 | 0.81650 | -4.53858 |
| P | 2.67735 | -4.96829 | -2.57069 |
| P | 3.12036 | 2.02323 | -4.87104 |
| C | 0.96776 | -4.23972 | -2.94470 |
| C | 0.18951 | -3.64067 | -1.89079 |
| C | 4.82753 | 1.22055 | -4.71984 |
| C | 5.51508 | 0.77177 | -5.90263 |
| C | 0.30560 | -4.54060 | -4.18270 |
| C | 5.57444 | 1.31521 | -3.49754 |
| C | 6.87986 | 0.49009 | -5.81848 |
| C | -1.17187 | -3.41132 | -2.10491 |
| C | -1.83344 | -3.72742 | -3.28674 |
| H | -1.75856 | -2.98825 | -1.30133 |
| C | 6.94035 | 0.99426 | -3.50861 |
| C | 7.62737 | 0.61698 | -4.65252 |
| H | 7.40112 | 0.18138 | -6.71384 |
| C | -1.06151 | -4.25779 | -4.30926 |
| H | -1.55092 | -4.49522 | -5.24106 |
| C | 0.73682 | -3.24601 | -0.49399 |
| C | 1.17324 | -4.48902 | 0.29943 |
| C | -0.31780 | -2.54726 | 0.38127 |
| C | 1.88229 | -2.23453 | -0.63104 |
| C | 0.97220 | -5.21748 | -5.40965 |
| C | 1.39385 | -6.65704 | -5.07107 |
| C | 2.16579 | -4.39492 | -5.91001 |
| C | 0.02344 | -5.33523 | -6.61482 |
| C | -3.34948 | -3.55785 | -3.39383 |
| C | -3.76865 | -2.13774 | -2.99276 |
| C | -4.01214 | -4.56616 | -2.44058 |
| C | -3.86580 | -3.81947 | -4.81105 |
| C | 4.87069 | 0.61571 | -7.30578 |
| C | 4.46035 | 1.98639 | -7.87043 |
| C | 3.67311 | -0.34215 | -7.26204 |
| C | 5.84109 | 0.01282 | -8.33641 |
| C | 5.00167 | 1.80001 | -2.13808 |
| C | 3.82071 | 0.92711 | -1.69252 |
| C | 4.59678 | 3.28115 | -2.21772 |
| C | 6.02729 | 1.70854 | -0.99510 |
| C | 9.14202 | 0.40877 | -4.68759 |
| C | 9.77673 | 0.48355 | -3.29718 |
| C | 9.75820 | 1.51448 | -5.56078 |
| C | 9.48481 | -0.96031 | -5.29061 |
| Si | 5.70396 | -4.25317 | -2.60187 |
| Si | 0.09849 | 1.39460 | -4.50745 |
| C | -0.93688 | -0.02039 | -5.29571 |
| C | -0.09851 | 3.08546 | -5.38757 |
| C | -0.28942 | 1.51741 | -2.62714 |
| C | 6.15135 | -3.06071 | -1.16273 |
| C | 6.74250 | -3.78893 | -4.15170 |
| C | 5.85666 | -6.09491 | -2.09201 |
| C | -0.27025 | -0.65494 | -6.51992 |
| C | -2.37080 | 0.38899 | -5.64709 |
| H | -0.99308 | -0.79481 | -4.51271 |
| C | 0.12349 | 3.00212 | -6.90085 |
| H | 0.71491 | 3.69951 | -4.97237 |
| C | -1.41763 | 3.80573 | -5.08591 |
| H | 5.65838 | -2.12128 | -1.46232 |
| C | 7.63915 | -2.75144 | -0.98585 |
| C | 5.52621 | -3.50061 | 0.16400 |
| H | 6.87440 | -2.69656 | -4.08425 |
| C | 6.02819 | -4.07812 | -5.47450 |
| C | 8.13625 | -4.42614 | -4.16285 |
| H | 8.70987 | -4.21518 | -3.25439 |
| H | 8.72092 | -4.05997 | -5.01694 |
| H | 8.07076 | -5.51445 | -4.26633 |
| H | 5.06369 | -3.56657 | -5.53376 |
| H | 5.84393 | -5.14998 | -5.60771 |
| H | 6.63966 | -3.74319 | -6.32356 |
| H | 4.45858 | -3.72298 | 0.06372 |
| H | 5.63497 | -2.71782 | 0.92614 |
| H | 6.01383 | -4.40100 | 0.55410 |
| H | 8.08762 | -2.34690 | -1.89976 |
| H | 8.20936 | -3.64281 | -0.70117 |
| H | 7.78652 | -2.00769 | -0.19090 |
| H | -2.38075 | 1.11918 | -6.46364 |
| H | -2.90916 | 0.83100 | -4.80277 |
| H | -2.94778 | -0.48084 | -5.98955 |
| H | -0.19988 | 0.05483 | -7.35157 |
| H | -0.85620 | -1.51454 | -6.87381 |
| H | 0.74214 | -1.00610 | -6.30038 |
| H | 1.08371 | 2.53866 | -7.14852 |
| H | 0.11424 | 4.00537 | -7.34636 |
| H | -0.66890 | 2.42455 | -7.39096 |
| H | -2.28065 | 3.25347 | -5.47232 |
| H | -1.42708 | 4.79419 | -5.56337 |
| H | -1.57601 | 3.96068 | -4.01420 |
| H | 0.22973 | 0.63928 | -2.20827 |
| C | -1.76408 | 1.39831 | -2.23586 |
| C | 0.33870 | 2.76350 | -1.99613 |
| H | 1.39904 | 2.86576 | -2.25015 |
| H | 0.25704 | 2.72950 | -0.90185 |
| H | -0.16784 | 3.67633 | -2.32910 |
| H | -2.20772 | 0.46003 | -2.58333 |
| H | -2.36174 | 2.22206 | -2.64219 |
| H | -1.87486 | 1.42886 | -1.14354 |
| H | 5.03631 | -6.24323 | -1.37379 |
| C | 7.16473 | -6.44238 | -1.37145 |
| C | 5.62105 | -7.06561 | -3.25373 |
| H | 4.66840 | -6.87726 | -3.75850 |
| H | 5.60355 | -8.10104 | -2.88950 |

| | | | |
|---|---|---|---|
| H | 6.42080 | -7.00007 | -4.00039 |
| H | 7.32708 | -5.82942 | -0.47951 |
| H | 8.03557 | -6.31757 | -2.02335 |
| H | 7.15135 | -7.49149 | -1.04825 |
| H | 7.49725 | 1.07566 | -2.58769 |
| H | 9.09174 | -1.07675 | -6.30515 |
| H | 9.07958 | -1.77081 | -4.67702 |
| H | 10.57232 | -1.08487 | -5.34137 |
| H | 9.36875 | 1.48240 | -6.58320 |
| H | 10.84683 | 1.39665 | -5.60926 |
| H | 9.53824 | 2.50466 | -5.14848 |
| H | 10.85476 | 0.31053 | -3.37982 |
| H | 9.36974 | -0.27859 | -2.62342 |
| H | 9.63720 | 1.46589 | -2.83353 |
| H | 6.88625 | 2.37081 | -1.14434 |
| H | 6.39140 | 0.68674 | -0.83788 |
| H | 5.53515 | 2.02462 | -0.06973 |
| H | 3.80218 | 3.46060 | -2.94580 |
| H | 5.45971 | 3.89549 | -2.49604 |
| H | 4.24069 | 3.61783 | -1.23705 |
| H | 2.99374 | 0.94289 | -2.40141 |
| H | 3.43412 | 1.28404 | -0.73099 |
| H | 4.14393 | -0.11180 | -1.53914 |
| H | 5.30125 | -0.11480 | -9.28006 |
| H | 6.21056 | -0.97388 | -8.03497 |
| H | 6.69598 | 0.66501 | -8.54326 |
| H | 4.06464 | 1.86195 | -8.88525 |
| H | 5.32929 | 2.65107 | -7.92304 |
| H | 3.69342 | 2.47593 | -7.26599 |
| H | 2.62789 | -4.89259 | -6.77049 |
| H | 2.93532 | -4.27358 | -5.14943 |
| H | 1.83474 | -3.40368 | -6.24760 |
| H | 0.58233 | -5.77951 | -7.44465 |
| H | -0.34381 | -4.36081 | -6.95597 |
| H | -0.83295 | -5.98853 | -6.41797 |
| H | 1.98731 | -5.03436 | -0.18322 |
| H | 0.32848 | -5.17581 | 0.41966 |
| H | 1.51152 | -4.18788 | 1.29785 |
| H | 2.71602 | -2.61955 | -1.21733 |
| H | 2.26911 | -1.96910 | 0.35958 |
| H | 1.51957 | -1.30820 | -1.09627 |
| H | -0.70620 | -1.63179 | -0.07943 |
| H | 0.15815 | -2.26005 | 1.32442 |
| H | -1.15843 | -3.20227 | 0.63290 |
| H | -3.73307 | -5.59231 | -2.70102 |
| H | -5.10349 | -4.48132 | -2.49781 |
| H | -3.71351 | -4.39005 | -1.40229 |
| H | -3.40380 | -3.14485 | -5.54073 |
| H | -4.94753 | -3.65275 | -4.84258 |
| H | -3.68384 | -4.85115 | -5.12981 |
| H | -3.32709 | -1.39562 | -3.66467 |
| H | -3.47063 | -1.89178 | -1.96929 |
| H | -4.85846 | -2.04055 | -3.05123 |
| H | 1.79384 | -7.14316 | -5.96846 |
| H | 0.53045 | -7.23584 | -4.72607 |
| H | 2.16097 | -6.69628 | -4.29451 |
| H | 3.25454 | -0.45486 | -8.26878 |
| H | 2.87807 | 0.01916 | -6.61212 |
| H | 3.98596 | -1.33807 | -6.92276 |

Mes*P=C(Si$^{i}$Pr$_3$)OLi(12-crown-4) (**2b**)

| | | | |
|---|---|---|---|
| C | -3.03848 | 1.23784 | -0.83754 |
| C | -4.15558 | 0.41072 | -0.49412 |
| C | -3.92024 | -0.91424 | -0.11613 |
| C | -1.75335 | 0.62415 | -0.94053 |
| C | -1.60202 | -0.71096 | -0.53790 |
| C | -2.65596 | -1.49635 | -0.09363 |
| H | -4.76281 | -1.53735 | 0.15550 |
| H | -0.62085 | -1.15998 | -0.58902 |
| C | -5.64075 | 0.84779 | -0.59163 |
| C | -5.95669 | 1.29127 | -2.03032 |
| C | -5.98845 | 1.96504 | 0.40058 |
| C | -6.61534 | -0.30291 | -0.28870 |
| C | -0.49362 | 1.31040 | -1.53042 |
| C | -0.78078 | 1.82548 | -2.95140 |
| C | 0.69180 | 0.34205 | -1.68364 |
| C | 0.00669 | 2.44477 | -0.62690 |
| C | -2.49149 | -2.95944 | 0.32430 |
| C | -3.07423 | -3.17552 | 1.72910 |
| P | -3.27933 | 3.05646 | -1.25505 |
| C | -3.08400 | 3.96063 | 0.21793 |
| O | -2.81145 | 3.57255 | 1.41271 |
| Si | -3.36063 | 5.86615 | -0.03724 |
| C | -5.09218 | 6.30677 | 0.70004 |
| C | -3.39613 | 6.32101 | -1.91061 |
| C | -1.93198 | 6.82288 | 0.83630 |
| H | -4.02193 | 5.53637 | -2.35980 |
| C | -2.02800 | 6.25068 | -2.59331 |
| C | -4.05122 | 7.66929 | -2.22349 |
| H | -1.53685 | 5.28702 | -2.42517 |
| H | -1.36273 | 7.04709 | -2.23824 |
| H | -2.13674 | 6.38280 | -3.67809 |
| H | -5.10143 | 7.70322 | -1.91830 |
| H | -4.02028 | 7.86346 | -3.30410 |
| H | -3.52924 | 8.50155 | -1.73407 |
| C | -2.10060 | 7.01026 | 2.34621 |
| H | -1.92945 | 7.82145 | 0.37030 |
| C | -0.56767 | 6.17387 | 0.57700 |
| H | -0.53876 | 5.17038 | 1.01491 |
| H | 0.23542 | 6.76511 | 1.03893 |
| H | -0.33654 | 6.07687 | -0.48664 |
| H | -3.03380 | 7.51288 | 2.61412 |
| H | -1.27406 | 7.61218 | 2.75069 |
| H | -2.07462 | 6.04040 | 2.85142 |
| H | -5.78248 | 5.99032 | -0.09857 |
| C | -5.34617 | 7.79615 | 0.96181 |
| C | -5.45837 | 5.50232 | 1.95119 |
| H | -5.38390 | 4.42564 | 1.77651 |
| H | -6.48922 | 5.72607 | 2.26169 |
| H | -4.80216 | 5.74787 | 2.79430 |
| H | -5.10990 | 8.43147 | 0.10487 |
| H | -4.75449 | 8.15885 | 1.80840 |
| H | -6.40155 | 7.96438 | 1.21658 |
| H | -4.13479 | -2.90900 | 1.77673 |
| H | -2.53825 | -2.57141 | 2.47048 |
| H | -2.98189 | -4.22767 | 2.02284 |
| C | -1.02565 | -3.39801 | 0.35333 |
| C | -3.24019 | -3.85611 | -0.67427 |
| H | -0.43430 | -2.78718 | 1.04468 |
| H | -0.56276 | -3.33972 | -0.63721 |
| H | -0.95980 | -4.43940 | 0.68680 |
| H | -4.30797 | -3.61853 | -0.70514 |
| H | -3.13615 | -4.91176 | -0.39612 |
| H | -2.83944 | -3.72547 | -1.68457 |
| H | -1.09810 | 1.00042 | -3.59848 |
| H | 0.13138 | 2.26139 | -3.37635 |
| H | -1.55918 | 2.59176 | -2.96505 |
| H | 0.87667 | 2.93123 | -1.08486 |
| H | 0.32103 | 2.03978 | 0.34254 |
| H | -0.76133 | 3.19942 | -0.45722 |
| H | 0.45382 | -0.51053 | -2.32891 |
| H | 1.05714 | -0.03570 | -0.72203 |
| H | 1.52033 | 0.88572 | -2.14961 |
| H | -5.74917 | 0.47902 | -2.73550 |
| H | -5.37106 | 2.16492 | -2.32745 |
| H | -7.01882 | 1.55227 | -2.11219 |
| H | -5.43723 | 2.87759 | 0.17339 |
| H | -5.75167 | 1.66509 | 1.42709 |
| H | -7.06162 | 2.18606 | 0.34275 |
| Li | -2.94619 | 3.22837 | 3.15851 |
| O | -1.68547 | 1.61681 | 3.75507 |
| O | -4.27586 | 1.52615 | 3.54225 |
| O | -1.31676 | 4.19610 | 4.29799 |
| O | -3.97493 | 3.91209 | 4.82010 |
| C | -4.90596 | 1.67116 | 4.79311 |
| C | -5.15985 | 3.15134 | 4.98996 |
| H | -4.26229 | 1.27671 | 5.59209 |
| H | -5.86094 | 1.12730 | 4.83158 |
| H | -5.84614 | 3.51606 | 4.22209 |
| H | -5.60500 | 3.33759 | 5.97632 |
| C | -3.56273 | 0.31070 | 3.34313 |
| C | -3.07612 | 3.91698 | 5.91432 |
| C | -1.88414 | 4.74038 | 5.47308 |
| C | -0.24542 | 3.28269 | 4.47385 |
| C | -0.42211 | 2.17445 | 3.46303 |
| H | -0.40725 | 2.57582 | 2.44159 |
| H | 0.37907 | 1.42886 | 3.56716 |
| H | -0.26767 | 2.85340 | 5.48366 |
| H | 0.71359 | 3.79467 | 4.32883 |
| C | -2.18560 | 0.66190 | 2.82740 |
| H | -4.08782 | -0.31971 | 2.61999 |
| H | -3.47834 | -0.22690 | 4.29595 |
| H | -1.54902 | -0.23194 | 2.79523 |
| H | -2.24111 | 1.10085 | 1.82386 |
| H | -6.48558 | -1.15297 | -0.96731 |
| H | -7.63657 | 0.06875 | -0.42225 |
| H | -6.53559 | -0.66023 | 0.74477 |
| H | -2.76109 | 2.89612 | 6.17024 |
| H | -3.55014 | 4.37164 | 6.79598 |

| | | | |
|---|---|---|---|
| H | -2.21620 | 5.75025 | 5.21645 |
| H | -1.14641 | 4.81243 | 6.28143 |

Mes*P=C(Si$^i$Pr$_3$)OSiMe$_3$ (**3b**)

| | | | |
|---|---|---|---|
| C | -2.98228 | 1.26569 | -0.66831 |
| C | -4.14369 | 0.44367 | -0.53525 |
| C | -3.96570 | -0.87374 | -0.10993 |
| C | -1.69773 | 0.65528 | -0.65113 |
| C | -1.60655 | -0.67828 | -0.22813 |
| C | -2.71360 | -1.44494 | 0.11119 |
| H | -4.83889 | -1.49365 | 0.04816 |
| H | -0.62917 | -1.13484 | -0.16591 |
| C | -5.57207 | 0.87478 | -0.96248 |
| C | -5.53075 | 1.33684 | -2.43104 |
| C | -6.19048 | 1.97105 | -0.08294 |
| C | -6.56149 | -0.30085 | -0.92244 |
| C | -0.40389 | 1.32439 | -1.18431 |
| C | -0.65671 | 1.86789 | -2.60139 |
| C | 0.74577 | 0.31339 | -1.32698 |
| C | 0.12466 | 2.44263 | -0.27689 |
| C | -2.60777 | -2.87655 | 0.63848 |
| C | -3.30066 | -2.95863 | 2.00763 |
| P | -3.18910 | 3.09263 | -0.95395 |
| C | -2.91326 | 3.95272 | 0.48795 |
| O | -2.52144 | 3.59262 | 1.72479 |
| Si | -3.16375 | 5.86547 | 0.32412 |
| C | -4.92116 | 6.25235 | 1.01192 |
| C | -3.13548 | 6.36156 | -1.53320 |
| C | -1.75347 | 6.75503 | 1.27945 |
| H | -3.79663 | 5.63231 | -2.02300 |
| C | -1.76061 | 6.24579 | -2.19612 |
| C | -3.71660 | 7.75471 | -1.79773 |
| H | -1.30923 | 5.25961 | -2.04714 |
| H | -1.06735 | 7.00211 | -1.81043 |
| H | -1.84619 | 6.41004 | -3.27794 |
| H | -4.77193 | 7.82535 | -1.51846 |
| H | -3.64661 | 7.99539 | -2.86643 |
| H | -3.17009 | 8.53735 | -1.25680 |
| C | -1.97005 | 6.88358 | 2.78949 |
| H | -1.73601 | 7.77171 | 0.85437 |
| C | -0.38124 | 6.11731 | 1.03028 |
| H | -0.34711 | 5.10676 | 1.44995 |
| H | 0.40637 | 6.70530 | 1.51964 |
| H | -0.12750 | 6.04515 | -0.03033 |
| H | -2.89280 | 7.40982 | 3.04695 |
| H | -1.13944 | 7.43826 | 3.24590 |
| H | -2.00318 | 5.89802 | 3.26260 |
| H | -5.57803 | 5.98242 | 0.16913 |
| C | -5.18862 | 7.72326 | 1.35240 |
| C | -5.32402 | 5.37264 | 2.19805 |
| H | -5.26140 | 4.30755 | 1.95688 |
| H | -6.35778 | 5.58503 | 2.50074 |
| H | -4.68458 | 5.55723 | 3.06847 |
| H | -4.91663 | 8.41076 | 0.54760 |
| H | -4.63772 | 8.03160 | 2.24609 |
| H | -6.25450 | 7.87359 | 1.56838 |
| H | -4.35652 | -2.67822 | 1.94229 |
| H | -2.81933 | -2.28983 | 2.72915 |
| H | -3.24777 | -3.98071 | 2.39992 |
| C | -1.15561 | -3.32870 | 0.80868 |
| C | -3.29722 | -3.84178 | -0.33809 |
| H | -0.60920 | -2.68432 | 1.50586 |
| H | -0.61905 | -3.33953 | -0.14600 |
| H | -1.13427 | -4.34705 | 1.21083 |
| H | -4.35711 | -3.60218 | -0.46710 |
| H | -3.23010 | -4.86998 | 0.03570 |
| H | -2.82157 | -3.80415 | -1.32356 |
| H | -0.99932 | 1.06855 | -3.26688 |
| H | 0.27363 | 2.27924 | -3.00949 |
| H | -1.40401 | 2.66694 | -2.62021 |
| H | 1.03235 | 2.87128 | -0.71822 |
| H | 0.38617 | 2.04985 | 0.70977 |
| H | -0.59641 | 3.24866 | -0.14616 |
| H | 0.47657 | -0.53371 | -1.96656 |
| H | 1.08960 | -0.07005 | -0.36042 |
| H | 1.59781 | 0.82107 | -1.79068 |
| H | -5.12373 | 0.54675 | -3.07060 |
| H | -4.92385 | 2.23520 | -2.57061 |
| H | -6.54607 | 1.56664 | -2.77434 |
| H | -5.58598 | 2.87987 | -0.05818 |
| H | -6.32453 | 1.61638 | 0.94331 |
| H | -7.17795 | 2.23630 | -0.47829 |
| H | -6.24242 | -1.13253 | -1.55919 |
| H | -7.52974 | 0.04765 | -1.29602 |
| H | -6.72510 | -0.67629 | 0.09349 |
| Si | -2.63436 | 2.43169 | 2.97189 |
| C | -1.16452 | 1.26666 | 2.94825 |
| C | -4.24490 | 1.47464 | 2.93896 |
| C | -2.54683 | 3.44902 | 4.55182 |
| H | -1.61108 | 4.01265 | 4.62217 |
| H | -2.59591 | 2.78408 | 5.42207 |
| H | -3.37679 | 4.15810 | 4.63004 |
| H | -4.30170 | 0.79594 | 2.08443 |
| H | -5.11494 | 2.13743 | 2.91940 |
| H | -4.30857 | 0.87250 | 3.85369 |
| H | -1.17207 | 0.60554 | 2.07785 |
| H | -1.19795 | 0.64126 | 3.84928 |
| H | -0.21702 | 1.81442 | 2.96451 |

[MesP=C(Si$^i$Pr$_3$)OK]$_2$ (**4**)

| | | | |
|---|---|---|---|
| K | 4.44611 | -0.74255 | -3.08169 |
| K | 1.27970 | -2.27367 | -4.36023 |
| O | 3.48097 | -3.12593 | -3.21798 |
| O | 2.26363 | 0.13284 | -4.24239 |
| C | 3.80097 | -4.35516 | -2.98525 |
| C | 1.97975 | 1.33546 | -4.61677 |
| P | 2.75390 | -5.71898 | -2.69709 |
| P | 3.05493 | 2.67362 | -4.92381 |
| C | 1.08019 | -4.92947 | -2.84366 |
| C | 0.59452 | -4.02891 | -1.86771 |
| C | 4.71379 | 1.90731 | -4.59798 |
| C | 5.29197 | 1.00177 | -5.51708 |
| C | 0.25635 | -5.23479 | -3.95059 |
| C | 5.43927 | 2.24233 | -3.43298 |
| C | 6.53767 | 0.42780 | -5.24058 |
| C | -0.65581 | -3.42347 | -2.04018 |
| C | -1.46658 | -3.69682 | -3.14497 |
| H | -1.01164 | -2.72713 | -1.28226 |
| C | 6.68376 | 1.64728 | -3.18997 |
| C | 7.25450 | 0.73545 | -4.08130 |
| H | 6.96482 | -0.27207 | -5.95729 |
| C | -0.98944 | -4.61023 | -4.08803 |
| H | -1.60801 | -4.85334 | -4.95072 |
| C | 1.40773 | -3.69485 | -0.64944 |
| H | 1.68011 | -4.60521 | -0.10545 |
| H | 0.85289 | -3.03632 | 0.02574 |
| H | 2.34457 | -3.20329 | -0.93374 |
| C | 0.70737 | -6.20740 | -5.00751 |
| H | 0.87569 | -7.20047 | -4.57777 |
| H | 1.66213 | -5.90317 | -5.45041 |
| H | -0.03843 | -6.29967 | -5.80317 |
| C | -2.82428 | -3.06639 | -3.28675 |
| H | -2.84354 | -2.05229 | -2.87526 |
| H | -3.57974 | -3.65031 | -2.74772 |
| H | -3.13701 | -3.01500 | -4.33411 |
| C | 4.57790 | 0.62668 | -6.78485 |
| H | 4.34639 | 1.51765 | -7.37758 |
| H | 3.62243 | 0.14058 | -6.56122 |
| H | 5.18364 | -0.05217 | -7.39286 |
| C | 4.88638 | 3.21956 | -2.43033 |
| H | 3.90639 | 2.90145 | -2.05732 |
| H | 4.73205 | 4.20237 | -2.88794 |
| H | 5.56563 | 3.33866 | -1.58060 |
| C | 8.61243 | 0.14235 | -3.82784 |
| H | 8.83974 | 0.09993 | -2.75826 |
| H | 9.39272 | 0.74770 | -4.30419 |
| H | 8.69323 | -0.87079 | -4.23481 |
| Si | 5.70298 | -4.67611 | -2.89470 |
| Si | 0.08926 | 1.71997 | -4.73309 |
| C | -0.72317 | 0.22664 | -5.65258 |
| C | -0.26089 | 3.40706 | -5.56771 |
| C | -0.46029 | 1.66298 | -2.89350 |
| C | 6.21377 | -3.79767 | -1.25743 |
| C | 6.48351 | -3.72050 | -4.37767 |
| C | 6.12802 | -6.54257 | -2.86469 |
| C | 0.11163 | -0.28818 | -6.83067 |
| C | -2.16489 | 0.46773 | -6.11094 |
| H | -0.77443 | -0.56566 | -4.88502 |
| C | 0.08200 | 3.40610 | -7.06128 |
| H | 0.44493 | 4.09291 | -5.07541 |
| C | -1.67334 | 3.95618 | -5.34021 |
| H | 5.62144 | -2.86540 | -1.27299 |
| C | 7.68440 | -3.39449 | -1.13188 |
| C | 5.74967 | -4.59138 | -0.03280 |
| H | 6.49219 | -2.66239 | -4.06314 |
| C | 5.64240 | -3.80693 | -5.65505 |
| C | 7.93656 | -4.10161 | -4.67763 |
| H | 8.58664 | -4.01254 | -3.80162 |
| H | 8.35099 | -3.45861 | -5.46618 |
| H | 8.00772 | -5.13346 | -5.03771 |
| H | 4.61305 | -3.48213 | -5.47645 |
| H | 5.60651 | -4.83265 | -6.03789 |
| H | 6.06953 | -3.17676 | -6.44719 |
| H | 4.69007 | -4.86120 | -0.10381 |
| H | 5.89385 | -4.01429 | 0.89000 |
| H | 6.31945 | -5.52125 | 0.07452 |
| H | 8.00553 | -2.73908 | -1.94973 |
| H | 8.34624 | -4.26713 | -1.12955 |
| H | 7.85983 | -2.85550 | -0.19092 |
| H | -2.20285 | 1.22328 | -6.90261 |
| H | -2.81417 | 0.80822 | -5.29895 |
| H | -2.60161 | -0.45266 | -6.52185 |
| H | 0.10437 | 0.43126 | -7.65600 |
| H | -0.29565 | -1.23040 | -7.22706 |
| H | 1.16387 | -0.44161 | -6.56567 |
| H | 1.10740 | 3.06657 | -7.23934 |
| H | -0.01034 | 4.41751 | -7.47779 |
| H | -0.59831 | 2.76114 | -7.63046 |
| H | -2.43770 | 3.32279 | -5.80325 |
| H | -1.76955 | 4.95466 | -5.78637 |
| H | -1.91873 | 4.04950 | -4.27740 |
| H | 0.11486 | 0.80645 | -2.50383 |
| C | -1.94026 | 1.38233 | -2.62795 |
| C | 0.00707 | 2.90734 | -2.13285 |
| H | 1.07218 | 3.10603 | -2.29704 |
| H | -0.15665 | 2.79270 | -1.05324 |
| H | -0.54565 | 3.79908 | -2.45050 |
| H | -2.26882 | 0.43930 | -3.07980 |
| H | -2.58355 | 2.17822 | -3.01965 |
| H | -2.13417 | 1.31298 | -1.54917 |
| H | 5.42517 | -6.95744 | -2.12609 |
| C | 7.54713 | -6.87218 | -2.38954 |
| C | 5.83913 | -7.23469 | -4.20087 |
| H | 4.81006 | -7.06054 | -4.53102 |
| H | 5.98200 | -8.31918 | -4.10931 |
| H | 6.51701 | -6.88622 | -4.98894 |
| H | 7.75485 | -6.48025 | -1.38891 |
| H | 8.30867 | -6.47263 | -3.06793 |

| | | | |
|---|---|---|---|
| H | 7.69066 | -7.95988 | -2.34870 |
| H | 7.22762 | 1.91579 | -2.28541 |

MesP=C(Si$^i$Pr$_3$)OH (**5a**)

| | | | |
|---|---|---|---|
| C | -3.26276 | 1.22302 | -0.60075 |
| C | -4.27247 | 0.39380 | -0.06627 |
| C | -3.95532 | -0.90895 | 0.31810 |
| C | -1.95418 | 0.71353 | -0.74975 |
| C | -1.68116 | -0.59627 | -0.34843 |
| C | -2.66621 | -1.42491 | 0.18528 |
| H | -4.73890 | -1.53957 | 0.73323 |
| H | -0.66924 | -0.97927 | -0.46289 |
| C | -5.68072 | 0.89378 | 0.10553 |
| H | -6.08316 | 1.28451 | -0.83555 |
| H | -5.72844 | 1.71179 | 0.83292 |
| H | -6.34005 | 0.09333 | 0.45282 |
| C | -0.84722 | 1.55164 | -1.32915 |
| H | -1.12320 | 1.94559 | -2.31318 |
| H | 0.06844 | 0.96417 | -1.43990 |
| H | -0.62062 | 2.41435 | -0.69277 |
| C | -2.35751 | -2.84234 | 0.57574 |
| H | -2.97321 | -3.16808 | 1.41940 |
| H | -1.30662 | -2.96067 | 0.85512 |
| H | -2.55515 | -3.52683 | -0.25774 |
| P | -3.69608 | 2.92780 | -1.19576 |
| C | -3.05747 | 3.85400 | 0.08797 |
| O | -2.43262 | 3.34899 | 1.17216 |
| H | -2.44337 | 2.36940 | 1.09789 |
| Si | -3.19081 | 5.77341 | 0.07112 |
| C | -5.05598 | 6.20283 | -0.10282 |
| C | -2.27032 | 6.42040 | -1.48943 |
| C | -2.42559 | 6.48279 | 1.67239 |
| H | -2.99464 | 6.28730 | -2.30779 |
| C | -1.01496 | 5.62634 | -1.86235 |
| C | -1.93253 | 7.91363 | -1.40075 |
| H | -1.23693 | 4.56668 | -2.01617 |
| H | -0.24313 | 5.69876 | -1.08809 |
| H | -0.57991 | 6.01692 | -2.79137 |
| H | -2.80914 | 8.54032 | -1.21046 |
| H | -1.48230 | 8.25942 | -2.33997 |
| H | -1.20672 | 8.11262 | -0.60385 |
| C | -3.16365 | 6.05053 | 2.94380 |
| H | -2.54378 | 7.57356 | 1.57242 |
| C | -0.92710 | 6.19003 | 1.80508 |
| H | -0.74169 | 5.11370 | 1.86979 |
| H | -0.52889 | 6.65236 | 2.71760 |
| H | -0.35007 | 6.58347 | 0.96232 |
| H | -4.21005 | 6.37101 | 2.94523 |
| H | -2.68672 | 6.49044 | 3.82935 |
| H | -3.14110 | 4.96266 | 3.06150 |
| H | -5.28239 | 5.97132 | -1.15541 |
| C | -5.34316 | 7.69133 | 0.12189 |
| C | -5.99597 | 5.35323 | 0.75682 |
| H | -5.85222 | 4.28339 | 0.57937 |
| H | -7.04150 | 5.59298 | 0.52431 |
| H | -5.85243 | 5.53960 | 1.82587 |
| H | -4.77768 | 8.33823 | -0.55549 |
| H | -5.10363 | 7.99408 | 1.14822 |
| H | -6.40769 | 7.90487 | -0.03804 |

MesP(H)C(Si$^i$Pr$_3$)(O) (**5a'**)

| | | | |
|---|---|---|---|
| C | -2.85456 | 1.25711 | -0.75386 |
| C | -4.16769 | 0.75291 | -0.82375 |
| C | -4.42727 | -0.51890 | -0.30545 |
| C | -1.82452 | 0.46182 | -0.20663 |
| C | -2.13592 | -0.79727 | 0.30022 |
| C | -3.43402 | -1.30628 | 0.26973 |
| H | -5.44330 | -0.90395 | -0.35695 |
| H | -1.33663 | -1.40110 | 0.72498 |
| C | -5.30615 | 1.50943 | -1.45476 |
| H | -5.10902 | 1.73663 | -2.50836 |
| H | -5.50252 | 2.45985 | -0.94597 |
| H | -6.22492 | 0.91837 | -1.41328 |
| C | -0.39868 | 0.94024 | -0.17762 |
| H | -0.06733 | 1.25109 | -1.17649 |
| H | 0.27119 | 0.14774 | 0.16675 |
| H | -0.29112 | 1.79872 | 0.49156 |
| C | -3.73909 | -2.66661 | 0.82994 |
| H | -4.79085 | -2.93020 | 0.68842 |
| H | -3.52457 | -2.70835 | 1.90356 |
| H | -3.13100 | -3.43939 | 0.34711 |
| P | -2.36336 | 2.88990 | -1.42753 |
| C | -2.19220 | 3.98268 | 0.11471 |
| O | -1.59555 | 3.58873 | 1.09941 |
| Si | -2.98418 | 5.77030 | 0.09930 |
| C | -4.85866 | 5.43652 | 0.34895 |
| C | -2.72134 | 6.61236 | -1.60825 |
| C | -2.18329 | 6.76701 | 1.51493 |
| H | -3.52382 | 6.21461 | -2.24844 |
| C | -1.38689 | 6.29356 | -2.29189 |
| C | -2.90894 | 8.13254 | -1.52284 |
| H | -1.24432 | 5.22056 | -2.45707 |
| H | -0.53543 | 6.65305 | -1.70424 |
| H | -1.33572 | 6.78671 | -3.27099 |
| H | -3.87428 | 8.41912 | -1.09349 |
| H | -2.84786 | 8.58276 | -2.52169 |
| H | -2.12547 | 8.59436 | -0.91139 |
| C | -2.51717 | 6.23046 | 2.91042 |
| H | -2.60217 | 7.78201 | 1.43242 |
| C | -0.66378 | 6.87011 | 1.34018 |
| H | -0.19900 | 5.87963 | 1.38023 |
| H | -0.22712 | 7.47270 | 2.14671 |
| H | -0.38046 | 7.34006 | 0.39268 |
| H | -3.59022 | 6.26559 | 3.12359 |
| H | -2.01336 | 6.83317 | 3.67715 |
| H | -2.17937 | 5.19593 | 3.02166 |
| H | -5.20434 | 5.08525 | -0.63662 |
| C | -5.64866 | 6.70473 | 0.68990 |
| C | -5.16189 | 4.32841 | 1.36327 |
| H | -4.64726 | 3.39021 | 1.13026 |
| H | -6.23916 | 4.11983 | 1.38796 |
| H | -4.86475 | 4.61565 | 2.37650 |
| H | -5.54840 | 7.48009 | -0.07618 |
| H | -5.32162 | 7.13429 | 1.64354 |
| H | -6.71797 | 6.47731 | 0.78601 |
| H | -3.62576 | 3.41387 | -1.80185 |

Mes*P=C(Si$^{i}$Pr$_3$)OH (**5b**)

| | | | |
|---|---|---|---|
| C | -2.96946 | 1.30692 | -1.04578 |
| C | -4.13678 | 0.52610 | -0.78145 |
| C | -3.97661 | -0.69533 | -0.12603 |
| C | -1.68707 | 0.71759 | -0.85527 |
| C | -1.61447 | -0.51486 | -0.18887 |
| C | -2.73508 | -1.21588 | 0.23512 |
| H | -4.85775 | -1.27545 | 0.11592 |
| H | -0.64006 | -0.94205 | -0.00046 |
| C | -5.56362 | 0.90741 | -1.24930 |
| C | -5.54355 | 1.29292 | -2.73822 |
| C | -6.17776 | 2.04043 | -0.41395 |
| C | -6.53188 | -0.28215 | -1.13854 |
| C | -0.36227 | 1.30688 | -1.40075 |
| C | -0.53697 | 1.74964 | -2.86218 |
| C | 0.76016 | 0.25488 | -1.41446 |
| C | 0.14941 | 2.47825 | -0.55120 |
| C | -2.65248 | -2.52949 | 1.01328 |
| C | -3.39854 | -2.37360 | 2.34752 |
| P | -3.14878 | 3.11865 | -1.48671 |
| C | -2.96192 | 3.80335 | 0.06548 |
| O | -2.69694 | 3.12401 | 1.20280 |
| H | -2.62467 | 2.17071 | 0.98368 |
| Si | -3.18136 | 5.68949 | 0.35672 |
| C | -4.92673 | 5.94155 | 1.12229 |
| C | -3.15043 | 6.51217 | -1.37601 |
| C | -1.79962 | 6.30294 | 1.53855 |
| H | -3.80160 | 5.85961 | -1.97866 |
| C | -1.77793 | 6.53306 | -2.05445 |
| C | -3.76265 | 7.91625 | -1.40761 |
| H | -1.29573 | 5.55028 | -2.05107 |
| H | -1.10618 | 7.24667 | -1.56454 |
| H | -1.87644 | 6.84890 | -3.10080 |
| H | -4.80876 | 7.92187 | -1.08736 |
| H | -3.73148 | 8.31953 | -2.42785 |
| H | -3.20976 | 8.61557 | -0.76852 |
| C | -2.00617 | 5.86360 | 2.99351 |
| H | -1.86353 | 7.40244 | 1.50378 |
| C | -0.38933 | 5.89598 | 1.10007 |
| H | -0.27025 | 4.80889 | 1.15042 |
| H | 0.35767 | 6.33687 | 1.77286 |
| H | -0.14550 | 6.21357 | 0.08349 |
| H | -2.94602 | 6.22585 | 3.41880 |
| H | -1.19320 | 6.24995 | 3.62221 |
| H | -1.99890 | 4.77220 | 3.07526 |
| H | -5.60270 | 5.87621 | 0.25465 |
| C | -5.12249 | 7.32225 | 1.76262 |
| C | -5.34723 | 4.84920 | 2.11069 |
| H | -5.26631 | 3.84763 | 1.68228 |
| H | -6.38941 | 5.00030 | 2.42046 |
| H | -4.73229 | 4.86665 | 3.01599 |
| H | -4.87601 | 8.14719 | 1.08851 |
| H | -4.50409 | 7.43608 | 2.65900 |
| H | -6.16683 | 7.45249 | 2.07444 |
| H | -4.45174 | -2.11721 | 2.19673 |
| H | -2.94474 | -1.58647 | 2.95870 |
| H | -3.35898 | -3.31088 | 2.91415 |
| C | -1.20904 | -2.93447 | 1.32064 |
| C | -3.30305 | -3.65575 | 0.19541 |
| H | -0.69282 | -2.17464 | 1.91735 |
| H | -0.63103 | -3.10942 | 0.40688 |
| H | -1.20541 | -3.86653 | 1.89543 |
| H | -4.35669 | -3.44855 | -0.01529 |
| H | -3.25202 | -4.60117 | 0.74739 |
| H | -2.78858 | -3.79020 | -0.76171 |
| H | -0.87989 | 0.91477 | -3.48211 |
| H | 0.42407 | 2.09747 | -3.25746 |
| H | -1.25124 | 2.57053 | -2.97254 |
| H | 1.13591 | 2.79221 | -0.91180 |
| H | 0.25326 | 2.18496 | 0.49929 |
| H | -0.51643 | 3.33981 | -0.60657 |
| H | 0.46676 | -0.65368 | -1.95073 |
| H | 1.09218 | -0.02230 | -0.40844 |
| H | 1.62779 | 0.68038 | -1.92884 |
| H | -5.13255 | 0.47768 | -3.34273 |
| H | -4.95421 | 2.19248 | -2.93374 |
| H | -6.56571 | 1.49057 | -3.08058 |
| H | -5.63368 | 2.97911 | -0.53198 |
| H | -6.18778 | 1.77713 | 0.64936 |
| H | -7.21424 | 2.20803 | -0.72945 |
| H | -6.16440 | -1.16392 | -1.67386 |
| H | -7.48830 | 0.00497 | -1.58694 |
| H | -6.73799 | -0.56069 | -0.09971 |

Mes*P(H)C(Si$^i$Pr$_3$)(O) (**[5b']**)

| | | | |
|---|---|---|---|
| C | -3.08913 | 1.23900 | -1.00246 |
| C | -4.18920 | 0.41326 | -0.61320 |
| C | -3.90845 | -0.84067 | -0.06942 |
| C | -1.77105 | 0.69852 | -0.98521 |
| C | -1.58787 | -0.57995 | -0.44713 |
| C | -2.62273 | -1.35309 | 0.05854 |
| H | -4.72923 | -1.45766 | 0.26900 |
| H | -0.58894 | -0.98719 | -0.41350 |
| C | -5.68768 | 0.76598 | -0.81680 |
| C | -5.96739 | 1.01149 | -2.31022 |
| C | -6.13862 | 1.97249 | 0.02451 |
| C | -6.61345 | -0.39196 | -0.40588 |
| C | -0.51084 | 1.39044 | -1.55885 |
| C | -0.72437 | 1.76759 | -3.03817 |
| C | 0.71626 | 0.45939 | -1.58250 |
| C | -0.07785 | 2.57950 | -0.68563 |
| C | -2.40389 | -2.72189 | 0.70337 |
| C | -2.91576 | -2.67889 | 2.15130 |
| P | -3.49988 | 3.00308 | -1.37804 |
| C | -3.30274 | 3.86461 | 0.29906 |
| O | -3.39057 | 3.25564 | 1.34745 |
| Si | -3.20884 | 5.82631 | 0.36250 |
| C | -4.99714 | 6.42601 | 0.73840 |
| C | -2.72638 | 6.49394 | -1.37599 |
| C | -1.98922 | 6.26018 | 1.77389 |
| H | -3.31158 | 5.86012 | -2.06247 |
| C | -1.24998 | 6.34734 | -1.75489 |
| C | -3.16763 | 7.94031 | -1.62691 |
| H | -0.86815 | 5.33105 | -1.61600 |
| H | -0.62326 | 7.02065 | -1.15964 |
| H | -1.09952 | 6.61390 | -2.80868 |
| H | -4.24619 | 8.07611 | -1.50661 |
| H | -2.91232 | 8.24079 | -2.65107 |
| H | -2.66069 | 8.63940 | -0.95108 |
| C | -2.55345 | 5.91793 | 3.15892 |
| H | -1.84831 | 7.35128 | 1.71818 |
| C | -0.61682 | 5.59522 | 1.62173 |
| H | -0.70484 | 4.50537 | 1.68660 |
| H | 0.04890 | 5.91516 | 2.43342 |
| H | -0.12360 | 5.83888 | 0.67723 |
| H | -3.47336 | 6.46267 | 3.38855 |
| H | -1.82095 | 6.17161 | 3.93617 |
| H | -2.76780 | 4.84763 | 3.23423 |
| H | -5.48945 | 6.43809 | -0.24652 |
| C | -5.02766 | 7.85863 | 1.29077 |
| C | -5.81597 | 5.50593 | 1.64833 |
| H | -5.91752 | 4.49727 | 1.24168 |
| H | -6.82562 | 5.91472 | 1.78311 |
| H | -5.36538 | 5.41116 | 2.64093 |
| H | -4.47578 | 8.57207 | 0.67230 |
| H | -4.60641 | 7.90349 | 2.30028 |
| H | -6.06347 | 8.21482 | 1.35951 |
| H | -3.98086 | -2.43156 | 2.19606 |
| H | -2.37336 | -1.92708 | 2.73373 |
| H | -2.77660 | -3.65375 | 2.63300 |
| C | -0.92791 | -3.12581 | 0.72864 |
| C | -3.17746 | -3.79377 | -0.07992 |
| H | -0.32155 | -2.41404 | 1.29906 |
| H | -0.51107 | -3.20605 | -0.28114 |
| H | -0.82517 | -4.10568 | 1.20668 |
| H | -4.25281 | -3.59152 | -0.09362 |
| H | -3.02855 | -4.77796 | 0.37888 |
| H | -2.83175 | -3.84420 | -1.11767 |
| H | -0.82564 | 0.85547 | -3.63591 |
| H | 0.14599 | 2.31900 | -3.41216 |
| H | -1.60966 | 2.37263 | -3.23211 |
| H | 0.80784 | 3.05960 | -1.11851 |
| H | 0.18598 | 2.22410 | 0.31599 |
| H | -0.84559 | 3.34089 | -0.56258 |
| H | 0.53277 | -0.45397 | -2.15735 |
| H | 1.05551 | 0.18391 | -0.57888 |
| H | 1.54216 | 0.99218 | -2.06555 |
| H | -5.72661 | 0.11801 | -2.89595 |
| H | -5.39193 | 1.84963 | -2.70968 |
| H | -7.03058 | 1.23684 | -2.45338 |
| H | -5.80375 | 2.91909 | -0.40414 |
| H | -5.76115 | 1.90308 | 1.04865 |
| H | -7.23350 | 2.00864 | 0.05899 |
| H | -6.40288 | -1.31242 | -0.96013 |
| H | -7.64433 | -0.10322 | -0.63382 |
| H | -6.56587 | -0.60335 | 0.66784 |
| H | -2.29979 | 3.53143 | -1.89154 |

## 4. References


[1] CrysAlisPro, Agilent Technologies, Version 1.171.35.8.

[2] (a) G. M. Sheldrick in SHELXL97, Programs for Crystal Structure Analysis (Release 97-2), Institut für Anorganische Chemie der Universität, Tammanstrasse 4, D-3400 Göttingen, Germany, 1998; (b) G. M. Sheldrick, *Acta Crystallogr. Sect. A* **1990**, *46*, 467−473; (c) G. M. Sheldrick, *Acta Crystallogr. Sect. A* **2008**, *64*, 112−122.

[3] F. Neese, Wiley Interdiscip. Rev. Comput. Mol. Sci., 2022, 10.1002/wcms.1606.

[4] F. Weigend and R. Ahlrichs, *Phys. Chem. Chem. Phys.*, **2005**, *7*, 3297–3305.
